# Discovery of PXR Antagonist MI891 and PXR Degrader MI1013 and Their Roles in Hepatic Gene Regulation

**DOI:** 10.1021/acs.jmedchem.4c03134

**Published:** 2025-07-12

**Authors:** Rajamanikkam Kamaraj, Ivana Mejdrová, Maria Krutakova, Tomas Smutny, Kryštof Škach, Klara Dohnalova, Lucie Smutna, Dharani Sai Sreekanth Nellore, Jan Dusek, Karel Chalupsky, Jana Hricová, Thales Kronenberger, Aaron Stahl, Markus Templin, Albert Braeuning, Radim Nencka, Petr Pavek

**Affiliations:** † Department of Pharmacology and Toxicology, Faculty of Pharmacy in Hradec Kralove, Charles University, Akademika Heyrovskeho 1203, 500 05 Hradec Kralove, Czech Republic; ‡ Institute of Organic Chemistry and Biochemistry, Czech Academy of Sciences, Flemingovo nám. 2, 166 10 Prague 6, Czech Republic; § Czech Centre for Phenogenomics, Institute of Molecular Genetics of the Czech Academy of Sciences, Vídeňská 1083, 142 20 Prague, Czech Republic; ∥ First Faculty of Medicine, Charles University, Katerinska 32, 112 08 Prague, Czech Republic; ⊥ DZIF Tübingen Partner Site, University Hospital Tübingen, 72076 Tuebingen, Germany; # NMI - Natural and Medical Sciences Institute at the University of Tuebingen, Markwiesenstr. 55, 72770 Reutlingen, Germany; ∇ Department Food Safety, German Federal Institute for Risk Assessment, Max-Dohrn-Str. 8-10, Berlin 10589, Germany

## Abstract

The pregnane X receptor (PXR) is an important regulator of hepatic metabolism, yet mechanistic insights into the effects of pharmacological inhibition using PXR inverse agonists or antagonists on critical genes involved in both xenobiotic and endobiotic metabolism remain limited. Here, we discovered a novel PXR inverse agonist/antagonist, MI891, which binds to the ligand-binding domain of PXR. Furthermore, we computationally designed and synthesized the proteolysis-targeting chimera molecule, MI1013, based on the PXR antagonist SPA70, which degrades PXR in HepaRG hepatic cells. Using these tools, we investigated the regulation of key PXR target genes in HepaRG cells and human hepatocytes. Our findings indicate that PXR antagonism or degradation suppresses basal and rifampicin-induced expression of selected ADME genes. Moreover, the PXR antagonists and PROTAC degrader downregulate the expression of several key genes involved in gluconeogenesis, cholesterol homeostasis, bile acid synthesis, and proliferation in hepatocyte cells, suggesting their potential therapeutic applications for metabolic diseases.

## Graphical abstract

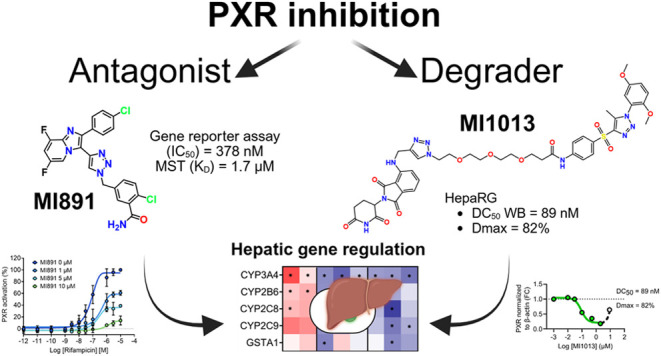

## Introduction

The PXR (NR1I2) is the crucial nuclear receptor that regulates genes involved in drug metabolism and disposition.^1^ Known as the xenobiotic receptor, PXR is activated by various xenobiotics and drugs. Its ligand-binding domain (LBD) is flexible, allowing it to interact with a wide range of ligands of different sizes and shapes. When activated, PXR acts as a transcription factor, primarily upregulating its target genes, with *CYP3A4* being the main drug-metabolizing enzyme controlled by PXR. This upregulation can lead to significant drug–drug interactions (DDIs), particularly when multiple drugs are coadministered. Additionally, PXR activation affects lipid and cholesterol metabolism, glucose tolerance, and blood pressure, potentially explaining some of the adverse metabolic effects of environmental chemicals and certain drugs.^2–4^

The ligand-binding pocket (LBP) of PXR is large (1300–1544 Å^3^) and flexible, allowing it to bind bulky or multiple ligands simultaneously.^5,6^ However, structural changes in the PXR LBP reduce binding affinity and specificity, increase ligand promiscuity, and hinder the development of selective PXR inverse agonists and antagonists.^4,7,8^

Large-scale, cell-based high-throughput screenings have been conducted to identify specific PXR antagonists.^9–11^ SPA70 (SJ000076745-1) has been discovered as a highly potent and selective PXR antagonist with 1-substituted-phenyl-4-substituted-phenylsulfonyl-5-methyl-1*H*-1,2,3-triazole scaffold (see Figure 1).^11^ However, SPA70 also exhibits weak inverse agonistic activity toward the constitutive androstane receptor (CAR), marginally increases activation of LXRβ by T0901317, and inhibits KIT kinase and CSF1R activities.^11^ Mechanistically, SPA70 and its 1*H*-1,2,3-triazole-4-carboxamide derivatives interact directly with the PXR LBP and destabilize the AF-2 helix for coactivator binding, as demonstrated by the recent crystallization of PXR with antagonists.^12^ Further chemical derivatization of the SPA70 with carbonyl amide linker led to the discovery of SJPYT-306, a selective and potent inverse agonist/antagonist of PXR, and SJPYT-310, a pure antagonist with nanomolar IC_50_ in cellular assays.^12^ However, derivatives with a sulfonyl linker resulted only in agonists and weaker antagonists.^13^ Notably, even minor chemical modifications to the compounds, as well as mutations in the PXR LBP, can dramatically alter the biological activity of these ligands, transforming SPA70-derived antagonists into agonists.^14,15^ Nevertheless, SPA70 and its novel highly potent 1*H*-1,2,3-triazole-4-carboxamide analog SJPYT-331 (see Figure 1), which is currently the most potent PXR antagonist with an IC_50_ of 7.1 ± 0.8 nM,^12^ need further pharmacokinetic optimization and safety assessment, to be used as a model PXR antagonist in *in vivo* studies.

**Figure 1:**
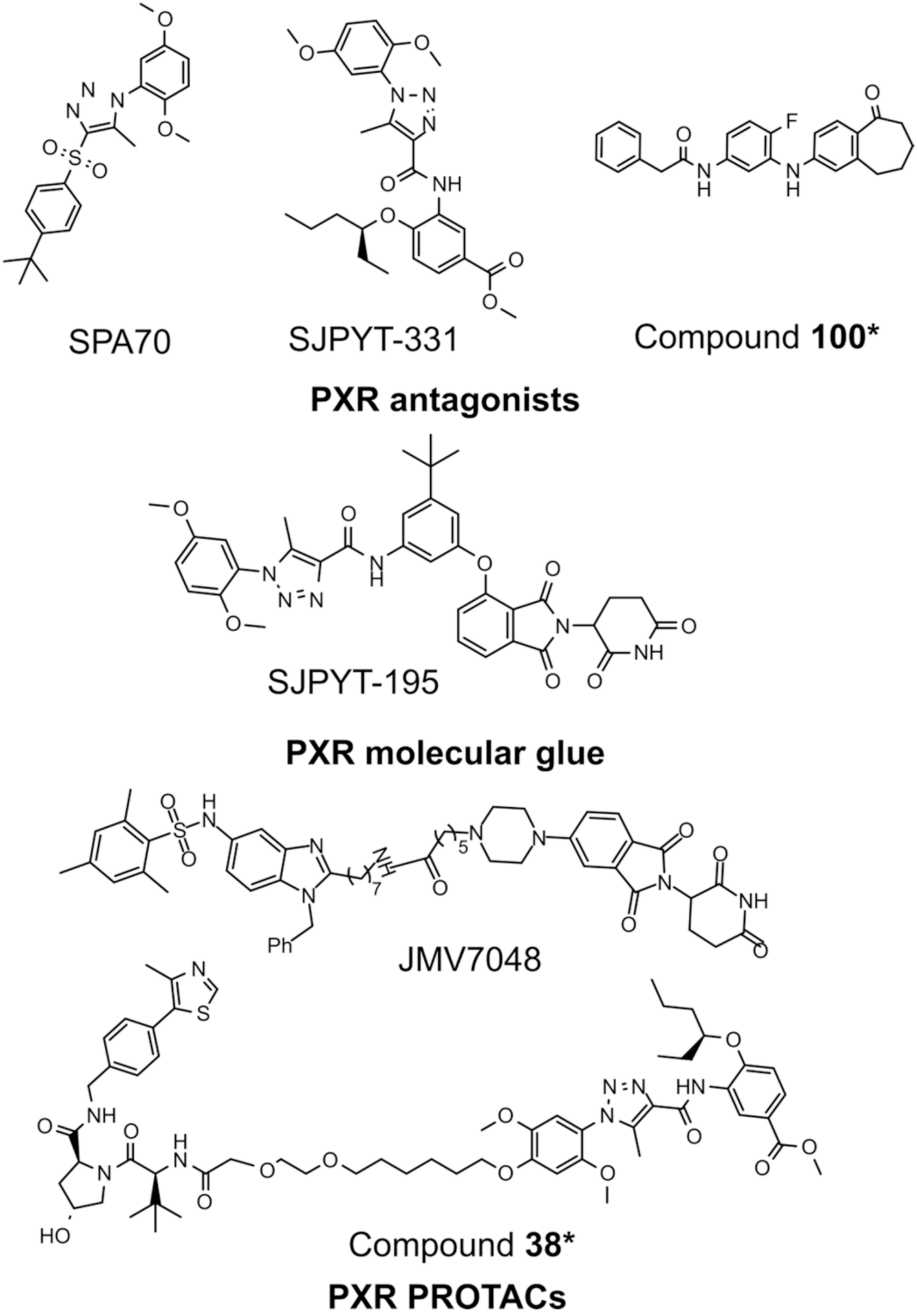
Chemical structures of currently available PXR antagonists, proved to act in the PXR ligand binding pocket, and proteolysis targeting chimeras (PROTACs).^10–12,19–21^

Additionally, in another screening of potential PXR antagonists, compound 100* has been identified as a kinase (RAF1) and PXR antagonist through its interaction with the PXR LBD in colon LS174T cancer cells; however, it exhibited partial agonist activity in phenotypically differentiated HepaRG cells.^10^ Other compounds have been identified to inhibit PXR allosterically.^16–18^

So far, the flexibility of the PXR LBP and undefined antagonist binding sites have complicated the discovery of PXR inverse agonists/antagonists at the orthosteric site.^4,8,22^ Therefore, alternative approaches, including molecular degraders, have been considered. The agonist-based PROTAC JMV7048 was the first tool to demonstrate tissue-specific PXR degradation, reducing PXR expression in colon cancer stem cells and delaying cancer relapse *in vivo*.^21,23^ A recent study demonstrated that a molecular glue targeting PXR, SJPYT-195, which consists of SPA70 and the cereblon (CRBN) ligand thalidomide, did not directly degrade PXR.^20^ Instead, it induced the degradation of GSPT1, a protein involved in translation termination. Curiously, SJPYT-195, in this way, indirectly reduced PXR protein levels in colon SNU-C4 cancer cells.^20^ Thus, ligand-unknown E3 ligases UBR5, RBCK1, FBXO44, and TRIM21, which mediate PXR ubiquitination, require further investigation.^24^ A recently discovered PXR PROTAC (compound 38*) conjugated with VHL ligand (VH032) has demonstrated an effective degradation profile of PXR.^19^ However, a comprehensive understanding of how these molecules suppress all PXR target genes remains unknown.

Thus, while PXR is recognized as a master regulator in agonist-induced up-regulation (*induction*) of numerous genes involved in drug metabolism, the intricate details of how antagonists influence PXR’s target genes remain elusive. Notably, transcriptome analysis after treatment with SPA70 has only been performed using DNA microarray in hepatocytes,^11^ and there is a lack of comprehensive transcriptomic data regarding the genes regulated by PXR inverse agonists/antagonists. This gap in understanding raises critical questions about how PXR antagonists may modulate hepatic metabolic functions connected with xenobiotic elimination and homeostasis of lipids, glucose, cholesterol, and bile acids. Addressing this gap is vital for elucidating the potential therapeutic implications of PXR antagonists.

In this study, we aimed to develop a novel class of PXR antagonists and a PXR PROTAC degrader to explore their potential effects on PXR-mediated regulation in hepatocyte models. We discovered **11** (MI891) as a small-molecule PXR antagonist and compound **36** (MI1013) as a PXR degrader, composed of SPA70 PXR antagonist and pomalidomide, a CRBN ligand.

For the first time, we conducted a detailed investigation into the pharmacological inhibition of PXR in hepatocyte models. We compare gene expression profiles in differentiated HepaRG cells employing extensive RT-qPCR analyses and a proteomic investigation using the DigiWest system. This approach allowed us to examine hepatic gene regulation and estimate signaling pathway activation in cells treated with the PXR inverse agonist/antagonist MI891 or the MI1013 PROTAC degrader, in contrast to PXR agonists such as rifampicin. We demonstrate that these novel tools effectively modulate PXR function through antagonism or degradation and downregulate key PXR target genes in the absence or presence of rifampicin. Additionally, we show that these compounds suppress critical genes involved in lipid (e.g., *CYP4A11*), glucose (*PCK1*), bile acid (*CYP7A1* and *CYP7B1*), and cholesterol (*ABCG5/8*, *HMGCS2*) homeostasis, as well as hepatocyte proliferation (e.g., MKI67). These findings extend our understanding of PXR antagonism and PXR degradation in the regulation of endobiotic metabolism.

## Results

### Design and Discovery of A Novel Class of PXR Antagonists

#### Chemical Synthesis of the PXR Antagonist MI891

We harnessed the synthetic approach from our previous study^25^ and applied it to prepare a smaller library of compounds to further explore the structure–activity relationship (SAR). The newly prepared compounds are derived from our published compound **1**, for which we suggested to possess PXR inhibitory activity, and share the same substitution pattern of the phenyl ring or with minor modification, specifically para-chloro and meta-carboxamide substituents, as compound **1**. We have explored the effects of methylene bridge modification (route I), phenyl ring (B) modification (route II and III), 5-membered linker ring (C) alternation (route IV), and mainly substitution of one or two hydrogens of the central core (A) with fluorine atom (route V) (Figure 2). Importantly, all these structural modifications have not been studied in connection with the amide moiety on the 3-Cl-phenyl D ring on PXR and CAR activity in our previous report.^25^

**Figure 2:**
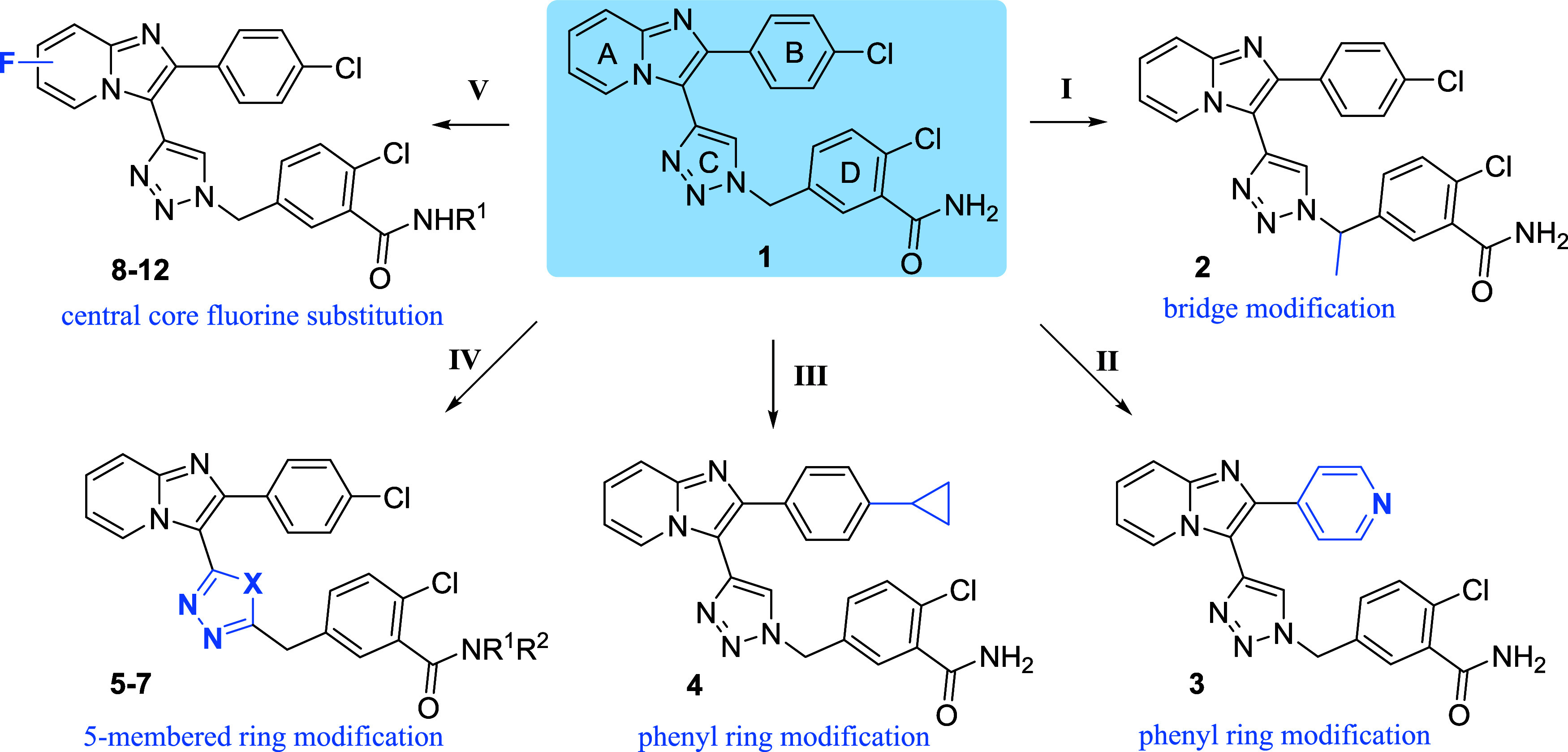
Synthetic strategies for the novel library.

Synthesis of compound **2** with a modified methylene bridge followed the original procedure, where the azide intermediate is clicked to an alkyne intermediate in a Cu-catalyzed click reaction, as depicted in Scheme 1. Similarly, the phenyl ring modification was achieved by exchanging the 2-bromo-1-(4-chlorophenyl)­ethan-1-one in the first cyclization step for 2-bromo-1-(pyridin-4-yl)­ethan-1-one under the same reaction conditions (compound **3**, Scheme 1). The cyclopropyl moiety (compound **4**) was installed after the cyclization step, where the 4-bromo substituted phenyl ring (**16**) was reacted with cyclopropyl boronic acid under the Pd catalysis as shown in Scheme 1.

**Scheme 1:**
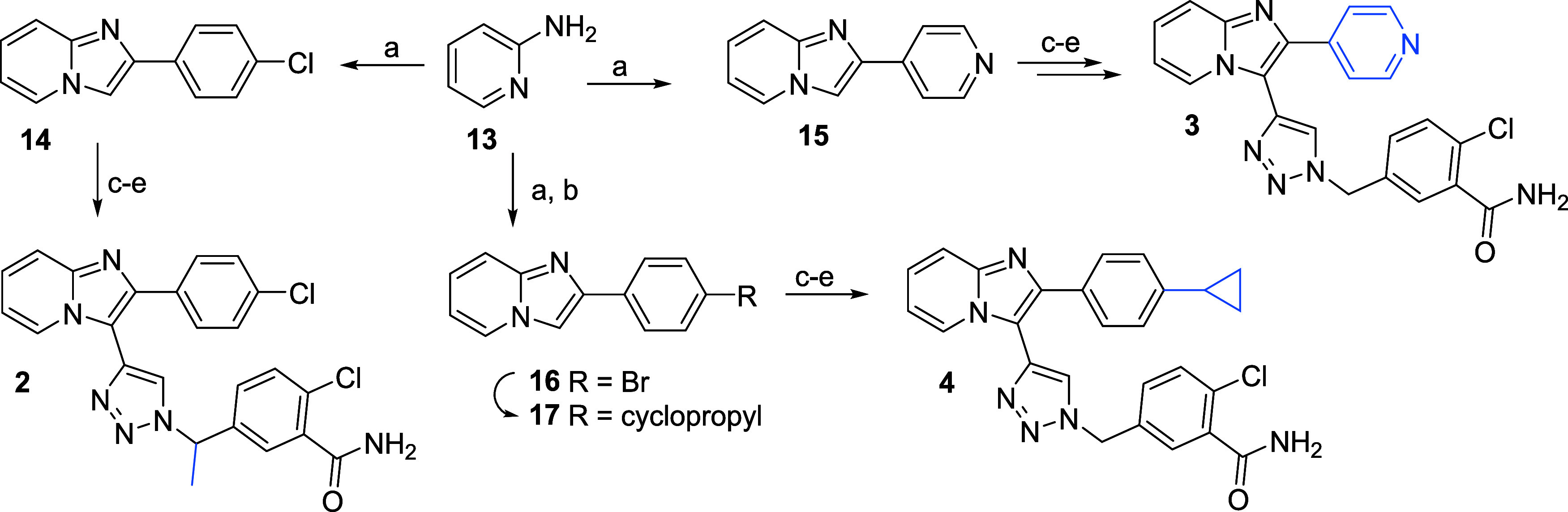
Chemical Synthesis of Phenyl Ring B Modifications and Linker Alternation^a^

a Reagents and conditions: (a) 2-bromo-1-(4-chlorophenyl)­ethan-1-one for **14**, 2-bromo-1-(pyridin-4-yl)­ethan-1-one for **15**, 2-bromo-1-(4-bromophenyl)­ethan-1-one for **16**, NaHCO_3_, EtOH, 70 °C, o/n; (b) cyclopropylboronic acid, K_3_PO_4_, Pd­(OAc)_2_, PCy_3_, H_2_O/CH_3_CN 1:1, reflux, 73%; (c) NIS, DCM, r.t.; (d) TMS-acetylene, CuI, TEA, Pd­(PPh_3_)_2_Cl_2_, DMF, 0 °C to r.t.; (e) 5-(azidomethyl)-2-chlorobenzamide, CuSO_4_.5H_2_O, KF, sodium ascorbate, THF/H_2_O (1:1), 0 °C to r.t., 1 h.

Synthesis of compound **1** analogs with varied 5-membered linker ring, where the original triazole moiety is exchanged for oxadiazole and thiadiazole, begins with 2-aminopyridine (**13**), which was reacted with ethyl 3-(4-chlorophenyl)-3-oxopropanoate in CH_3_CN in the presence of excess of CBr_4_ (Scheme 2). Reaction was stirred at 80 °C, providing the corresponding intermediate ethyl carboxylates **18**. Reaction with hydrazine under reflux conditions led to hydrazide intermediate **19**, which was further coupled with 2-(4-chloro-3-(methylcarbamoyl)­phenyl)­acetic acid providing intermediate **20** or 2-(4-chloro-3-(dimethylcarbamoyl)­phenyl)­acetic acid providing intermediate **21** (for the synthesis see Supporting Information). Dehydrating and closing step was carried out either using tosyl chloride for the oxadiazole derivative **5** or P_2_S_5_ for the sulfur-containing compounds **6** and **7** at elevated temperature. Undesirably, the cyclization step with P_2_S_5_ in the case of compound **6** led to thiocarboxamide with a closed oxadiazole ring, vice versa to compound **7**.

**Scheme 2:**
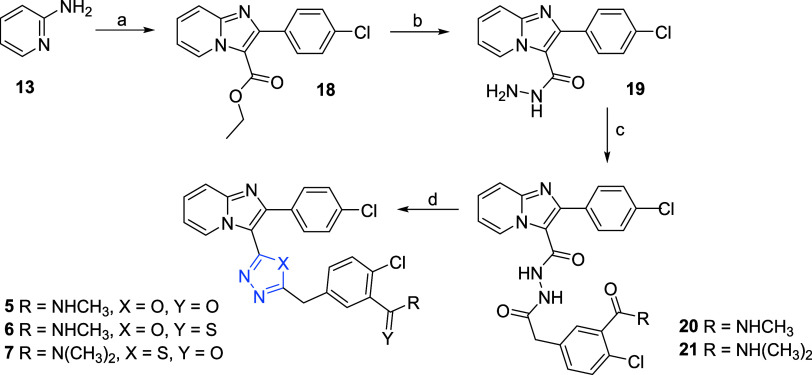
Chemical Synthesis of 5-Membered Ring C Derivatives^a^

a Reagents and conditions: (a) ethyl 3-(4-chlorophenyl)-3-oxopropanoate, CBr_4_, CH_3_CN, 80 °C, o/n, 82%; (b) N_2_H_4_·H_2_O, EtOH, reflux, o/n, 87%; (c) 2-(3-carbamoyl-4-chlorophenyl)­acetic acid, HATU, DIPEA, DMF, r.t., 86% for **20**, 83% for **21**; (d) tosyl chloride (1.5 equiv), TEA (3 equiv), DCM, 0 °C, 6% for **5**; P_2_S_5_, toluene, 100 °C, o/n, 36% for **6**; Lawesson’s reagent (3 equiv), toluene, 100 °C, o/n, 21% for **7**.

For the fluorinated series (Scheme 3), mono or di-fluorinated amino pyridine **22a**–**d** was reacted with 2-bromo-1-(4-chlorophenyl)­ethan-1-one at elevated temperature in EtOH in the presence of sodium hydrogen carbonate and fluorinated imidazopyridnes **23a**–**d** were isolated. Next, position 3 of intermediates **23a**–**d** was selectively iodinated with NIS in acetonitrile. During this fast reaction, a suspension was formed, which allowed fast work-up of the reaction by filtration of the products **24a**–**d**. Afterward, Sonogashira coupling reaction with TMS-acetylene and Pd­(PPh_3_)­Cl_2_ provided intermediates **25a**–**d** in moderate yields. Finally, Cu­(II)-catalyzed (Huisgen-Meldal) click reaction with 5-(azidomethyl)-2-chlorobenzamide or 5-(azidomethyl)-2-chloro-*N*-methylbenzamide yielded compounds **8**–**11** and compound **12**, respectively. Compound **9** has been investigated and already published independently.^26^

**Scheme 3:**
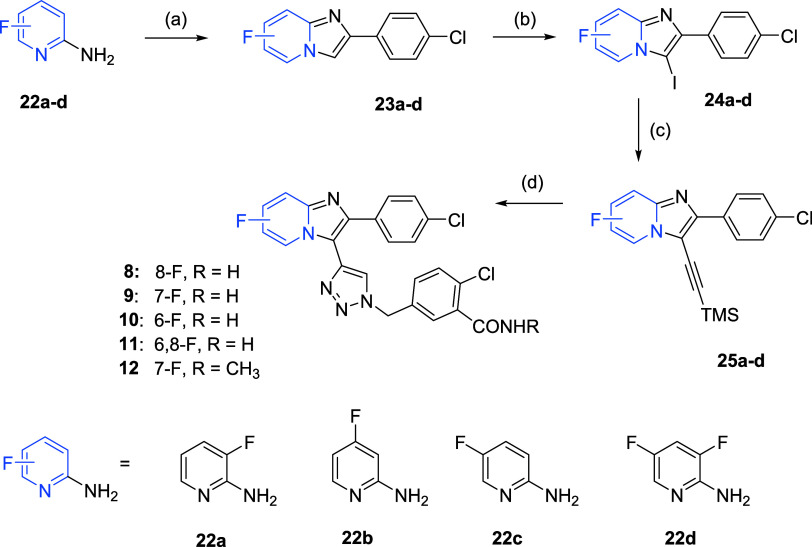
Chemical Synthesis of the Fluorinated Series^a^

a Reagents and conditions: (a) 2-bromo-1-(4-chlorophenyl)­ethan-1-one, NaHCO_3_, EtOH, 70 °C, o/n; (b) NIS, CH_3_CN, r.t.; (c) TMS-acetylene, CuI, TEA, Pd­(PPh_3_)_2_Cl_2_, DMF, 0 °C to r.t.; (d) 5-(azidomethyl)-2-chlorobenzamide or 5-(azidomethyl)-2-chloro-*N*-methylbenzamide, CuSO_4_·5H_2_O, KF, sodium ascorbate, THF/H_2_O (1:1), 0 °C to r.t., 1 h.

#### Compound **11** (MI891) Functions as a PXR Antagonist and Inverse Agonist

The synthesized library of imidazo­[1,2-*a*]­pyridine derivatives was evaluated for PXR antagonistic/inverse agonistic and agonistic activities in PXR-responsive luciferase reporter assays *in vitro*. In addition, we analyzed the activation of the human constitutive androstane receptor (CAR, NR1I3) in the CAR LBD assembly assays, CAR3 variant luciferase reporter assay, and TR-FRET CAR Coactivator assays. Table 1 summarizes the results, with rifampicin (100%) used as a reference ligand to measure the compounds’ PXR agonistic activity. We found that compounds with 1,2,3-triazol C ring heterocyclic linker and 3-amide or 3-*N*-methylamide substitution on the D ring exhibited PXR antagonistic (inhibition of rifampicin-mediated PXR activation) or inverse agonistic (effects on PXR basal activity) activities (Table 1). Specifically, 3-amide and 3-*N*-methylamide substitutions on ring D inhibited PXR activation, depending on the B ring substitutions (compounds **3** and **4**) or C ring heterocycle linkers (compounds **2**, **5**–**7**). Interestingly, the *N*-dimethylamide derivative (compound **41**, published in ref 25) did not inhibit PXR activation and exerted mild PXR activation (Table 1). A ring substitution with fluorine in position 6 or 8 did not affect PXR antagonistic activities but stabilized compounds against A ring metabolic hydroxylation (Table S1).^25^ Importantly, both double 6,8-difluorinated compounds on ring A (compounds **11** (MI891) and **12**) lost activity in CAR activation but displayed potent PXR antagonistic and inverse agonistic activities (Table 1). Other compounds activated CAR, except compound **3**, which has a pyridine B ring. Compound **7** acts as a partial PXR agonist, as it activates PXR while concurrently suppressing rifampicin-induced activation of PXR. We did not identify any neutral PXR antagonists among the compounds lacking inverse agonistic activity. We can summarize that fluorinated derivatives **10**–**12** exhibit significant PXR antagonistic activities but do not substantially activate CAR in any assay used (Table 1).

**Table 1:** Interactions of Newly Synthesized Compounds with PXR and CAR and Its CAR3 Variant in Luciferase Gene Reporter Cellular Assays and TR-FRET CAR Coactivation Assay

substitutions					PXR antagonism	PXR inverse agonism	PXR agonism	CAR agonistic activation		
compound	A ring substitution	B ring substitution	C ring (heterocycle linker)	D ring substitution	PXR inhibition (IC_50_, μM)^c^	decrease of basal activity in %^d^	PXR (% activity of Rif 10 μM)^e^	CAR TR-FRET (EC_50_, μM)	CAR AA (EC_50_, μM)	CAR3 (% activity of CITCO 1 μM)
**1** ^a^		4-Cl-phenyl	1,2,3-triazole	3-amide 4-Cl	1.10 ± 0.2	–73.10 ± 3.62		0.61 ± 0.1^a^	1.22 ± 0.3^a^	68 ± 4^a^
**2**		4-Cl-phenyl	1,2,3-triazol-1-y ethyl	3-amide 4-Cl	25.41 ± 3.5		47.3 ± 13	>5	5.3 ± 0.3	54 ± 7
**3**		pyridin-4-yl	1,2,3-triazol	3-amide 4-Cl	>100		7.59 ± 1.99	nb	na	na
**4**		4-cyclopropyl phenyl	1,2,3-triazole	3-amide 4-Cl	5.76 ± 2.6	–33.50 ± 0.50		nb	>20	11.2 ± 2
**5**		4-Cl-phenyl	1,3,4-oxadiazol	3-*N*-methylamide 4-Cl	>100		16.00 ± 5.10	0.401	1.02 ± 0.2	91 ± 7
**6**		4-Cl-phenyl	1,3,4-oxadiazol	3-*N*-methylthioamide 4-Cl	>100		19.33 ± 18.20	0.162	0.99 ± 0.15	176 ± 9
**7**		4-Cl-phenyl	1,3,4-thiadiazol	3-*N*-dimethyl amide 4-Cl	4.94 ± 0.9		8.67 ± 11.60	0.01 ± 0.2	0.50 ± 0.01	168 ± 10
**8**	8-F	4-Cl-phenyl	1,2,3-triazole	3-amide 4-Cl	12.45 ± 6.0	–31.27 ± 0.10		nb	4.14 ± 0.2	21 ± 4
**9** (MI-883)^b^	7-F	4-Cl-phenyl	1,2,3-triazole	3-amide 4-Cl	3.60 ± 0.4	–72.03 ± 0.3		0.073 ± 0.02	0.38 ± 0.06	82
**10**	6-F	4-Cl-phenyl	1,2,3-triazole	3-amide 4-Cl	6.46 ± 0.6	–13.50 ± 5.50		nb	>10	11 ± 3
**11** (MI891)	6,8-diF	4-Cl-phenyl	1,2,3-triazole	3-amide 4-Cl	3.76 ± 0.4	–68.84 ± 5.53		nb	>10	3 ± 1
**12**	6,8-diF	4-Cl-phenyl	1,2,3-triazole	3-*N*-methylamide 4-Cl	8.04 ± 2.3	–44.47 ± 2.04		nb	na	na
**40** ^a^		4-Cl-phenyl	1,2,3-triazole	3-*N*-methylamide 4-Cl	6.56 ± 2.5	–80.30 ± 3.30		1.61 ± 0.1^a^	1.53 ± 0.3^a^	63 ± 4^a^
**41** ^a^		4-Cl-phenyl	1,2,3-triazole	3-*N*-dimethyl amide 4-Cl	>500		7.00 ± 6.48	1.07 ± 0.1^a^	1.09 ± 0.1^a^	91 ± 8^a^
**43** ^a^		4-Cl-phenyl	1,2,3-triazole	3-ethan-1-one 4-Cl	>50		30.0 ± 20	0.001^a^	tox	333 ± 30^a^
SPA70					4.11 ± 2.80	–61.07 ± 3.00				
rifampicin							100			
CITCO								0.012 ± 0.004*	0.69 ± 0.04*	100

a Published in ref 25.

b Published in ref 26. nb, no binding; na, no activity; tox, cytotoxicity in HepG2 cells.

c Antagonist mode with 10 μM rifampicin.

d Inverse agonism, DMSO (0.1%) is considered to represent 100% basal activity of PXR expression, tested at 10 μM.

e Agonistic mode for PXR activation relative to 10 μM rifampicin (Rif, 100%), tested at 10 μM. Values represent means ± SD from *n* = 3 (biological replicates).

Next, we analyzed the microsomal stability of A-ring fluorinated compounds **8**, **10**, and **11** (MI891) in mouse and human microsomes. We found that compound **11**, which has two fluorine atoms on ring A, displayed substantial stability in these experiments. In contrast, compounds **8** and **10**, with only one fluorine on ring A, demonstrated less microsomal stability (Table S1).

MI891 (compound **11**) (Figure 3A) is a relatively potent PXR antagonist in the cellular luciferase reporter antagonist assay, with negligible CAR activation (Table 1). In detailed luciferase reporter gene assays with the PXR-responsive construct derived from the *CYP3A4* gene promoter, we observed that MI891 concentration-dependently abrogates PXR activation by rifampicin (Figure 3B) and displays antagonistic activity with an IC_50_ of 378 nM for rifampicin (1 μM)-activated PXR. In the antagonist mode, MI891 showed a potent reduction of rifampicin induced PXR activation, demonstrating effectiveness comparable to SPA70 at 10 μM (IC_50_ 3.8 vs 4.1 μM; Table 1). Next, we demonstrate that MI891 exhibits inverse agonistic activity with an IC_50_ of 6.1 μM and maximal inhibition of 68.8 ± 5.5% at 10 μM (Figure 3B, Table 1). Interestingly, MI891 is a less potent PXR inverse agonist compared to SPA70 in these experiments (Figure S1A,B). MI891 also suppressed both rifampicin-induced and basal (constitutive) *CYP3A4* mRNA expression in HepaRG cells in a concentration-dependent manner (Figure 3C,D). A similar phenomenon in *CYP3A4* mRNA suppression was observed for SPA70, a known PXR antagonist (Figure S1C).

**Figure 3:**
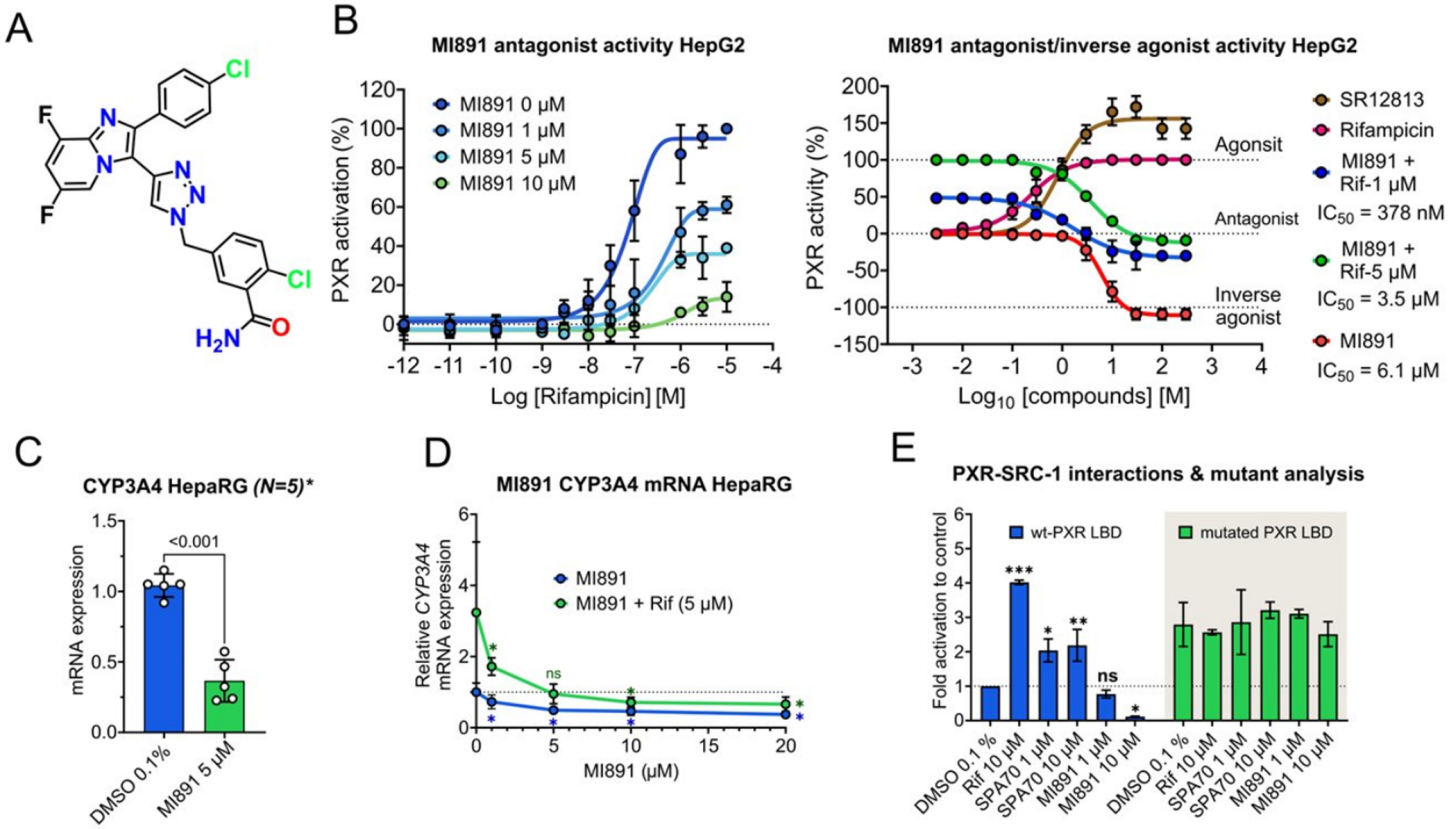
Discovery and characterization of MI891, a novel PXR antagonist. (A) Chemical structure of MI891. (B) Dose–response curves showing the antagonistic effect of MI891 in HepG2 cells transfected with the PXR-responsive *CYP3A4* gene promoter luciferase reporter construct under increasing concentrations of rifampicin (left panel). Dose–response curves of MI891’s antagonistic effect at constant rifampicin concentrations (1 or 5 μM) (antagonistic mode) and with MI891 alone (inverse agonist mode) (right panel). SPA70 represents 100% inhibition to basal activity, and rifampicin represents 100% PXR activity at 10 μM, as shown in the figure. Vehicle (DMSO, 0.1%) is set as 0% PXR activity. Curve fitting (nonlinear regression) was performed using log­(inhibitor) vs response using GraphPad Prism software. (C–D) *CYP3A4* mRNA expression levels in HepaRG cells treated with MI891 alone or in combination with rifampicin (5 μM) for 48 h. Data are normalized to *HPRT1* mRNA expression and expressed as fold change relative to vehicle control. Statistical significance between groups was assessed by one-way ANOVA followed by Dunnett’s multiple comparisons test (DMSO to other molecules, *p < 0.05; ns, not significant). (E) Mammalian two-hybrid assay with wild-type (wt-PXR LBD) or triple mutant (S208W/S247W/C284W) obstructed PXR-LBD and the coactivator peptide SRC-1-VP16 in HepG2 cells. Data are presented as fold activation relative to the vehicle-treated and GAL-4 wt-PXR-LBD/SRC-1-VP16-transfected sample (set to 1). Statistical significance between groups was assessed by one-way ANOVA followed by Dunnett’s multiple comparisons test (DMSO to other molecules, *p < 0.05, **p < 0.01, or ***p < 0.001; ns, not significant). All data are the mean ± SD, *n* = 3.

To confirm the interaction of MI891 with the PXR LBD, we performed a two-hybrid luciferase assay with wild-type and triple mutant (S208W/S247W/C284W) PXR LBD expression constructs (Figure 3E). Interestingly, while SPA70 activated the wild-type (wt) PXR LBD construct, MI891 at 10 μM disrupted the wt-PXR-LBD/SRC1-VP16 interaction, suggesting that MI891 binding abrogates PXR LBD coactivation with steroid receptor coactivator 1 (SRC1) (Figure 3E). Neither MI891 nor SPA70 activated the obstructed mutated PXR LBD. Therefore, MI891 acts as a potent PXR antagonist and inverse agonist in the PXR LBD cavity.

#### Structure–Activity Relationship (SAR) Studies of MI891’s Antagonistic Properties

To evaluate the binding affinity of MI891 for PXR, we performed microscale thermophoresis (MST) measurements to determine the dissociation constant (*K*_D_) of MI891 with the PXR-eGFP chimeric protein. MI891 bound to PXR with a *K*_D_ value of 1.7 μM (Figures 4A and S1D).

**Figure 4:**
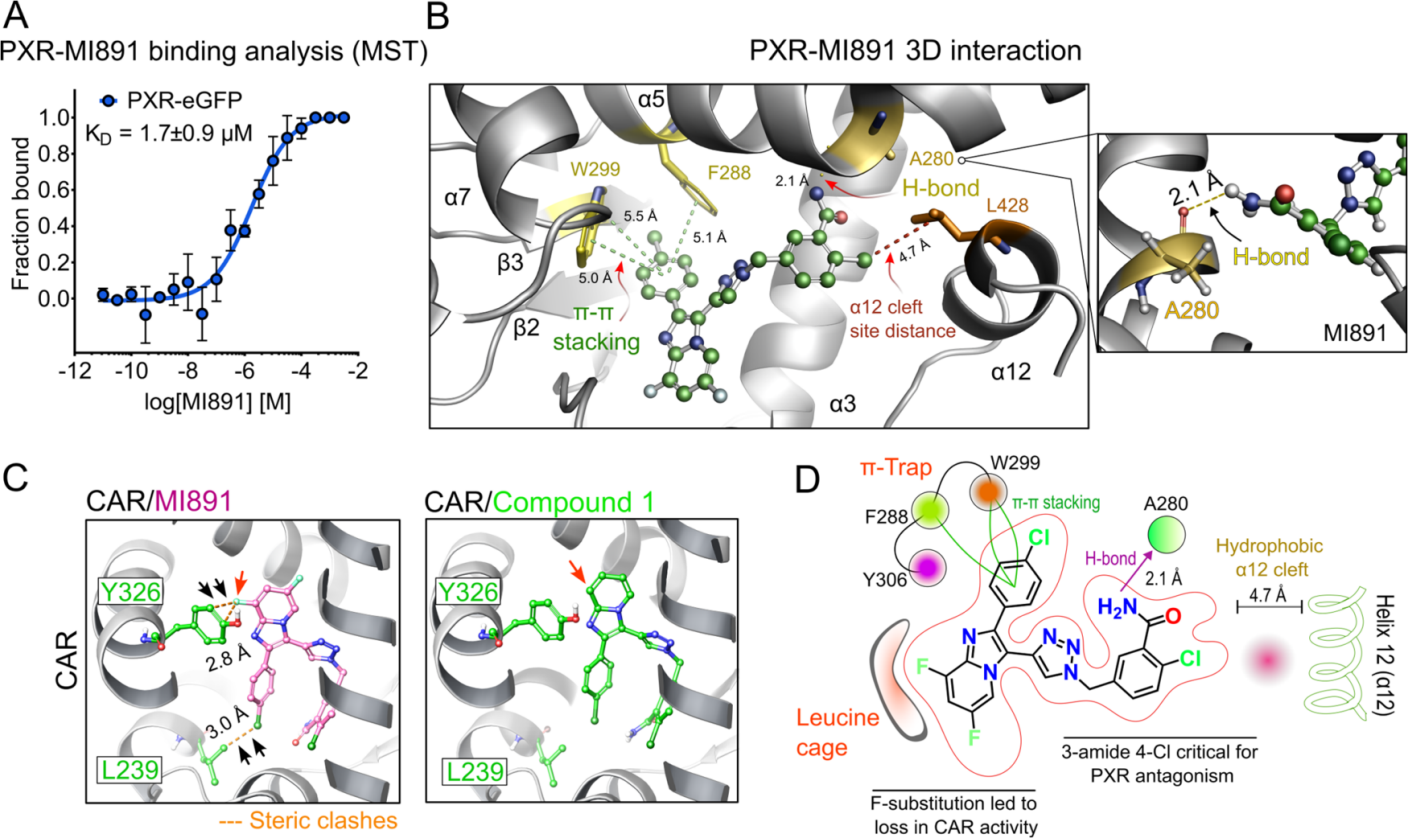
Binding Affinity and Molecular Interactions of MI891 with PXR. (A) The binding affinity of MI891 to PXR-eGFP was measured by microscale thermophoresis (MST) assay (*K*_D_ value calculated by MO. affinity analysis software using *K*_D_ fit model). Curve fitting (nonlinear regression) was performed using log­(inhibitor) vs response with a variable slope (four parameters). (B) Molecular interactions of MI891 with PXR. MI891-bound PXR model (PDB: 8SVT). Three-dimensional (3D) interaction model of MI891 with PXR at the ligand-binding site. Yellow dashed lines represent hydrogen bonds, and light green lines represent pi-pi stacking. Measured distances are highlighted. Red dashed lines represent the α12–ligand distance. The right-side box highlights the view of the hydrogen bond interaction between MI819 and PXR. (C) CAR-MI891 or compound **1** ligand docking (PDB: 1XVP): MI891′s (pink) chlorine (Cl) and fluorine (F) sites form steric clashes (orange dashed lines) with the CAR LBD, which are not observed with compound 1. (D) Structure–activity relationship (SAR) analysis of MI891 showing the critical sites for PXR interactions and regions for modulating the CAR activity.

These data were in excellent agreement with docking results, which suggested a binding free energy of −10.7 kcal/mol. MI891 proposed binding mode shows hydrophobic contacts and pi-mediated interactions between the chlorine-phenyl group with the “π-trap” pocket, consisting by the F288 (α5), W299 (β3), and Y306 (β4) residues, as well as a “leucine cage” composed of the L206, L209, L239, and L240 residues (Figures 4B,D and S1E–F). In addition, MI891’s amide substituent stabilized a relevant H-bond interaction with the backbone from A280 (α5), while leaving the hydrophobic cleft formed by residues M425, L428, and F429 in the α12 helix unoccupied (Figure 4B,D). This missing interaction with α12 helix has been described as critical for other PXR antagonists (e.g., SJPYT-331), ultimately leading to destabilization of the AF-2 domain necessary for coactivator binding.^6,12^ However, agonists resides in close proximity to L428 of the α12 helix in the PXR-LBD to stabilize the AF2 domain (Figure S1F). Since PXR-LBD is highly flexible, and therefore a more precise description of this behavior would require a dynamic structural model with solvent.^27^

In the next docking experiments, we demonstrate the role of the A ring substitution on CAR activity and the MI891′s pose in the CAR LBD (Figure 4C,D). We demonstrate that fluorine in position 6 clashes with Y326 of the CAR LBD, thus resulting in low CAR activation by compounds **10**, **11**, and **12** (Table 1). Interestingly, the presence of two fluorines in positions 6 and 8 almost abrogates the CAR activities of compounds 11 and 12 (Table 1) and stabilizes compound **11** (MI891) in microsomal stability assays (Table S1).

We can conclude that 6,8-fluorines are critical for the elimination of CAR activation of compound **1**, but 3-amide 4-Cl substitution of ring D is critical for PXR antagonistic/inverse agonistic activity of compound **11** (MI891) (Figure 4D).

#### Selectivity Profile of MI891 to PXR Orthologues and Other Nuclear Receptors

Sister nuclear receptors PXR and CAR together with the Vitamin D receptor (VDR), constitute the NR1I subfamily of nuclear receptors. MI891 showed no binding activity and an EC_50_ greater than 10 μM, confirming its minor effect on wild-type CAR (Table 1). In further dose–response experiments, we thoroughly assessed MI891’s ability to activate CAR using luciferase reporter gene assays with human wild-type CAR (CAR^wt^) and the CAR variant (CAR3). MI891 required significantly higher concentrations to activate CAR compared to CITCO, specifically having an estimated EC_50_ of 137 μM, which is 55 times higher than of CITCO. While MI891 activated CAR at high concentrations, we observed no significant activation of either CAR^wt^ or CAR3 variant at concentrations up to 10 μM (Figure 5A). In the CAR LBD assembly assay, we noted a lack of significant (*p* value 0.07) activation of CAR LBD assembly at 10 μM concentration (Figure 5A).

**Figure 5:**
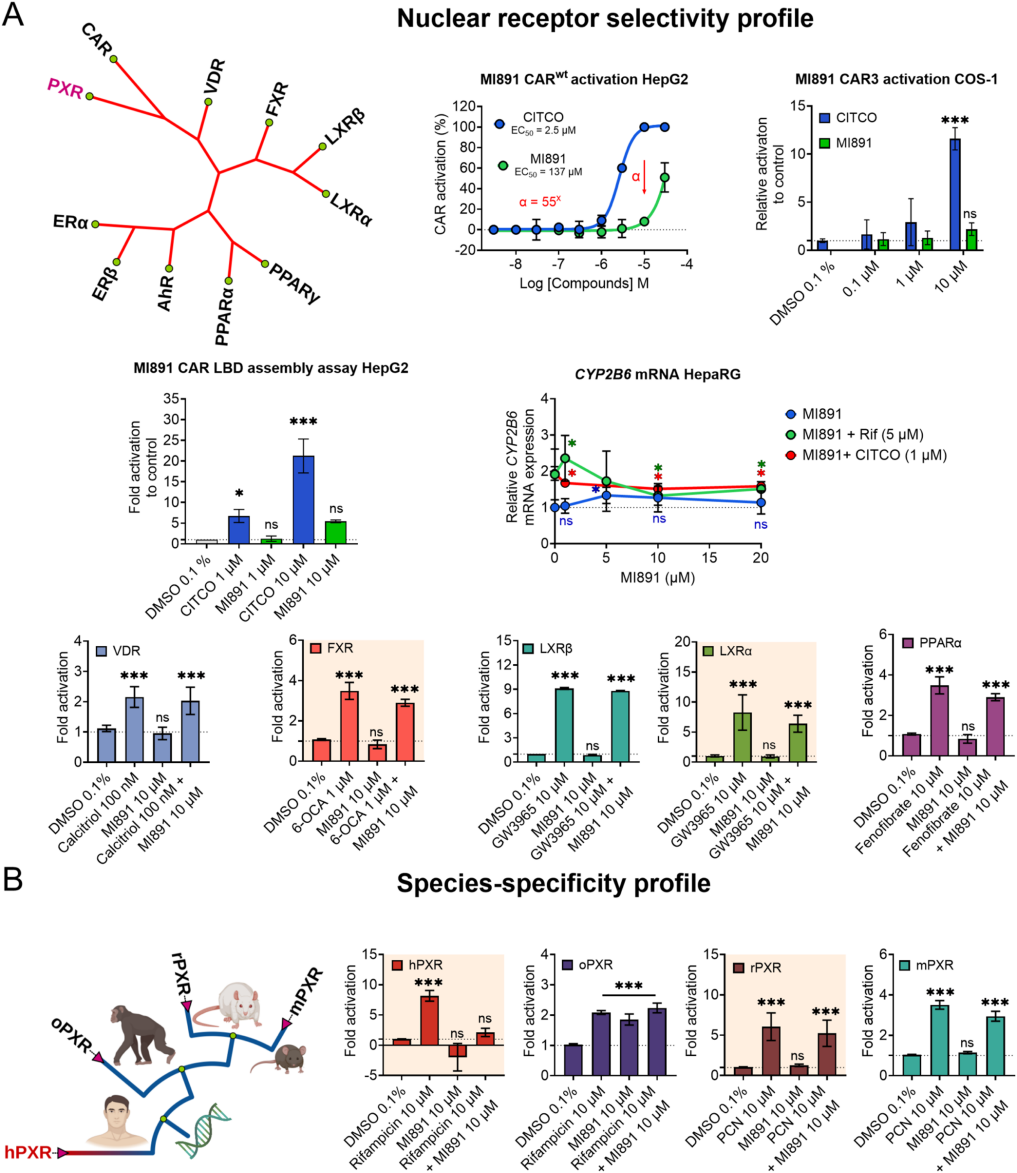
MI891 exhibits selectivity and species-selectivity for PXR. (A) Selectivity of MI891 for PXR and related nuclear receptors. The phylogenetic tree (left), luciferase reporter assays (CAR^wt^, CAR3 and CAR LBD assembly) data in HepG2 and COS-1 cells, and *CYP2B6* mRNA expression data in HepaRG cells treated with MI891 alone or in combination with rifampicin (5 μM) or CITCO (1 μM) for 48 h. Data are normalized to *HPRT1* mRNA and expressed as fold change relative to vehicle control (top center). Interaction of MI891 with vitamin D receptor (VDR), farnesoid X receptor (FXR), liver X receptors (LXRβ and LXRα), and peroxisome proliferator-activated receptor α (PPARα) in luciferase reporter assays in both agonistic and antagonistic setups using prototype ligands, calcitriol, obeticholic acid (6-OCA), GW3965, and fenofibrate (bottom). (B) Phylogenetic tree of PXR orthologs (left) and their responses to MI891 and reference ligands in luciferase reporter assays (right). Rifampicin and PCN were used as prototypical inducers for human/monkey and rat/mouse PXR, respectively. Data are presented as mean ± SD, *n* = 3. *p < 0.05; ***p < 0.001 (ANOVA with Dunnett test; MI891 and reference ligands vs DMSO 0.1%).

In HepaRG cells, MI891 slightly induced *CYP2B6* mRNA, a key target gene of CAR, and enhanced rifampicin-mediated induction of *CYP2B6* mRNA at the concentration of 1 μM. However, no significant activation was observed at higher concentrations (Figure 5A). Notably, MI891 did not exhibit an additive effect with CITCO on *CYP2B6* mRNA expression (Figure 4A). Overall, MI891 functions as an antagonist of PXR at micromolar concentrations, but it exhibits limited activity on CAR at high concentrations (Figure 5A).

Next, we observed no significant agonistic or antagonistic activities of MI891 on human VDR, FXR, LXRβ, LXRα, and PPARα receptors (Figure 5A). Furthermore, no agonistic effects of MI891 were observed in PPARγ, AhR, or ERα/β receptor assays (Figure S1G). MI891 exhibited selective antagonism and inverse agonism on human hPXR but showed no activities on rodent PXRs (Figure 5B). In contrast, rhesus monkey PXR (oPXR) displayed full agonism with MI891, but not antagonism.

#### *In-Silico* Prediction of Toxicity, ADME, and Off-Target Kinase Profile of MI891

MI891 demonstrated no effects on viability in various cell lines (up to 30 μM), including COS-1, HepG2, and HK-2 (Figure S2A). The *in silico* analysis of its physicochemical and ADME properties suggests that MI891 has the potential to be further developed as a lead compound (Figure S2B).^28^ Modeling for membrane permeability indicates that MI891 can passively transport across the plasma membrane, facilitating its access to PXR-containing structures in cellular models (Figure S3A).^29^

To evaluate the selectivity of MI891, an off-target profiling study was performed using a panel of 58 kinases. MI891 did not exhibit significant inhibitory activity against any of the kinases tested, although a moderate inhibition of 53% was observed for PIM-1 kinase at 1 μM (Table S2).

In conclusion, MI891 is identified as a highly selective PXR antagonist with significant efficacy in the micromolar range and minimal activation of CAR. Our data demonstrate that MI891 effectively inhibits rifampicin-induced PXR activation and exhibits inverse agonistic properties, with a notable IC_50_ of 378 nM for PXR antagonism and 6.1 μM for inverse agonism. Structural and binding studies reveal specific interactions between MI891 and the PXR ligand-binding domain, including hydrogen bonding and π–π interactions, which contribute to its selectivity. Importantly, MI891 shows limited activity on other nuclear receptors and kinases.

### Design, Synthesis, and Characterization of MI1013 as a PXR Degrader

In the next phase of this research, we focused on designing and synthesizing a bifunctional PROTAC molecule using SPA70 as a ligand for the PXR LBD. This type of PROTAC proved to be more feasible than the PROTAC with the MI891 ligand.

#### Structure-Based MI1013 Design and Selection: *In Silico*

We developed a structure-based method to design and select PXR-targeting PROTACs, which are dual-functional molecules that induce the degradation of target proteins by linking them to E3 ubiquitin ligases, thereby marking them for proteasomal destruction. Using the crystal structure of PXR LBD (PDB ID: 5 × 0R), we designed a MI1013 degrader (Figure 6A,D). Docking studies with SPA70 showed a binding free energy of −9.446 kcal/mol, with key interactions of SPA70 at F429 and H407, and the protruding dimethyl–ethyl-phenyl part of the molecule from the PXR LBD (Figures 6A and S4A). This moiety has been considered as a linker access site. Limited PXR-interacting E3 ligase ligands (UBR5, RBCK1, FBXO44, TRIM21) constrained the PROTAC design (Figure 6B). Therefore, we selected the CRBN ligase ligand pomalidomide based on the gene expression profile of CRBN compared to other known E3 ligases, such as VHL (Figure S4B).^30^ Additionally, CRBN is abundantly expressed in various tissues compared to other E3 ligases and has well-characterized, high-affinity ligands, such as pomalidomide, which binds to CRBN with an affinity of 157 nM.^31^ In addition, a thalidomide-based PROTAC has already been demonstrated as an efficient tool in PXR degradation in tumor cells.^21^

**Figure 6:**
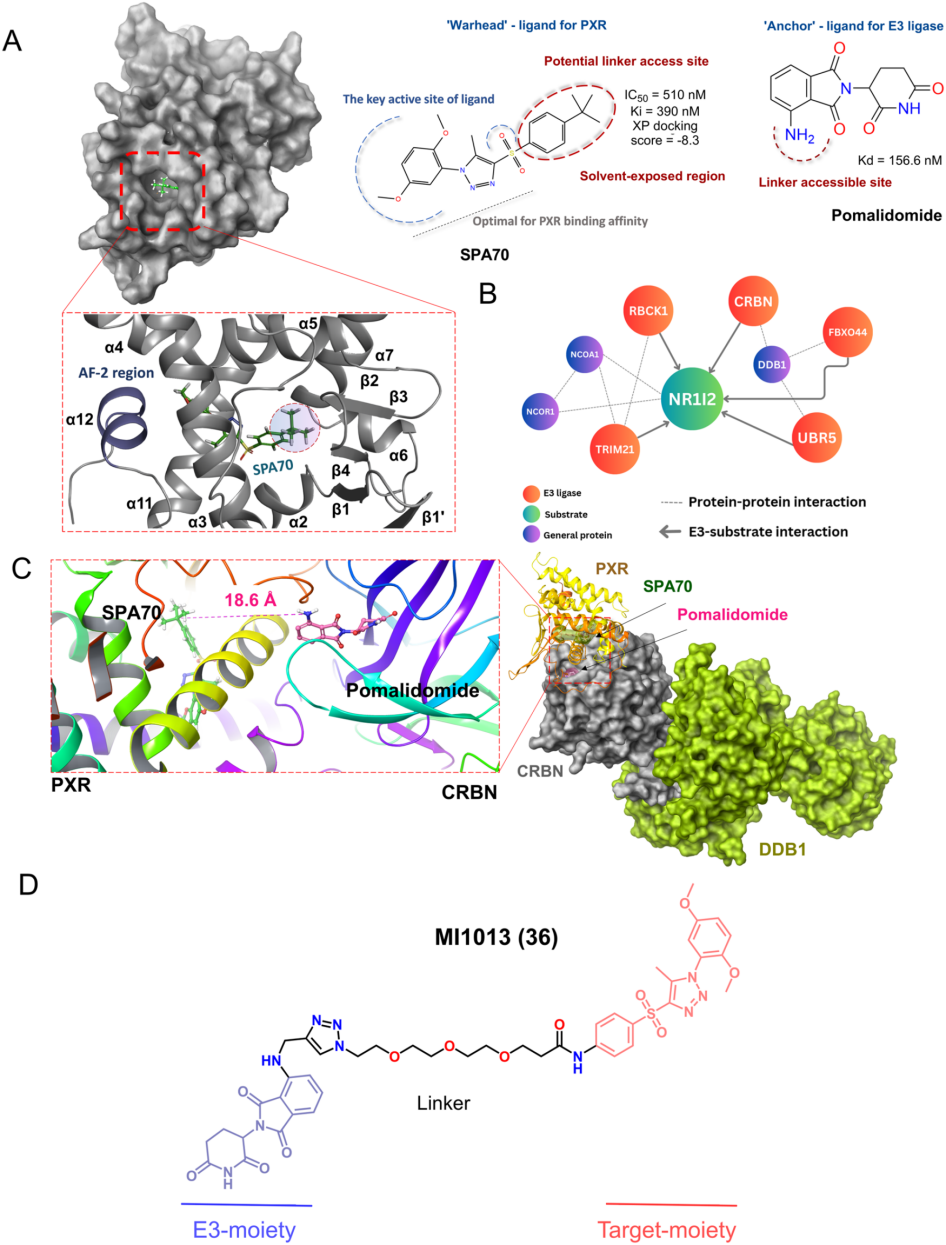
*In silico* design of MI1013 degrader. (A) PXR LBD with docked SPA70 pose (PDB ID: 5 × 0R). (B) E3 ligases reported for interaction with PXR. NR1I2, nuclear receptor subfamily 1 group I member 2 (PXR); CRBN, cereblon; DDB1, DNA damage-binding protein 1; FBXO44, F-box only protein 44; UBR5, Ubiquitin protein ligase E3 component n-recognin 5; RBCK1, Ring-B-box-coiled-coil protein interacting with protein kinase C-1; TRIM21, tripartite motif containing 21; NCOA1, nuclear receptor coactivator 1; NCOR1, nuclear receptor corepressor 1. (C) Protein–protein molecular stimulation of PXR and CRBN, with the distance measured between anchored ligands. (D) Designed the MI1013 (compound **36**) structure with PEG linker.

First, we used the degronopedia web server to identify the degron motif KK-LQLHEEE in the PXR protein (Figure S4C).^32^ Degrons are short linear motifs recognized by E3 ubiquitin ligases, tagging proteins for degradation. Identifying the degron is crucial, as it ensures that the designed PROTAC can effectively recruit the E3 ligase to the target protein, facilitating its ubiquitination and subsequent degradation. The identified degron in PXR had low disorder scores, indicating its relevance (Figure S4C).

Next, we aimed to find the CRBN binding site on PXR using HADDOCK (High Ambiguity Driven protein–protein DOCKing), an information-driven flexible docking approach.^33^ The docking simulations predicted two potential interaction sites between E3 ligases and PXR (Figure S5). Structural models of PXR/CRBN complexes showed strong binding affinity between PXR and CRBN (HADDOCK score: −194.1 ± 1.9), driven by hydrogen bonding and van der Waals and hydrophobic interactions (Table S3; Figure S6). The predicted binding affinity for PXR/CRBN is Δ*G* = −14.8 kcal/mol (Table S4). Further analyses optimized the linker length to 18.6 Å, facilitating E3 ligase proximity to PXR (Figure 6C) (see Protein–Protein Docking). Based on this distance, a PROTAC molecule MI1013, was designed by connecting SPA70 and pomalidomide with a polyethylene glycol (PEG) linker (Figure 6D).

Using PRosettaC, the PROTAC molecule MI1013 was modeled to generate near-native ternary complex models.^34^ The PRosettaC run produced 21 top models, which were clustered based on structural similarity. The first cluster showed a promising outlook for further use. After modeling, the MI1013-bound PXR:CRBN complex revealed mainly hydrophobic interactions, with a key hydrogen bond between pomalidomide and Trp382 and His380 in CRBN (Figure S7).^35^

#### Chemical Synthesis of the PXR PROTAC Molecule

The synthesis of MI1013 (compound **36**) involved the ligation of two components prepared separately, namely the preparation of E3 ligand based on a pomalidomide structure **30** and PXR inhibitor **34b** possessing a PEG linker. First, azide intermediate **27** was prepared via diazotation reaction of 2,5-dimethoxyaniline **26**.

Next, the coupling partner compound **30** was synthesized in two steps from 4-fluoroisobenzofuran-1,3-dione (**28)**, which upon reaction with HCl salt of 3-aminopiperidine-2,6-dione under reflux conditions in acetic acid provided the intermediate **29**, which was subsequently alkylated with propargyl bromide, yielding compound **30** in lower yield. Intermediate **32**, *N*-(4-((2-oxopropyl)­sulfonyl)­phenyl)­acetamide for the cyclization reaction was prepared in two steps from 4-acetamidobenzenesulfonyl chloride (**31**), This compound was in initially converted to its sodium salt with Na_2_SO_3_ and NaHCO_3_ and then reacted with chloroacetone in the presence of catalytic amount of KI overnight. Subsequently, intermediate **32** was cyclized in the Dimroth cyclization reaction with separately prepared azide 27 at an elevated temperature of 60 °C overnight, providing intermediate **33**. The acetyl group was also removed during this step.

In the following reaction step, the PEG linker featuring two functional groups (azide and carboxylic acid) was attached to an amino group of compound 33 via amide bond formation using HATU as a coupling reagent, leading to intermediate **34b**. Finally, Cu catalyzed click reaction (CuAAC) took place in the presence of copper sulfate as the Cu source and sodium ascorbate as a reducing agent, yielding the final PROTAC compound MI1013 (**36**) (Scheme 4 and Figure 6D).

**Scheme 4:**
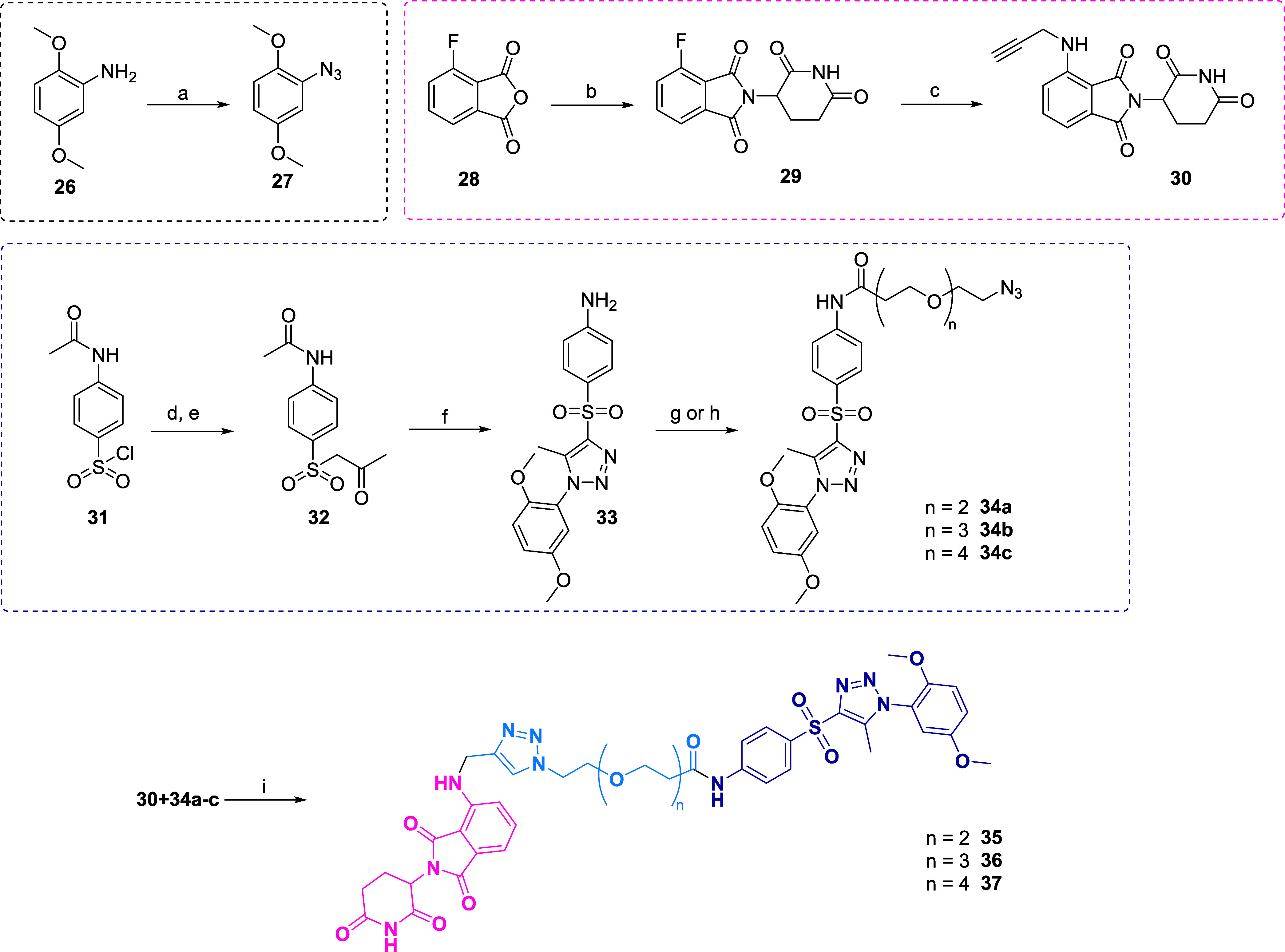
Synthesis of PROTACs **35**, **36** (MI1013), and **37** ^a^

a Reagents and conditions: (a) NaNO_2_, HCl, H_2_O, 0 °C, hexane, NaN_3_, 32%; (b) 3-aminopiperidine-2,6-dione.HCl, AcOH, NaOAc, reflux, o/n, 97%; (c) propargyl bromide, DIPEA, DMSO, 90 °C, 1 h, 31%; (d) Na_2_SO_3_, NaHCO_3_, H_2_O, 80 °C, EtOH, quant.; (e) chloroacetone, CH_3_CN, KI, o/n, 76%; (f) **27**, MeONa, MeOH, 60 °C, o/n, 16%; (g) N_3_–PEG-COOH, HATU, DMF, r.t., 2 h, 20% for **34b**; (h) SOCl_2_, DCM, rt, 2h, quant., then NMP, rt, o/n, for **34a** and **34c**; (i) **30** and **34a**–**c**, CuSO_4_.5H_2_O, sodium ascorbate, THF/H_2_O 1:1, 3 h, 70% for **35**, 74% for **36**, 74% for **37**.

In addition to this optimal PXR PROTAC, we designed two PROTAC molecules, MI1161 (**35**) and MI1160 (**37**), with shorter and longer linkers (Figure S8B). The preparation of shorter and longer analogues followed the aforementioned procedure with minor modifications. Azide-PEG2/4-carboxylic acid was treated with thionyl chloride in DCM at room temperature for 2 h, volatiles were evaporated, and the PEG2/4 acyl chlorides were reacted with SPA70 analogue **33** in NMP. Finally, Cu catalyzed click reaction with propargyl pomalidomide **30** provided the final PROTACs **35** and **37**, respectively.

Lastly, the methylated analogue of MI1013 (compound **36**) was also synthesized from *N*-methyl propargyl pomalidomide (**S15**).

#### Degradation of PXR by MI1013 PROTAC Molecule

The activity of the PROTAC degraders was primarily assessed using Western blotting, which measures relative protein levels. The cellular on-target effect of PROTACs on PXR expression and activity was tested in differentiated hepatic HepaRG cells, which naturally express high PXR activity and expression. MI1013 (**36**) PROTAC significantly reduced the PXR receptor after treatment with various concentrations, yielding a DC_50_ value of 89 nM and a *D*_max_ of 82% (Figure 7A,B). The compounds **37** with a longer linker also exhibit a mild effect on PXR degradation, but compound **35** with a shorter linker has no effect on PXR degradation (Figure 7A,B). A hallmark of PROTAC technology is the “hook effect”, where high concentrations of degraders result in reduced degradation due to saturation of binding and the formation of binary complexes instead of ternary complexes.^36^ This phenomenon was observed with MI1013 at 10 μM in HepaRG cells (Figure 7B). The proteasome inhibitor MG132 prevented the MI1013-induced degradation of PXR, confirming the involvement of the proteasome pathway. A time-course or kinetic experiment with HepaRG cells showed significant degradation of PXR after 12 h of treatment with MI1013 (**36**), but reversal of PXR expression after 72 h (Figure 7C). A more specific assessment of the MI1013 activity at 10 μM was conducted using the HiBiT luminescent assay, in which PXR degradation was measured based on the nanoluciferase activity of luciferase-tagged PXR (Figure S8C). We confirmed PXR degradation when we transfected cells with 0.3 ng of the PXR-HiBiT vector (under the strong CMV promoter), but not with a higher amount of the construct. This suggests a high expression and saturation of MI1013/CRBN in HepG2 cells when transfected with excess PXR-HiBiT.

**Figure 7:**
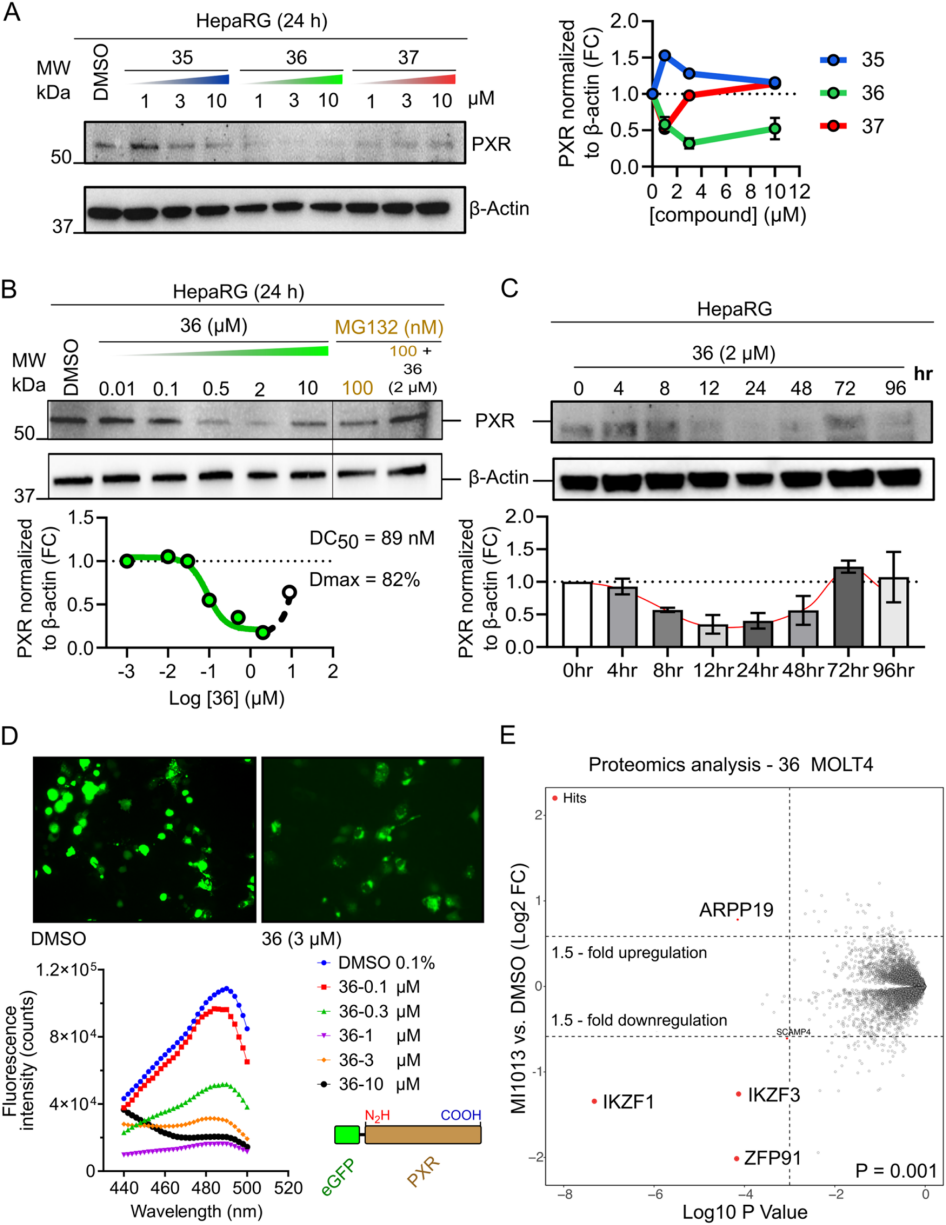
MI1013 induces degradation of PXR under different conditions. (A) Western blot image (left) and quantification (right) of PXR levels in HepaRG cells after 24 h of treatment with PXR–PROTAC molecules **35**, **36**, and **37**, normalized to β-actin (set as 1). (B) Western blot analysis of HepaRG cells treated with MI1013 (**36**) at various concentrations for 24 h. MG-132 at 100 nM was used as proteasome inhibitor. Curve fitting (nonlinear regression) was performed using log­(inhibitor) vs response-variable slope (four parameters). (C) Western blot analysis of HepaRG cells treated with 2 μM MI1013 (**36**) for various time points (kinetic). (D) Representative microscope image and fluorescence intensity quantification showing MI1013 (**36**)-mediated PXR-eGFP degradation in a concentration-dependent manner in COS-1 cells. (E) Off-target quantitative proteomics analysis in MOLT4 cells (a PXR-nonexpressing cell line). After MI1013 (**36**) treatment, off-target effects were evaluated. The volcano plot illustrates the downregulated proteins, showing a typical off-target profile for an IMiD-based degrader, with pomalidomide-downregulated proteins detected. The dashed line represents a statistically significant change in protein expression.

To further demonstrate the degradation of PXR by MI1013, the eGFP-tagged PXR system was analyzed using a fluorescent analyzer and microscopy in COS-1 cells. In the experiments, we observed dose-dependent degradation of eGFP-tagged PXR by MI1013, resulting in the loss of fluorescence (Figure 7D). In the next experiments, we analyzed the off-target effects of MI1013 PROTAC in MOLT4 (a human lymphoblast cell line) cells using global proteomics methods. The results indicate that MI1013 does not affect the expression of other proteins, including GSPT1,^20^ which remains undegraded after treatment with 2 μM of MI1013 (Figure 7E). The data showed a typical off-target profile for an IMiD-based degrader,^37^ confirming the specificity of MI1013. We observed degradation of blood cell Ikaros family zinc finger proteins 1 and 3 (IKZF1 and IKZF3), and ZFP91, a zinc finger protein related to atypical E3 ubiquitin ligase.^37^ These proteins are involved in immune response and T-cell proliferation but are unlikely to affect PXR protein expression in hepatocyte cells (Figure 7E). Pomalidomide was used as a positive control in these experiments to validate the degradation of known IMiD targets (Figure S9A).

In the next experiments, we confirmed that MI1013, but not MI1155 (S15), a PROTAC with methylated pomalidomide, selectively degrades PXR in HepaRG cells (Figure 8A,B). In addition, MI1013 does not degrade RXRα and GSPT1 ^20^ (Figure 8B). Importantly, neither precursor of MI1013 degrades PXR in Western blotting experiments (Figure 8C). However, some precursors appeared to activate PXR in luciferase reporter assays in transiently transfected HepG2 cells (Figure S9B–D).

**Figure 8:**
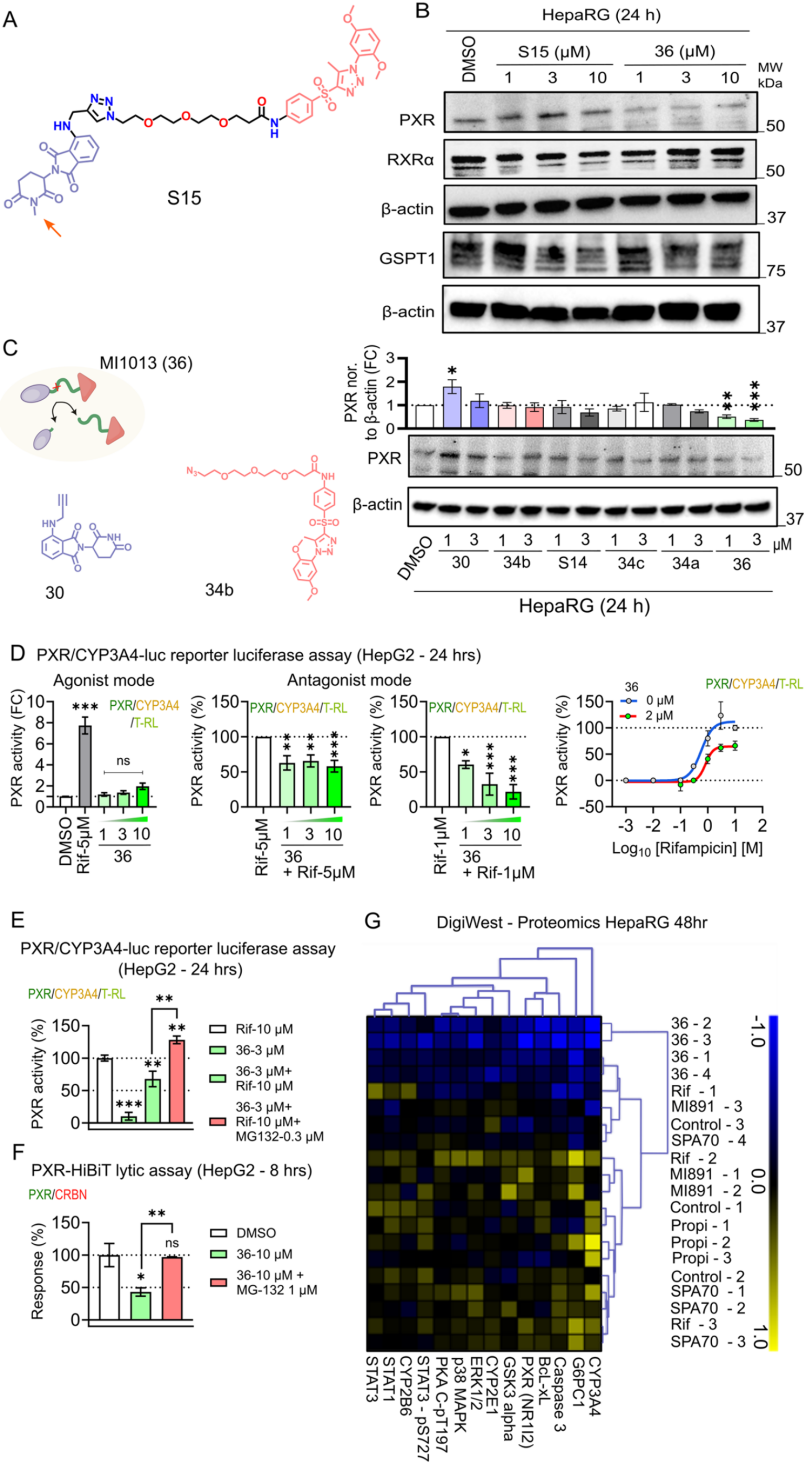
Functional proteasome-mediated degradation of PXR by MI1013 (**36**) PROTAC. (A) *N*-methylated MI1155 (S15) chemical structure. (B) Western blot analysis of PXR, retinoid X receptor α (RXRα), the heterodimeric partner of PXR, and GSPT1 protein expression in HepaRG cells treated with different concentrations of MI1013 and MI1155 for 24 h. (C) Left panel: Schematic representation of derivatives of MI1013, and chemical structures of MI1002B (blue) and MI1011B (red). Right panel: Western blot analysis of PXR protein expression in HepaRG cells treated with 1 and 3 μM concentrations of PROTAC derivatives for 24 h. (D) PXR agonist and antagonist activities were analyzed using the PXR-responsive luciferase reporter gene assay based on the *CYP3A4* gene promoter luciferase construct in HepG2 cells, and PXR activity is represented by PXR transcriptional activation in the assay. PXR agonistic activity was evaluated with 5 μM rifampicin as a positive control. Data are presented as mean ± SEM of the luciferase construct fold activation (FC) to vehicle-treated controls. ***p < 0.001; ns, not significant (Dunnett’s multiple comparison test; MI1013 (**36**) at different concentrations or 5 μM rifampicin vs DMSO). For antagonistic activity, MI1013 (**36**) was administered at specified concentrations alongside 1 or 5 μM rifampicin treatment, respectively. The positive controls treated with rifampicin are set as 100%. Data are presented as mean ± SEM ***p < 0.001; *p < 0.05 (Dunnett’s multiple comparison test). On the right panel, cells were treated with a concentration range of the PXR agonist rifampicin in the absence or presence of 2 μM MI1013 (**36**). Curve fitting (nonlinear regression) was performed using log­(inhibitor) vs response with a variable slope (four parameters). (E) The PXR-responsive luciferase reporter assay performed in HepG2 cells treated for 24 h with 3 μM MI1013 (**36**), with or without proteasome inhibitor MG132 1 μM and rifampicin, was used to evaluate the proteasome-mediated degradation effect. One-way ANOVA with multiple comparisons test (***p < 0.001). (F) Nanoglo HiBiT lytic assay. PXR-HiBiT/CRBN vectors (1:1 ratio, 0.3 ng/well) transfected HepG2 cells were treated for 8 h with 10 μM MI1013 (**36**), with or without 1 μM MG132. One-way ANOVA with multiple comparisons test (*p = 0.0184; ns, not significant). The relative luminescence of PXR-HiBit complex in control samples is set to 100% response. (G) Clustered heatmap of differentially expressed proteins and phosphoproteins in HepaRG. Protein levels were analyzed using DigiWest (bead-based multiplexed Western blotting) (at 48 h after rifampicin-agonist, SPA70 or MI891-antagonists, and MI1013 (**36**)-degrader treatment, *n* = 4, median-centered results, Wilcoxon Test, *p* < 0.05).

Next, we assessed the PXR transcriptional activity using the *CYP3A4* gene promoter luciferase construct, which is regulated by PXR, in transiently transfected HepG2 cells (Figure 8D,E). MI1013 demonstrated a concentration-dependent inhibition of PXR activation by rifampicin (Figure 8D). Notably, MI1013 did not exhibit agonistic activity at concentrations up to 10 μM (Figure 8D, left panels). When HepG2 cells were treated with the proteasome inhibitor MG132, the inhibition of PXR or PXR-HiBiT degradation caused by MI1013 was reversed, indicating the involvement of the proteasome pathway (Figure 8E,F). Further confirmation of PXR degradation by MI1013 was obtained by showing that MI1013 could counteract a ligand-mediated activation of the CYP3A4-based luciferase construct in LS174T cells (Figure S10A).

In other experiments, we employed the TR-FRET competitive binding assay with recombinant PXR LBD (Figure S8D). MI1013 exhibited weaker binding to PXR LBD compared to its precursor (MI1011B) and SR12813. These results indicate that MI1013 is less competitive in binding to PXR LBD in the presence of a highly binding PXR probe (tracer), likely due to multiple distinct binding conformations.^38^ MI1013 does not activate a mammalian two-hybrid system with either wild-type PXR LBD or obstructed PXR LBD (a triple mutant of PXR LBD) confirming the interaction between the MI1013 PROTAC and the PXR LBD (Figure S10B). Overall, these findings highlight the specificity and safety profile of the MI1013 PROTAC molecule in modulating *CYP3A4* promoter activity through PXR degradation. Importantly, cytotoxicity/viability studies revealed that MI1013 PROTAC was nontoxic up to a concentration of 100 μM in HK-2, COS-1, and HepG2 cells (Figure S8E).

These lines are used as sensitive models in cytotoxicity testing. Furthermore, no cytotoxic effect on the HepG2 cell line excludes false results in other experiments.

Next, we employed extensive protein profiling using a bead-based microarray multiplexed immunoassay platform (DigiWest) to evaluate protein panel expression and degradation, including expression of phosphorylated proteins of key signaling pathways (Figure 8G).^39^ Selected proteins and phosphoproteins in HepaRG cells were analyzed after 48 h of treatment with MI1013 (2 μM), MI891 (5 μM), rifampicin (5 μM), propiconazole (5 μM, a dual PXR and CAR agonist), and SPA70 (5 μM).

DigiWest analysis following MI1013 (**36**) treatment confirmed PXR degradation (Figure 8G). In a detailed analysis of the data with 57 antibodies (Figure S11), we did not observe statistically significant effects from the treatments. However, we noted a significant difference between the effects of PXR inducers and those of PXR antagonists/degraders (Wilcoxon test, *p* < 0.05, comparing rifampicin/propiconazole with MI891/MI1013/SPA70). Further statistical analysis revealed significant differences between the PROTAC data and all other samples (Wilcoxon test, *p* < 0.05) for several proteins (including PXR, CYP3A4, STAT1, STAT3, Caspase 3, Bcl-X, CYP2E1) and phosphoproteins (PKA C α/β/γ-phospho Thr197, and STAT3 phospho Ser 727) (Figures 8G and S11). These findings suggest a strong effect of MI1013 on PXR downregulation, as well as the expression of numerous genes involved in cellular signaling. The relationship between these effects and PXR degradation remains to be elucidated. Similarly, the effect of PXR degradation on hepatocyte cell signaling, especially the JAK-STAT cascade, should be further studied.

Finally, we examined the crosstalk of heterodimerization between PXR and CAR. The previous study by Bwayi et al. reported the inhibitory effect of PXR on CAR in cellular models that coexpress both proteins.^40^ We observed a similar inhibitory effect in our experiments (Figure S12A). Notably, when we used the MI1013 (**36**) PROTAC degrader in combination with CITCO, CAR activation was restored, an effect not seen with CITCO alone in HepG2 cells cotransfected with both receptors (Figure S12A). This suggests that the MI1013 PROTAC effectively modulates PXR activity, allowing CAR to regain its function.

#### Perturbation of Gene Expression of PXR Target Genes and Key Endobiotic Metabolism Genes by MI891 and MI1013 PROTAC in HepaRG Cells and Human Hepatocytes

The expression of various PXR target genes involved in drug metabolism and other pathways was analyzed through extensive qRT-PCR assays in HepaRG and PHH cells treated with PXR agonist rifampicin, propiconazole (PXR and CAR agonist), or CITCO (CAR agonist), along with PXR antagonists MI891 and SPA70, or the PXR degrader MI1013 (**36**).

We found that the key xenobiotic metabolism gene induced by PXR agonists, *CYP3A4*, was downregulated by antagonists or MI1013 (**36**), a PROTAC degrader. *CYP2B6*, the dominant CAR target gene, was downregulated by SPA70 but not MI891, suggesting that the weak CAR activation by MI891 may outweigh PXR antagonism in gene regulation (Figure 9A). All used PXR antagonists suppressed expression of *ABCB1* mRNA, which encodes P-glycoprotein transporter, a known PXR target gene that is upregulated by rifampicin. PXR antagonists also suppressed both CYP3A4 and ABCB1 genes in the presence of rifampicin (Figure 9A). Furthermore, MI891 antagonist and MI1013 PROTAC significantly downregulated *SULT1E1* and *SULT1B1* genes encoding sulfotransferases, *CYP7B1* (a key enzyme of bile acid synthesis in acidic pathway), and *NR0B2* (SHP, encoding small heterodimer partner) (Figure 9B,C). Interestingly, *UGT1A1* mRNA (encoding UDP glucuronosyltransferase 1 family, polypeptide A1 enzyme) was elevated after treatment with all PXR ligands (Figure 9A). In contrast, *CYP7A1* mRNA was downregulated after all treatments (Figure 9B).

**Figure 9:**
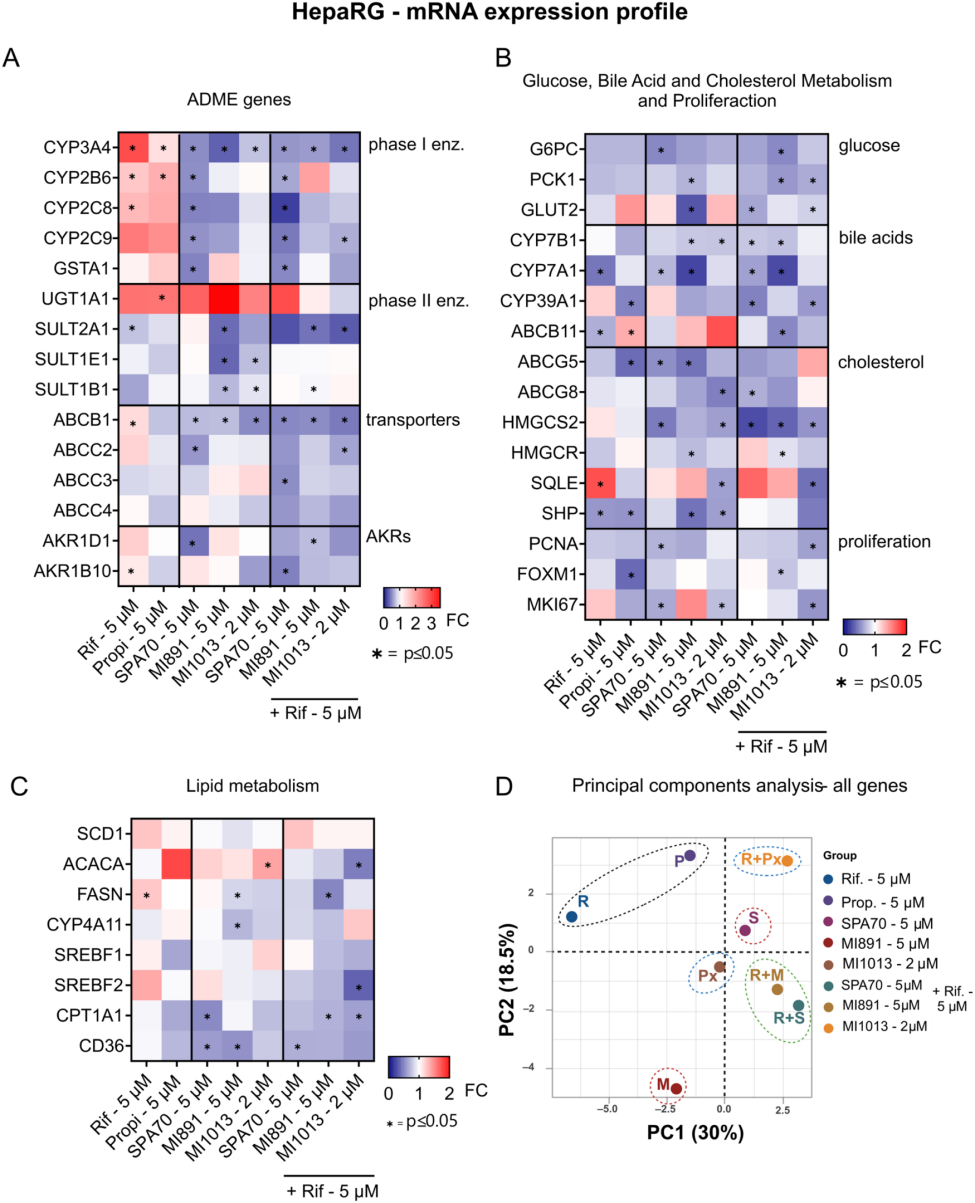
Gene expression analysis in HepaRG cells treated with PXR agonists, antagonists, or MI1013 degrader. (A) mRNA expression of ADME genes, (B) genes involved in glucose, bile acid, cholesterol metabolism, and proliferation, and (C) lipid metabolism gene expression in HepaRG cells. mRNA levels were analyzed using RT-qPCR (at 48 h after rifampicin-agonist, SPA70 or MI891-antagonists, and MI1013-degrader treatment). Rifampicin and propiconazole were used as prototypical inducers for PXR and PXR/CAR, respectively. Data are mean ± SD, *n* = 3 (*p < 0.05). FC, fold change. (D) Principal components analysis (PCA) of all genes (A–C) variables, quantitatively categorized. R, rifampicin; P, propiconazole; M, MI891; S, SPA70; Px, **36** (MI1013). Blue color-downregulation, red color-upregulation.

In principal component analysis (PCA) of PXR-regulated genes, rifampicin and propiconazole revealed similar regulatory patterns. In contrast, the antagonists MI891 and SPA70 exhibited distinct behaviors in the agonistic mode, but their patterns reversed under antagonistic conditions with rifampicin. Notably, MI1013 (**36**) PROTAC displayed unique behavior in both scenarios, showing no alignment with any other modulators (Figure 9D).

In subsequent experiments, we analyzed the effect of MI891 on the expression of similar gene sets in primary human hepatocytes from three donors. We found that MI891 suppressed the expression of *CYP3A4* mRNA and attenuated its induction by rifampicin (Figure 10). MI891 may slightly upregulate *CYP2B6* mRNA in all donors, suggesting the dominant role of CAR in the regulation of the gene. Importantly, we observed significant downregulation of *CYP7A1*, *HMGCS2*, *PCK1*, and *MKI67* mRNA after treatment with MI891, both in the presence and absence of rifampicin (Figure 10). The RT-qPCR data were cross-checked with previous microarray data (GSE90122) for the effects of rifampicin and SPA70. The comparison suggests that SPA70 consistently downregulates genes involved in drug disposition (such as CYP3A4, CYP2C9, CYP2B6, ABCB1, AKR1D1, and SULT2A1) in the data sets (Figure S12B,C).

**Figure 10:**
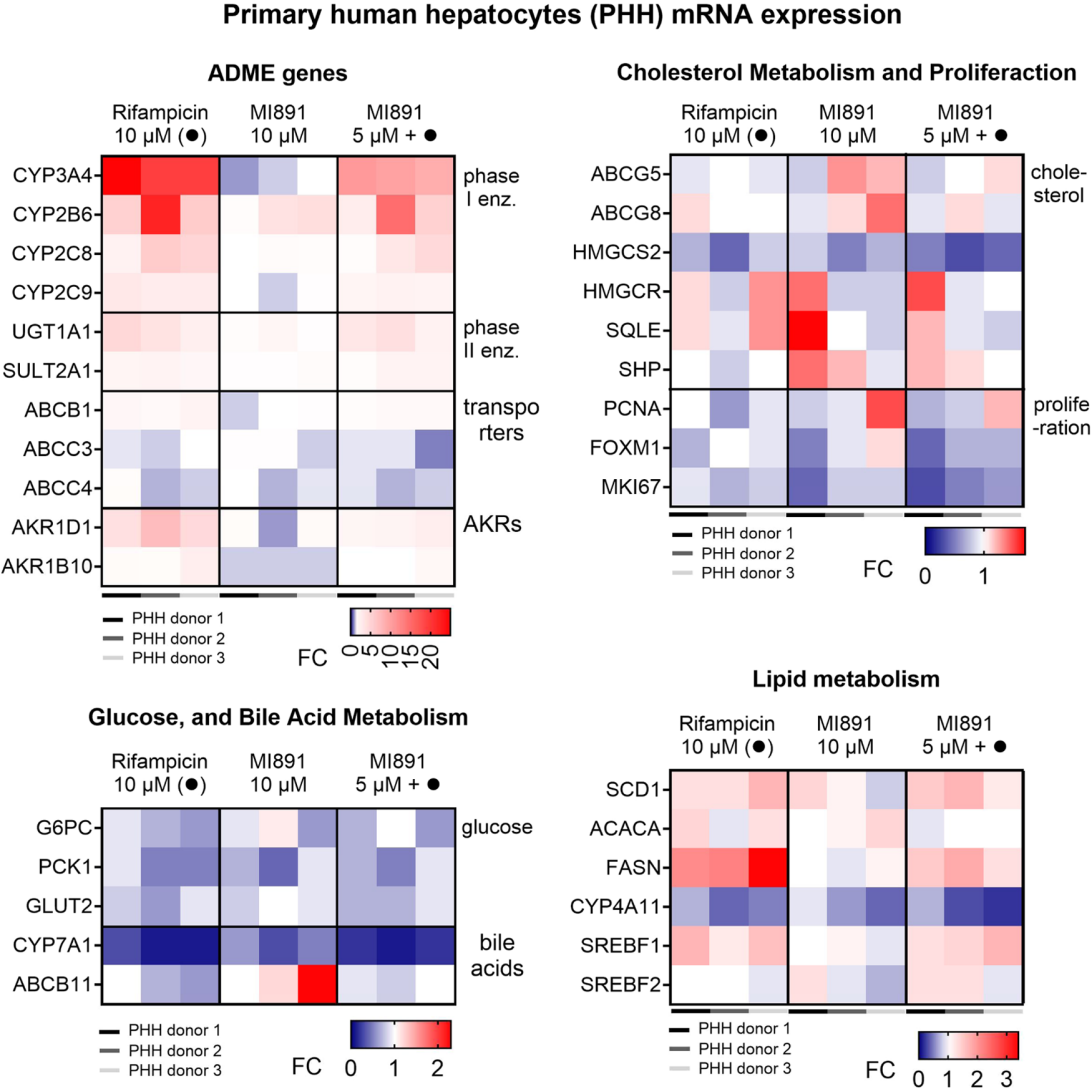
Primary human hepatocytes mRNA expression. Expression of ADME genes, genes involved in glucose and lipid metabolism, and proliferation have been studied in three donors. mRNA levels were analyzed using RT-qPCR after 48 h treatment with rifampicin (10 μM), MI891 (5 μM), or their combination. Data are means of three technical replicates (*n* = 3). FC, fold change. Red color-upregulation, blue color-downregulation.

Collectively, these data suggest that PXR antagonists and the MI1013 PROTAC degrader significantly affect some critical genes involved in sulfate conjugation (e.g., *SULT1E1*), bile acid synthesis (*CYP7A1*), gluconeogenesis (*PCK1*), ketone synthesis (*HMGCS2*), and hepatocyte proliferation (*MKI67*). Future experiments should investigate the effects of pharmacological inhibition or PXR degradation on the whole transcriptome using RNA sequencing in hepatocytes from additional donors or in *PXR*/*CYP3A4*-humanized mice.

## Discussion and Conclusions

The nuclear receptor PXR, functioning as chemosensors, instigates the transcriptional regulation of downstream genes implicated in drug metabolism and excretion. PXR is dominantly localized in the cytoplasm, where it binds ligands and is accessible for proteasomal degradation.^41^ Concurrently, emerging research underscores the key roles of PXR in coordinating the regulation of glucose metabolism and insulin resistance, as well as lipid, bile acid, and cholesterol metabolism and homeostasis.^26,42^

Its ligand-binding domain is bulky and flexible, interacting with a diverse array of ligands of varying sizes and shapes, leading to significant ligand promiscuity.^8^ These properties hindered the development of selective direct-acting PXR antagonists with high affinity for the PXR LBD until 2017.^11^ In 2024, Lin and colleagues introduced the first series of highly potent PXR antagonists based on the scaffolds of 1-substituted-phenyl-4-substituted-phenylsulfonyl-5-methyl-1*H*-1,2,3-triazole and 1-substituted-phenyl-4-substituted-phenylaminocarbonyl-1*H*-1,2,3-triazole.^15^

Herein, we report the discovery of a potent PXR antagonist, MI891 (compound **11**), based on the 2-chloro-5-((4-(2-(4-chlorophenyl)­imidazo­[1,2-*a*]­pyridin-3-yl)-1*H*-1,2,3-triazol-1-yl)­methyl)­benzamide scaffold. This structure has recently been identified as a ligand for the human constitutive androstane receptor (CAR, NR1I3) with suggested PXR inverse agonistic activity **(cmp 39)**.^25^ Based on the hit, we synthesized a small library of methylated 2-chlorobenzamides (ring D) with modified A, B, and D rings, and tested their interactions with human PXR and CAR receptors in this work.

We found that the substitution with a 4-chlorophenyl group (ring B), a 1,2,3-triazole linker (ring C), and a 3-amide 4-chlorophenyl group (ring D) is critical for PXR antagonism/inverse agonism (Table 1, Figure 4D). Substituting 4-chlorophenyl with pyridin-4-yl (ring B) abrogated the activity. Similarly, substitution of the 1,2,3-triazole C ring with 1,3,4-oxadiazol or thiadiazol resulted in the loss of activity (cmp **5**, **6**) or conversion into a PXR partial agonist (cmp **7**, Table 1). Importantly, methylation or dimethylation of the 3-amide progressively diminished PXR antagonistic activity, with compound 41*, previously reported by our group,^25^ emerging as a highly selective human CAR ligand devoid of significant activity against PXR. We also fluorinated ring A, previously shown to be a site of metabolic hydroxylation.^25^ We recently reported that fluorination at position 7 of imidazo­[1,2-*a*]­pyridine does not hinder CAR activation or PXR antagonism but metabolically stabilizes imidazo­[1,2-*a*]­pyridine for hydroxylation.^26^ Here, we demonstrate that the substitution of ring A with fluorine at positions 6 and 8 restricts CAR activation while maintaining the PXR antagonistic/inverse agonistic activities of compounds **8** and **10**–**12** (Table 1, Figure 4C,D). This is due to the hydrophobic interactions with C202 and Y326 of the CAR LBD, which are disturbed by fluorination in positions 6 and 8.^25,26^ Interestingly, we found only ligands exhibiting both common PXR antagonistic (inverse effect on PXR agonist rifampicin) and inverse agonistic (decrease in basal constitutive activity of PXR) activities, but not a pure neutral PXR antagonist without inverse agonistic activity. Compound **7** appeared as a PXR partial agonist (Table 1).

In cellular experiments, we demonstrate the activity of MI891 (compound **11**) in primary human hepatocytes, HepaRG cells, and HepG2 cells. MI891 effectively regulated a comprehensive set of typical PXR target genes, which have not been systematically examined with previously reported antagonist molecules, including SJPYT-331, SPA70, and compound 100.^10–12^ Comparing these antagonists, MI891 is the only currently available direct PXR antagonist that selectively abrogates the interaction between PXR and its coactivator, SRC1 (Figure 3E). Furthermore, MI891 was shown to be a specific PXR antagonist, not affecting other closely related nuclear receptors up to a concentration of 10 μM in either agonist or antagonist modes, as well as kinases, in an antagonist mode (Figure 5; Table S2).

We also found that MI891 (compound **11**) is a highly selective PXR antagonist/inverse agonist with sufficient stability in metabolically competent primary human hepatocytes (Figure 10). Additionally, we performed pilot microsomal stability experiments (Table S1) and pharmacokinetic studies in mice, along with expression analysis of hepatic target genes, demonstrating its applicability in *in vivo* experiments (unpublished data).

Moreover, we developed and evaluated a novel PXR degrader, MI1013 (**36**). We employed a systematic design and protein–protein docking with molecular simulations studies, revealing the distance from PXR to CRBN that was used to design the specific PROTAC degrader (Figure 6). This approach allowed us to minimize synthesis and testing methods, thereby reducing the cost and time for structure–activity relationship (SAR) studies. We demonstrate that MI1013 PROTAC can selectively degrade PXR protein via the ubiquitin-proteasome pathway (Figures 7 and 8E,F). Notably, we observed that the degradation efficiency of HiBiT-tagged PXR inversely correlated with its expression level and the amount of expression vector. Endogenous PXR was efficiently degraded (Figure 7A–C), while overexpression via a strong CMV promoter in our expression vector reduced relative degradation, likely due to saturation or altered protein dynamics (Figure S8C). In experiments using differentiated, phenotypically competent hepatic HepaRG cells or intestinal LS174T cells, we show that the PROTAC degrades endogenous PXR (Figures 7, 8, and S10A). The degradation kinetics assay showed a significant reduction in PXR protein levels at 12 h (Figure 7C), but the levels were restored after 72 h. These results confirm the reversible and long-lasting degradation of PXR. In addition, we show that the MI1013 PROTAC enhances the degradation of engineered eGFP- or HiBiT-tagged PXR hybrid proteins (Figures 7D, 8F, and S8C) while having no effect on the degradation of other proteins in MOLT4 cells (Figure 7E). Experiments with the tagged PXR fusion proteins further confirm MI1013-mediated degradation, even though they may not reflect the quantitative aspects of the degradation. Importantly, the MI1013 PROTAC suppresses rifampicin-induced upregulation of *CYP3A4*, as well as the basal expression of this gene in HepaRG cells (Figure 9A). In DigiWest multiplex protein profiling experiments, we found that MI1013 PROTAC suppresses several key proteins involved in cellular signaling pathways, including STAT1, STAT3, phospho Ser727 STAT3, caspase 3, Bcl-XL, GSK3α, Erk1/2, p38 and PKA Cα/β/γ phosphor Thr197 (Figure 8D).

The first PROTAC targeting PXR (JMV7048) in colon cancer stem cells was generated from the highly potent PXR agonist JMV6845 and thalidomide.^21,23^ Recently, Taosheng Chen’s group reported the generation of a protein degrader, SJPYT-195 from the PXR inhibitor SPA70.^20^ By incorporating an E3 ligase binding moiety, thalidomide, for CRBN, they aimed to degrade PXR through protein degradation pathways. However, SJPYT-195 acted as a molecular glue degrader of the translation termination factor GSPT1, leading to a subsequent reduction in PXR protein levels.^20^ This approach did not achieve the desired selectivity, highlighting the ongoing challenge in developing PXR-targeted degradation strategies. Additionally, in the paper, the authors found that varying short linker lengths did not result in specific degradation.^20^ This observation aligns with our computational study on linkerology, which aimed to develop the tailored PXR PROTAC molecule. Further optimization by the same team involved switching the E3 ligase target to VHL and conducting structure–activity relationship (SAR) studies, resulting in the identification of degrader 38*.^19^ The degrader 38* is composed of SJPYT-331 as the PXR warhead and VH032 as the anchor ligand for the VHL E3 ligase (Figure 1). Notably, degrader 38* was oriented through the H12 pore of PXR LBD,^19^ whereas our MI1013 PROTAC degrader was designed for the H2 pore of the PXR LBD (Figure 6A). This H2 pore is theoretically believed to be the more favorable entry site for PXR ligands,,^7,43^ although MD simulations are still needed to examine this hypothesis. Modification of the SPA70 tert-butyl group compromises key hydrophobic interactions with PXR (e.g., Trp299), leading to reduced binding and antagonism, as observed with compounds **34b** (Figures S8D and S9C). Another concern is the use of E3 ligases. Established PXR-degrading E3 ligases (Figure 6B) lack specific ligands. A comparison of CRBN and VHL indicates that achieving degradation in hepatocytes is challenging due to the higher expression levels of PXR compared to the E3 ligases. Specifically, CRBN is expressed at a 1:3 ratio relative to PXR transcript levels, while VHL being even less abundant (Figure S4B). This expression pattern is also observed in colorectal cancer, where both E3 ligase targets showed equivalent expression levels (Figure S4B).

In experiments with HepaRG and human hepatocytes cultivated as a monolayer in 2D conditions, we comprehensively examined the effects of MI891, MI1013 PROTAC, and SPA70 inhibitors alongside rifampicin and propiconazole. We observed opposing effects of PXR antagonists and agonists on the expression of typical ADME genes controlled by PXR, such as *CYP3A4*, *CYP2C9*, *CYP2C8*, *CYP2B6*, and *ABCB1* (Figure 9A). For the first time, we describe significant and consistent effects of PXR antagonists and the PXR degrader MI1013 on critical genes involved in bile acid synthesis (*CYP7B1* and *CYP7A1*), ketone synthesis (*HMGCS2*), and hepatocyte proliferation (*MKI67*) (Figures 9 and 10). Many other genes were also consistently downregulated by all PXR inhibitors, although their effects were not statistically significant (Figures 9 and 10). Thus, our results reveal previously unreported effects of PXR antagonism on many genes and pathways of hepatocytes, which may pave the way for the discovery of new molecules targeting PXR in metabolic diseases. These findings also demonstrate that targeting PXR with PROTAC degraders is a promising strategy to overcome the limitations of traditional PXR antagonists and exploit the therapeutic potential of PXR inhibition. Our ongoing animal studies with humanized PXR animal models should further validate the metabolic effects of the novel compounds. This will mainly involve studies in animal models under nutritional stress (e.g., a high-fat diet) or when the activation of PXR by ligands or genetic mutations leads to obesity, metabolic syndrome, liver steatosis, hypercholesterolemia, decreased insulin resistance connected with hyperglycemia, or hemorrhagic shock-induced liver injury.^1^ PXR antagonists may also prevent drug–drug interaction mediated by unwanted PXR activation or chemoresistance of some tumors.^1,4^

The discovery of the two novel PXR modulators opens up new possibilities for the selective modulation of PXR, which could have significant implications for the development of therapeutic agents. The specificity of MI891, as demonstrated by the absence of off-target effects on other nuclear receptors, underscores its potential as a lead compound for further drug development. This study is also the first to report a degrader of PXR that can effectively degrade this nuclear receptor in various cellular contexts of human differentiated hepatocyte models.

## Experimental Section

### Chemical and Materials

Rifampicin (prototype ligands of human PXR), SPA70 (PXR antagonist), propiconazole (CAR and PXR agonist), CITCO (a human CAR agonist), and dimethyl sulfoxide (DMSO) were all obtained from Merck (Darmstadt, Germany). The stock solutions 1000 × were prepared in DMSO. The final concentration of DMSO in media did not exceed 0.1% (v/v) in any experiments.

### General Chemical Procedures

NMR spectra were measured on a Bruker Avance II-600 and/or Bruker Avance II-500 instruments (600.1 or 500.0 MHz for 1H and 150.9 or 125.7 MHz for 13C) in hexadeuterodimethyl sulfoxide and referenced to the solvent signal (δ 2.50 and 39.70, respectively). Mass spectra were measured on a LTQ Orbitrap XL (Thermo Fischer Scientific) using electrospray ionization (ESI) and a GCT Premier (Waters) using EI. The elemental analyses were obtained on a PerkinElmer CHN Analyzer 2400, Series II Sys (PerkinElmer) and X-ray fluorescence spectrometer SPECTRO iQ II (SPECTRO Analytical Instruments, Germany). Column chromatography and thin-layer chromatography (TLC) were performed using Silica gel 60 (Fluka) and Silufol Silica gel 60 F254 foils (Merck), respectively. Solvents were evaporated at 2 kPa and bath temperature 30–60 °C. The compounds were dried at 13 Pa and 50 °C.

### Synthesis of Novel Ligands

#### General Procedure I. Cyclization of Heterocycle

2-Aminopyridine **13** or mono/difluorinated 2-aminopyridine **22a**–**d** was dissolved in EtOH and 2-bromo-1-(4-chlorophenyl)­ethan-1-one (1 equiv) or alternatively 2-bromo-1-(4-chlorophenyl)­ethan-1-one, 2-bromo-1-(pyridin-4-yl)­ethan-1-one was added followed by an addition of NaHCO_3_ (1 equiv). The reaction mixture was heated at 70 °C overnight. After the completion of the reaction (monitored by TLC or UPLC), the solvent was evaporated to a minimal volume, a residue was diluted with EtOAc and washed with water. Water phase was extracted twice more with EtOAc, combined organic phases were dried over sodium sulfate and evaporated. The residue was purified by flash column chromatography (eluent petrolether/EtOAc) providing compounds **14**, **15**, **16**, and **23a**–**d**.

#### 2-(Pyridin-4-yl)­imidazo­[1,2-*a*]­pyridine (Intermediate Compound) (**15**)

The title compound was prepared according to General Procedure I (Scheme 1). Mobile phase petrolether/EtOAc (40–100%). Yield 98 mg (48%). ^1^H NMR (401 MHz, DMSO-*d*_6_) δ 8.64–8.59 (m, 3H), 8.56 (dt, *J =* 6.8, 1.2 Hz, 1H), 7.94–7.86 (m, 2H), 7.62 (dq, *J =* 9.1, 1.0 Hz, 1H), 7.30 (ddd, *J =* 9.1, 6.7, 1.3 Hz, 1H), 6.94 (td, *J =* 6.8, 1.2 Hz, 1H). ^13^C NMR (101 MHz, DMSO) δ 150.33, 145.29, 141.82, 141.26, 127.41, 125.95, 120.08, 117.18, 113.03, 111.62. HRMS: calcd for [M + H], 196.08692; found, 196.08678.

#### 2-(4-Bromophenyl)­imidazo­[1,2-*a*]­pyridine (Intermediate Compound) (**16**)

The title compound was prepared according to General Procedure I (Scheme 1). Mobile phase petrolether/EtOAc (20–70%). Yield 1.87 g (94%). ^1^H NMR (401 MHz, DMSO-*d*_6_) δ 8.52 (dt, *J =* 6.8, 1.2 Hz, 1H), 8.43 (d, *J =* 0.7 Hz, 1H), 7.98–7.86 (m, 2H), 7.65–7.60 (m, 2H), 7.58 (dq, *J =* 9.1, 1.0 Hz, 1H), 7.25 (ddd, *J =* 9.1, 6.7, 1.3 Hz, 1H), 6.90 (td, *J =* 6.7, 1.2 Hz, 1H). ^13^C NMR (101 MHz, DMSO) δ 145.04, 143.33, 133.38, 131.81, 127.71, 127.13, 125.38, 120.85, 116.85, 112.61, 109.68. HRMS: calcd for [M + H], 273.0027; found, 273.0028.

#### 2-(4-Cyclopropylphenyl)­imidazo­[1,2-*a*]­pyridine (Intermediate Compound) (**17**)

The title compound was prepared from compound **16** (0.1 g, 0.36 mmol), which was dissolved in H_2_O/CH_3_CN (4 mL, 1:1) mixture and cyclopropylboronic acid (1.5 equiv) was added. The reaction mixture was degassed and refilled with argon. Subsequently K_3_PO_4_ (3.5 equiv), Pd­(OAc)_2_ (5 mol %) and Pcy_3_ (10 mol %) were added and the reaction mixture was stirred at 110 °C under argon. After completion of the reaction, the mixture was filtrated over Celite, extracted with EtOAc, the organic phase was dried over sodium sulfate and evaporated. The residue was purified by flash column chromatography. Mobile phase petrolether/EtOAc (20–70%). Yield 86 mg (73%). ^1^H NMR (401 MHz, DMSO-*d*_6_) δ 8.50 (dt, *J =* 6.8, 1.3 Hz, 1H), 8.33 (s, 1H), 7.85–7.80 (m, 2H), 7.55 (dq, *J =* 9.1, 1.0 Hz, 1H), 7.22 (ddd, *J =* 9.1, 6.7, 1.3 Hz, 1H), 7.16–7.10 (m, 2H), 6.87 (td, *J =* 6.7, 1.2 Hz, 1H), 1.94 (tt, *J =* 8.4, 5.1 Hz, 1H), 1.00–0.92 (m, 2H), 0.74–0.66 (m, 2H). ^13^C NMR (101 MHz, DMSO) δ 144.88, 144.61, 143.51, 131.17, 126.93, 125.78, 125.71, 125.66, 124.93, 116.66, 112.31, 108.75, 15.17, 9.67. HRMS: calcd for [M + H], 235.1235; found, 235.1234.

#### 2-(4-Chlorophenyl)-8-fluoroimidazo­[1,2-*a*]­pyridine (Intermediate Compound) (**23a**)

The title compound was prepared according to General Procedure I. Mobile phase petrolether/EtOAc (20–70%). Yield 1.01 g (93%). ^1^H NMR (401 MHz, DMSO-*d*_6_) δ 8.55 (d, *J =* 3.1 Hz, 1H), 8.39 (dd, *J =* 6.7, 0.9 Hz, 1H), 8.02–7.94 (m, 2H), 7.54–7.45 (m, 2H), 7.15 (ddd, *J =* 11.4, 7.7, 0.9 Hz, 1H), 6.87 (ddd, *J =* 7.7, 6.8, 4.7 Hz, 1H). ^13^C NMR (101 MHz, DMSO-*d*_6_) δ 150.88 (d, *J =* 249.1 Hz), 143.85, 137.77 (d, *J =* 28.4 Hz), 132.93, 132.69, 129.27, 127.84, 124.17 (d, *J =* 4.8 Hz), 112.02 (d, *J =* 6.8 Hz), 111.77–111.62 (m), 108.35 (d, *J =* 16.1 Hz). HRMS: calcd for [M + H], 247.0438; found, 247.0439.

#### 2-(4-Chlorophenyl)-7-fluoroimidazo­[1,2-*a*]­pyridine (Intermediate Compound) (**23b**)

The title compound was prepared according to General Procedure I. Mobile phase petrolether/EtOAc (20–70%). Yield 510 mg (83%). ^1^H NMR (401 MHz, DMSO-*d*_6_) δ 8.59 (ddd, *J =* 7.5, 5.9, 0.8 Hz, 1H), 8.40 (d, *J =* 0.7 Hz, 1H), 8.01–7.89 (m, 2H), 7.51–7.46 (m, 2H), 7.43 (ddt, *J =* 10.1, 2.7, 0.8 Hz, 1H), 6.97 (td, *J =* 7.6, 2.6 Hz, 1H). ^13^C NMR (101 MHz, DMSO-*d*_6_) δ 160.04 (d, *J =* 248.4 Hz), 145.15 (d, *J =* 14.3 Hz), 144.32 (d, *J =* 1.5 Hz), 132.72, 132.41, 129.14 (d, *J =* 11.1 Hz), 128.94, 104.74 (d, *J =* 29.4 Hz), 100.36 (d, *J =* 23.7 Hz). HRMS: calcd for [M + H], 247.0438; found, 247.0439.

#### 2-(4-Chlorophenyl)-6-fluoroimidazo­[1,2-*a*]­pyridine (Intermediate Compound) (**23c**)

The title compound was prepared according to General Procedure I. Mobile phase petrolether/EtOAc (20–70%). Yield 1.2 g (91%). ^1^H NMR (401 MHz, DMSO-*d*_6_) δ 8.75 (ddd, *J =* 4.6, 2.5, 0.8 Hz, 1H), 8.41 (d, *J =* 0.7 Hz, 1H), 8.00–7.94 (m, 2H), 7.64 (ddt, *J =* 9.9, 5.4, 0.8 Hz, 1H), 7.52–7.45 (m, 2H), 7.34 (ddd, *J =* 9.9, 8.4, 2.5 Hz, 1H). ^13^C NMR (101 MHz, DMSO-*d*_6_) δ 152.79 (d, *J =* 232.7 Hz), 144.61 (d, *J =* 2.0 Hz), 143.00, 132.73, 132.49, 128.97, 127.43, 117.57 (d, *J =* 9.4 Hz), 117.16 (d, *J =* 25.9 Hz), 113.84 (d, *J =* 41.4 Hz), 111.16 (d, *J =* 2.3 Hz). HRMS: calcd for [M + H], 247.0438; found, 247.0439.

#### 2-(4-Chlorophenyl)-6,8-difluoroimidazo­[1,2-*a*]­pyridine (Intermediate Compound) (**23d**)

The title compound was prepared according to General Procedure I. Mobile phase petrolether/EtOAc (20–70%). Yield 0.45 g (87%). ^1^H NMR (401 MHz, DMSO-*d*_6_) δ 8.70 (ddd, *J =* 4.3, 2.1, 0.9 Hz, 1H), 8.55 (d, *J =* 3.1 Hz, 1H), 8.01–7.96 (m, 2H), 7.57–7.48 (m, 3H). ^13^C NMR (101 MHz, DMSO-*d*_6_) δ 151.59 (dd, *J =* 233.31, 11.12 Hz), 150.03 (dd, *J =* 252.4, 14.9 Hz), 144.75 (d, *J =* 1.7 Hz), 135.76 (d, *J =* 27.8 Hz), 133.11, 132.41, 129.31, 127.86, 113.25 (d, *J =* 2.6 Hz), 111.38 (dd, *J =* 41.5, 5.9 Hz), 102.38 (dd, *J =* 30.3, 20.3 Hz). HRMS: calcd for [M + H], 265.0344; found, 265.0343.

#### General Procedure II. Iodination

2-Substituted imidazo­[1,2-*a*]­pyridines **15**–**17** and **23a**–**d** were dissolved in CH_3_CN (5 mL/mmol) and NIS (1.05 equiv) was added in one portion. The suspension was stirred at r.t. and the conversion was monitored by TLC. After the completion of the reaction (1 h), the reaction mixture was evaporated to a minimal volume, afterward it was diluted with EtOAc and washed with saturated Na_2_S_2_O_3_ solution. Inorganic phase was extracted twice more with EtOAc, combined organic phases were dried over sodium sulfate and evaporated. Alternatively, the suspension was filtered, washed with acetonitrile and the filtrate evaporated, diluted with EtOAc and washed with saturated solution of Na_2_S_2_O_3_. The residue was purified by flash column chromatography, mobile phase petrolether/EtOAc (10–50%) providing compounds **24a**–**d** and **38**–**40**.

#### 2-(4-Chlorophenyl)-8-fluoro-3-iodoimidazo­[1,2-*a*]­pyridine (Intermediate Compound) (**24a**)

The title compound was prepared according to General Procedure II. Mobile phase petrolether/EtOAc (10–50%). Yield: 0.35 g (92%). ^1^H NMR (401 MHz, DMSO-*d*_6_) δ 8.30 (dd, *J =* 6.9, 0.9 Hz, 1H), 8.11–8.04 (m, 2H), 7.63–7.55 (m, 2H), 7.32 (ddd, *J =* 11.0, 7.7, 0.9 Hz, 1H), 7.06 (ddd, *J =* 7.6, 6.9, 4.8 Hz, 1H). ^13^C NMR (101 MHz, DMSO-*d*_6_) δ 150.29 (d, *J =* 250.5 Hz), 145.73, 139.66 (d, *J =* 28.7 Hz), 133.22, 132.30, 129.80, 128.69, 124.18 (d, *J =* 4.8 Hz), 112.71 (d, *J =* 7.1 Hz), 109.01 (d, *J =* 16.0 Hz), 66.13. HRMS: calcd for [M + H], 372.9405; found, 372.9407.

#### 2-(4-Chlorophenyl)-7-fluoro-3-iodoimidazo­[1,2-*a*]­pyridine (Intermediate Compound) (**24b**)

The title compound was prepared according to General Procedure II. Mobile phase petrolether/EtOAc (10–50%). Yield: 0.32 g (93%). ^1^H NMR (401 MHz, DMSO-*d*_6_) δ 8.49 (t, *J =* 6.6 Hz, 1H), 8.07 (d, *J =* 8.2 Hz, 2H), 7.57 (dd, *J =* 9.0, 6.0 Hz, 3H), 7.12 (td, *J =* 7.6, 2.6 Hz, 1H). ^13^C NMR (101 MHz, DMSO-*d*_6_) δ 160.72 (d, *J =* 249.8 Hz), 147.16 (d, *J =* 14.2 Hz), 146.55, 133.05, 132.58, 129.57, 128.94, 128.68, 127.37, 105.83 (d, *J =* 29.7 Hz), 100.73 (d, *J =* 23.8 Hz), 63.43. HRMS: calcd for [M + H], 372.9405; found, 372.9407.

#### 2-(4-Chlorophenyl)-6-fluoro-3-iodoimidazo­[1,2-*a*]­pyridine (Intermediate Compound) (**24c**)

The title compound was prepared according to General Procedure II. Mobile phase petrolether/EtOAc (10–50%). Yield: 0.29 g (92%). ^1^H NMR (401 MHz, DMSO-*d*_6_) δ 8.57 (ddd, *J =* 4.6, 2.4, 0.8 Hz, 1H), 8.09–8.03 (m, 2H), 7.73 (ddd, *J =* 9.8, 5.3, 0.8 Hz, 1H), 7.60–7.55 (m, 2H), 7.47 (ddd, *J =* 9.8, 8.3, 2.4 Hz, 1H). ^13^C NMR (101 MHz, DMSO-*d*_6_) δ 153.66 (d, *J =* 235.2 Hz), 146.82 (d, *J =* 2.3 Hz), 145.33, 133.08, 132.63, 129.65, 128.68, 118.23 (d, *J =* 13.6 Hz), 118.05 (d, *J =* 3.0 Hz), 114.46 (d, *J =* 42.7 Hz), 65.73. HRMS: calcd for [M + H], 372.9405; found, 372.9406.

#### 2-(4-Chlorophenyl)-6,8-difluoro-3-iodoimidazo­[1,2-*a*]­pyridine (Intermediate Compound) (**24d**)

The title compound was prepared according to General Procedure II. Mobile phase petrolether/EtOAc (10–50%). Yield: 0.345 g (94%). ^1^H NMR (401 MHz, DMSO-*d*_6_) δ 8.49 (ddd, *J =* 4.4, 2.1, 0.9 Hz, 1H), 8.07–8.01 (m, 2H), 7.67 (ddd, *J =* 11.1, 9.2, 2.1 Hz, 1H), 7.59–7.52 (m, 2H). ^13^C NMR (101 MHz, DMSO-*d*_6_) δ 151.95 (dd, *J =* 235.62 Hz, *J =* 11.32 Hz), 149.72 (dd, *J =* 253.9, 14.6 Hz), 146.63 (d, *J =* 1.9 Hz), 137.70 (d, *J =* 28.1 Hz), 133.34, 132.10, 129.72, 128.71, 111.71 (dd, *J =* 42.6, 5.8 Hz), 103.00 (dd, *J =* 30.2, 20.3 Hz), 68.18 (d, *J =* 2.5 Hz). HRMS: calcd for [M + H], 390.9311; found, 390.9312.

#### 2-(4-Chlorophenyl)-3-iodoimidazo­[1,2-*a*]­pyridine (Intermediate Compound) (**38**)

The title compound was prepared according to General Procedure II (Scheme 1). Mobile phase petrolether/EtOAc (10–50%). Yield: 352 mg (98%). ^1^H NMR (401 MHz, DMSO-*d*_6_) δ 8.42 (dt, *J =* 6.9, 1.2 Hz, 1H), 8.11–8.06 (m, 2H), 7.63 (dt, *J =* 9.0, 1.2 Hz, 1H), 7.60–7.55 (m, 2H), 7.38 (ddd, *J =* 9.0, 6.8, 1.3 Hz, 1H), 7.09 (td, *J =* 6.8, 1.2 Hz, 1H). ^13^C NMR (101 MHz, CDCl3) δ 147.52, 145.52, 132.99, 132.85, 129.73, 128.67, 127.35, 126.47, 117.16, 113.93, 63.82. HRMS: calcd for [M + H], 354.94935; found, 354.94944.

#### 3-Iodo-2-(pyridin-4-yl)­imidazo­[1,2-*a*]­pyridine (Intermediate Compound) (**39**)

The title compound was prepared according to General Procedure II. Mobile phase petrolether/EtOAc (10–50%). Yield: 253 mg (87%). ^1^H NMR (401 MHz, DMSO-*d*_6_) δ 8.71–8.66 (m, 2H), 8.43 (dt, *J =* 6.9, 1.2 Hz, 1H), 8.10–8.05 (m, 2H), 7.65 (dt, *J =* 9.1, 1.1 Hz, 1H), 7.39 (ddd, *J =* 9.0, 6.7, 1.2 Hz, 1H), 7.09 (td, *J =* 6.8, 1.2 Hz, 1H).^13^C NMR (101 MHz, DMSO) δ 150.37, 147.91, 143.89, 141.53, 127.75, 127.17, 122.29, 117.73, 114.54, 65.91. HRMS: calcd for [M + H], 321.98357; found, 321.98367.

#### 2-(4-Cyclopropylphenyl)-3-iodoimidazo­[1,2-*a*]­pyridine (Intermediate Compound) (**40**)

The title compound was prepared according to General Procedure II. Mobile phase petrolether/EtOAc (10–50%). Yield: 0.287 g (99%). ^1^H NMR (401 MHz, DMSO-*d*_6_) δ 8.40 (d, *J =* 6.9 Hz, 1H), 7.95 (d, *J =* 8.0 Hz, 2H), 7.61 (d, *J =* 9.0 Hz, 1H), 7.35 (t, *J =* 7.9 Hz, 1H), 7.19 (d, *J =* 7.9 Hz, 2H), 7.06 (t, *J =* 6.9 Hz, 1H), 1.97 (td, *J =* 8.5, 4.3 Hz, 1H), 0.98 (d, *J =* 7.7 Hz, 2H), 0.73 (d, *J =* 5.4 Hz, 2H). ^13^C NMR (101 MHz, DMSO) δ 147.37, 146.72, 144.04, 130.89, 127.98, 127.13, 126.06, 125.43, 116.95, 113.62, 62.86, 15.18, 9.83. HRMS: calcd for [M + H], 361.0202; found, 361.0206.

#### General Procedure III. Sonogashira Coupling

3-Iodoimidazo­[1,2-*a*]­pyridines **24a**–**d** and **38**–**40** were placed in dried round-bottom flask, diluted with dry DMF and degassed at 0 °C and flushed with argon. CuI (10 mol %), Pd­(PPh_3_)_2_Cl_2_ (5 mol %) were added and the mixture was properly degassed and dry TEA (3 equiv) was added and the mixture degassed again. Finally, TMS-acetylene (5 equiv) was added in one portion. The reaction mixture was stirred at r.t. under the argon atmosphere. After the completion of the reaction (monitored by TLC), the mixture was if necessary diluted with CHCl_3_ and filtered over Celite. Filtrate was washed with water, water phase was extracted twice more with CHCl_3_, combined organic phases were dried over sodium sulfate and evaporated. A residue was purified by flash column chromatography (mobile phase petrolether/EtOAc) providing compounds **25a**–**d** and **41**–**43**.

#### 2-(4-Chlorophenyl)-8-fluoro-3-((trimethylsilyl)­ethynyl)­imidazo­[1,2-*a*]­pyridine (Intermediate Compound) (**25a**)

The title compound was prepared according to General Procedure III. Mobile phase petrolether/EtOAc (10–50%). Yield: 125 mg (76%). ^1^H NMR (401 MHz, DMSO-*d*_6_) δ 8.28–8.21 (m, 3H), 7.59–7.54 (m, 2H), 7.36 (ddd, *J =* 11.0, 7.8, 0.9 Hz, 1H), 7.09 (ddd, *J =* 7.7, 6.7, 4.6 Hz, 1H), 0.35 (s, 9H). ^13^C NMR (101 MHz, DMSO-*d*_6_) δ 150.88 (d, *J =* 250.2 Hz), 146.41, 137.25 (d, *J =* 28.8 Hz), 134.04, 131.82, 129.27, 128.67, 122.87 (d, *J =* 4.7 Hz), 113.82 (d, *J =* 7.0 Hz), 110.96 (d, *J =* 16.0 Hz), 109.84, 106.00, 92.94. HRMS: calcd for [M + H], 343.0834; found, 343.0836.

#### 2-(4-Chlorophenyl)-7-fluoro-3-((trimethylsilyl)­ethynyl)­imidazo­[1,2-*a*]­pyridine (Intermediate Compound) (**25b**)

The title compound was prepared according to General Procedure III. Mobile phase petrolether/EtOAc (10–50%). Yield: 171 mg (58%). ^1^H NMR (401 MHz, DMSO-*d*_6_) δ 8.44 (dd, *J =* 7.5, 5.6 Hz, 1H), 8.25–8.19 (m, 2H), 7.64–7.58 (m, 1H), 7.57–7.53 (m, 2H), 7.16 (td, *J =* 7.6, 2.6 Hz, 1H), 0.34 (s, 9H). ^13^C NMR (101 MHz, DMSO-*d*_6_) δ 161.38 (d, *J =* 251.3 Hz), 147.05, 145.42, 133.58, 131.78, 128.97, 128.23, 109.11, 106.21 (d, *J =* 29.3 Hz), 101.39 (d, *J =* 24.0 Hz), 92.91, −0.12. HRMS: calcd for [M + H], 343.0834; found, 343.0833.

#### 2-(4-Chlorophenyl)-6-fluoro-3-((trimethylsilyl)­ethynyl)­imidazo­[1,2-*a*]­pyridine (Intermediate Compound) (**25c**)

The title compound was prepared according to General Procedure III. Mobile phase petrolether/EtOAc (10–50%). Yield: 74 mg (40%). ^1^H NMR (401 MHz, DMSO-*d*_6_) δ 8.54 (ddd, *J =* 4.0, 2.5, 0.8 Hz, 1H), 8.28–8.18 (m, 2H), 7.77 (ddd, *J =* 9.8, 5.1, 0.8 Hz, 1H), 7.61–7.50 (m, 3H), 0.35 (s, 9H). ^13^C NMR (101 MHz, DMSO-*d*_6_) δ 153.80 (d, *J =* 236.5 Hz), 147.10, 142.48, 133.58, 131.85, 128.96, 128.24, 119.52 (d, *J =* 25.6 Hz), 118.26 (d, *J =* 9.0 Hz), 112.95 (d, *J =* 41.3 Hz), 109.91, 92.54, −0.15. HRMS: calcd for [M + H], 343.0834; found, 343.0835.

#### 2-(4-Chlorophenyl)-6,8-difluoro-3-((trimethylsilyl)­ethynyl)­imidazo­[1,2-*a*]­pyridine (Intermediate Compound) (**25d**)

The title compound was prepared according to General Procedure III and used as crude for the next step without further purification.

#### 2-(4-Chlorophenyl)-3-((trimethylsilyl)­ethynyl)­imidazo­[1,2-*a*]­pyridine (Intermediate Compound) (**41**)

The title compound was prepared according to General Procedure III. Mobile phase petrolether/EtOAc (10–50%). Yield: 625 mg (56%). ^1^H NMR (401 MHz, DMSO-*d*_6_) δ 8.41 (dt, *J =* 6.8, 1.2 Hz, 1H), 8.28–8.21 (m, 2H), 7.70 (dt, *J =* 9.0, 1.1 Hz, 1H), 7.59–7.53 (m, 2H), 7.46 (ddd, *J =* 9.0, 6.8, 1.3 Hz, 1H), 7.14 (td, *J =* 6.8, 1.2 Hz, 1H), 0.34 (s, 9H). ^13^C NMR (101 MHz, DMSO) δ 146.17, 144.70, 133.43, 132.04, 128.88, 128.28, 127.82, 125.82, 117.30, 114.28, 108.95, 104.06, 93.27, −0.10. HRMS: calcd for [M + H], 325.09223; found, 325.09232.

#### 2-(Pyridin-4-yl)-3-((trimethylsilyl)­ethynyl)­imidazo­[1,2-*a*]­pyridine (Intermediate Compound) (**42**)

The title compound was prepared according to General Procedure III. Mobile phase cy/EtOAc (20–60%). Yield 431 mg (50%). ^1^H NMR (401 MHz, CDCl3) δ 8.75–8.68 (m, 2H), 8.48 (dt, *J =* 7.0, 1.1 Hz, 1H), 7.99–7.93 (m, 2H), 7.51 (dt, *J =* 9.0, 1.1 Hz, 1H), 7.21 (ddd, *J =* 9.0, 6.8, 1.1 Hz, 1H), 6.86 (td, *J =* 6.8, 1.2 Hz, 1H), 0.18 (s, 9H). ^13^C NMR (101 MHz, CDCl3) δ 149.82, 145.13, 143.93, 138.76, 126.27, 126.19, 116.63, 114.24, 110.68, 107.03, 87.53, 3.56. LC-MS: calcd for [M + H], 292.126, found, 292.098.

#### 2-(4-Cyclopropylphenyl)-3-((trimethylsilyl)­ethynyl)­imidazo­[1,2-*a*]­pyridine (Intermediate Compound) (**43**)

The title compound was prepared according to General Procedure III. Mobile phase petrolether/EtOAc (10–50%). Yield: 211 mg (93%). ^1^H NMR (401 MHz, DMSO-*d*_6_) δ 8.38 (dt, *J =* 6.8, 1.2 Hz, 1H), 8.19–8.11 (m, 2H), 7.68 (dt, *J =* 9.0, 1.1 Hz, 1H), 7.42 (ddd, *J =* 9.0, 6.8, 1.3 Hz, 1H), 7.22–7.14 (m, 2H), 7.11 (td, *J =* 6.8, 1.2 Hz, 1H), 1.99–1.89 (m, 1H), 1.02–0.90 (m, 2H), 0.77–0.68 (m, 2H), 0.34 (s, 9H). ^13^C NMR (101 MHz, DMSO) δ 147.62, 144.87, 144.64, 130.25, 127.43, 126.63, 125.61, 117.10, 113.95, 108.37, 103.39, 93.85, 15.25, 9.90, −0.04. HRMS: calcd for [M + H], 331.1631; found, 331.1630.

#### General Procedure IV. Click Reaction

Trimethylsilyl­(ethynyl)­imidazo­[1,2-*a*]­pyridine derivatives **25a**–**d** and **41**–**43** were dissolved in THF/H_2_O mixture (1:1) and 5-(azidomethyl)-2-chlorobenzamide or 5-(azidomethyl)-2-chloro-*N*-methylbenzamide (1 equiv) was added. The reaction mixture was degassed at 0 °C, refilled with argon and CuSO_4_·5H_2_O (10 mol %), KF (1 equiv), sodium ascorbate (1 equiv) were added in one portion. The reaction mixture was stirred at r.t. and monitored by TLC. After the completion of the reaction, the mixture was diluted with EtOAc and washed with water. The water phase was extracted twice more with EtOAc, combined organic phases were dried over sodium sulfate and evaporated. The residue was purified by flash column chromatography (eluent petrolether/EtOAc or EtOAc/MeOH) providing final compounds **2**–**4** and **8**–**12**.

#### 2-Chloro-5-(1-(4-(2-(4-chlorophenyl)­imidazo­[1,2-*a*]­pyridin-3-yl)-1*H*-1,2,3-triazol-1-yl)­ethyl)­benzamide (**2**)

The title compound was prepared according to General Procedure IV (Scheme 1). Mobile phase petrolether/EtOAc (40–100%). Yield 56 mg (81%).^1^H NMR (401 MHz, DMSO-*d*_6_) δ 8.67 (s, 1H), 8.42 (dt, *J =* 7.0, 1.2 Hz, 1H), 7.96 (s, 1H), 7.74–7.67 (m, 4H), 7.54 (d, *J =* 8.3 Hz, 1H), 7.45 (ddt, *J =* 9.2, 7.5, 2.4 Hz, 4H), 7.38 (ddd, *J =* 9.1, 6.7, 1.3 Hz, 1H), 6.99 (td, *J =* 6.8, 1.2 Hz, 1H), 6.15 (q, *J =* 7.1 Hz, 1H), 1.97 (d, *J =* 7.1 Hz, 3H).^13^C NMR (101 MHz, DMSO) δ 168.06, 144.94, 142.43, 140.11, 137.69, 136.14, 132.97, 132.88, 130.24, 129.51, 128.71, 126.87, 126.27, 125.32, 124.66, 117.10, 113.32, 111.63, 59.04, 21.07. HRMS: calcd for [M + H], 477.09919; found, 477.09891.

#### 2-Chloro-5-((4-(2-(pyridine-4-yl)­imidazo­[1,2-*a*]­pyridine-3-yl)-1*H*-1,2,3-triazol-1-yl)­methyl)­benzamide (**3**)

The title compound was prepared according to General Procedure IV (Scheme 1). Mobile phase: water/CH_3_CN with 0.1% formic acid (0–70%). Yield: 99.1 mg (63%). ^1^H NMR (401 MHz, MeOD) δ 8.34 (dt, *J =* 7.0, 1.1 Hz, 1H), 8.31 (s, 1H), 7.55 (dt, *J =* 9.1, 1.1 Hz, 3H), 7.48 (d, *J =* 2.0 Hz, 1H), 7.45–7.38 (m, 2H), 7.35 (ddd, *J =* 9.1, 6.8, 1.2 Hz, 1H), 6.91 (td, *J =* 6.9, 1.2 Hz, 1H), 5.70 (s, 2H), 4.84 (s, 4H). ^13^C NMR (101 MHz, MeOD) δ 171.39, 150.19, 147.04, 143.52, 141.71, 137.91, 137.29, 135.88, 131.99, 131.80, 131.74, 129.62, 128.58, 127.00, 126.47, 117.75, 115.04, 114.52, 54.02, 49.85. LC-MS: calcd for [M + H], 430.130, found, 430.118.

#### 2-Chloro-5-((4-(2-(4-cyclopropylphenyl)­imidazo­[1,2-*a*]­pyridin-3-yl)-1*H*-1,2,3-triazol-1-yl)­methyl)­benzamide (**4**)

The title compound was prepared according to General Procedure IV (Scheme 1). Mobile phase petrolether/EtOAc (40–100%). Yield 200 mg (92%). ^1^H NMR (401 MHz, DMSO-*d*_6_) δ 8.50 (s, 1H), 8.47 (dt, *J =* 7.0, 1.2 Hz, 1H), 8.01–7.92 (m, 1H), 7.71 (s, 1H), 7.67 (dt, *J =* 9.1, 1.2 Hz, 1H), 7.60–7.52 (m, 3H), 7.48 (d, *J =* 2.2 Hz, 1H), 7.43 (dd, *J =* 8.3, 2.3 Hz, 1H), 7.35 (ddd, *J =* 9.1, 6.7, 1.3 Hz, 1H), 7.10–7.05 (m, 2H), 6.97 (td, *J =* 6.8, 1.3 Hz, 1H), 5.76 (s, 2H), 1.93 (tt, *J =* 8.4, 5.1 Hz, 1H), 1.01–0.87 (m, 2H), 0.74–0.66 (m, 2H). ^13^C NMR (101 MHz, DMSO) δ 168.00, 144.86, 143.97, 143.81, 137.72, 136.79, 135.17, 131.08, 130.24, 130.19, 129.66, 128.27, 127.82, 125.84, 125.75, 125.53, 125.24, 116.96, 113.04, 110.88, 52.20, 15.18, 9.84. HRMS: calcd for [M + H], 469.15381; found, 469.15344.

#### 2-Chloro-5-((4-(2-(4-chlorophenyl)-8-fluoroimidazo­[1,2-*a*]­pyridin-3-yl)-1*H*-1,2,3-triazol-1-yl)­methyl)­benzamide (**8**)

The title compound was prepared according to General Procedure IV. Mobile phase petrolether/EtOAc (30–100%). Yield 105 mg (89%). ^1^H NMR (401 MHz, DMSO-*d*_6_) δ 8.59 (s, 1H), 8.35 (dd, *J =* 6.9, 0.9 Hz, 1H), 7.93 (s, 1H), 7.74–7.69 (m, 2H), 7.69–7.67 (m, 1H), 7.54 (d, *J =* 8.2 Hz, 1H), 7.49–7.45 (m, 3H), 7.43 (dd, *J =* 8.2, 2.3 Hz, 1H), 7.31 (ddd, *J =* 11.0, 7.7, 0.9 Hz, 1H), 6.98 (ddd, *J =* 7.7, 6.9, 4.8 Hz, 1H), 5.76 (s, 2H). ^13^C NMR (101 MHz, DMSO-*d*_6_) δ 168.25, 150.90 (d, *J =* 249.7 Hz), 142.85, 137.98, 137.64 (d, *J =* 28.8 Hz), 136.22 (d, *J =* 1.3 Hz), 135.31, 133.50, 132.72, 131.94 (d, *J =* 9.8 Hz), 130.53, 129.99, 129.94, 129.12, 128.62, 126.38, 122.50 (d, *J =* 4.9 Hz), 113.70, 112.83 (d, *J =* 6.9 Hz), 109.32 (d, *J =* 15.9 Hz), 52.54. HRMS: calcd for [M + H], 481.07412; found, 481.07324.

#### 2-Chloro-5-((4-(2-(4-chlorophenyl)-7-fluoroimidazo­[1,2-*a*]­pyridin-3-yl)-1*H*-1,2,3-triazol-1-yl)­methyl)­benzamide (**9**)

The title compound was prepared according to General Procedure IV. Mobile phase petrolether/EtOAc (30–100%). Yield 75 mg (83%). ^1^H NMR (401 MHz, DMSO-*d*_6_) δ 8.58–8.51 (m, 2H), 7.92 (s, 1H), 7.72–7.65 (m, 3H), 7.58 (ddd, *J =* 9.9, 2.7, 0.8 Hz, 1H), 7.53 (s, 1H), 7.49–7.38 (m, 4H), 7.05 (td, *J =* 7.6, 2.7 Hz, 1H), 5.75 (s, 2H). ^13^C NMR (101 MHz, DMSO-*d*_6_) δ 167.96, 160.47 (d, *J =* 249.8 Hz), 145.15 (d, *J =* 14.2 Hz), 143.16, 137.68, 136.07, 135.04, 133.02, 132.72, 131.66 (d, *J =* 9.6 Hz), 130.24, 129.63, 129.53, 128.80, 128.32, 127.69 (d, *J =* 11.2 Hz), 125.88, 111.61 (d, *J =* 1.5 Hz), 105.38 (d, *J =* 29.4 Hz), 100.73 (d, *J =* 23.7 Hz), 52.23. HRMS: calcd for [M + H], 481.07412; found, 481.07370.

#### 2-Chloro-5-((4-(2-(4-chlorophenyl)-6-fluoroimidazo­[1,2-*a*]­pyridin-3-yl)-1*H*-1,2,3-triazol-1-yl)­methyl)­benzamide (**10**)

The title compound was prepared according to General Procedure IV. Mobile phase petrolether/EtOAc (30–100%). Yield 63 mg (84%). ^1^H NMR (401 MHz, DMSO-*d*_6_) δ 8.65 (dd, *J =* 4.8, 2.4 Hz, 1H), 8.57 (s, 1H), 7.92 (s, 1H), 7.78 (dd, *J =* 9.8, 5.2 Hz, 1H), 7.69 (dd, *J =* 7.9, 5.8 Hz, 3H), 7.54 (d, *J =* 8.2 Hz, 1H), 7.49–7.43 (m, 4H), 5.75 (s, 2H). ^13^C NMR (101 MHz, DMSO-*d*_6_) δ 167.98, 153.23 (d, *J =* 234.3 Hz), 143.62 (d, *J =* 2.2 Hz), 142.77, 137.67, 135.98, 135.01, 133.06, 132.77, 131.66 (d, *J =* 9.8 Hz), 130.27 (d, *J =* 8.0 Hz), 129.63, 128.80, 128.38, 125.79, 118.09 (d, *J =* 3.6 Hz), 117.92 (d, *J =* 12.9 Hz), 113.25 (d, *J =* 2.0 Hz), 112.30 (d, *J =* 42.5 Hz), 52.25. HRMS: calcd for [M + H], 481.07412; found, 481.07346.

#### 2-Chloro-5-((4-(2-(4-chlorophenyl)-6,8-difluoroimidazo­[1,2-*a*]­pyridin-3-yl)-1*H*-1,2,3-triazol-1-yl)­methyl)-benzamide (**11**)

The title compound was prepared according to General Procedure IV. Mobile phase petrolether/EtOAc (40–100%). Yield 79 mg (88%). ^1^H NMR (401 MHz, DMSO-*d*_6_) δ 8.60 (s, 1H), 8.59 (s, 1H), 7.91 (s, 1H), 7.74–7.65 (m, 4H), 7.54 (d, *J =* 8.2 Hz, 1H), 7.51–7.40 (m, 4H), 5.75 (s, 2H). ^13^C NMR (101 MHz, DMSO-*d*_6_) δ 167.95, 151.91 (dd, *J =* 234.7 Hz, *J =* 11.2 Hz), 149.90 (dd, *J =* 253.3 Hz, *J =* 14.6 Hz), 143.36 (d, *J =* 2.0 Hz), 137.67, 135.47, 135.16, 134.93, 133.35, 132.22, 130.32, 130.21, 129.68, 128.87, 128.39, 126.11, 115.20–114.94 (m), 109.62 (dd, *J =* 43.0, 6.1 Hz), 103.01 (dd, *J =* 30.1, 20.3 Hz), 52.27. HRMS: calcd for [M + H], 499.06470; found, 499.06396.

#### 2-Chloro-5-((4-(2-(4-chlorophenyl)-6,8-difluoroimidazo­[1,2-*a*]­pyridin-3-yl)-1*H*-1,2,3-triazol-1-yl)­methyl)-*N*-methylbenzamide (**12**)

2-(4-Chlorophenyl)-3-ethynyl-6,8-difluoroimidazo­[1,2-*a*]­pyridine (120 mg, 0.415 mmol), 5-(azidomethyl)-2-chloro-*N*-methylbenzamide (103 mg, 0.457 mmol, 1.1 equiv), CuI (20 mg, 0.104 mmol, 0.25 equiv) and TEA (173 μL, 1.247 mmol, 3 equiv) were stirred vigorously in CH_3_CN (10 mL) at RT overnight. The crude product was purified via flash column chromatography (silica gel, cyclohexane/EtOAc 60–10%). Yield 96 mg (45%). ^1^H NMR (400 MHz, DMSO) δ 8.60 (s, 1H), 8.57 (dt, *J =* 4.5, 1.3 Hz, 1H), 8.39 (q, *J =* 4.6 Hz, 1H), 7.73–7.68 (m, 2H), 7.55 (d, *J =* 8.2 Hz, 1H), 7.46 (m, 4H), 7.43 (d, *J =* 2.2 Hz, 1H), 5.74 (s, 2H), 2.76 (d, *J =* 4.6 Hz). *13*C NMR (101 MHz, DMSO) δ 166.35, 152.80 (d, *J =* 11.8 Hz), 150.99 (d, *J =* 14.6 Hz), 150.47 (d, *J =* 11.2 Hz), 148.47 (d, *J =* 14.8 Hz), 143.19 (d, *J =* 1.9 Hz), 137.40, 135.29, 134.99, 134.84, 133.16, 132.04, 130.27, 130.01, 129.68, 129.48, 128.66, 128.33, 125.99, 109.62 (d, *J =* 6.3 Hz), 52.07, 25.93. ^19^F NMR (376 MHz, DMSO) δ −128.03 (dd, *J =* 11.0, 3.8 Hz), −137.76 (dt, *J =* 8.9, 4.1 Hz). HRMS: calcd for [M + H], 513.08035; found, 513.08013.

#### Ethyl 2-(4-chlorophenyl)­imidazo­[1,2-*a*]­pyridine-3-carboxylate (Intermediate Compound) (**18**)

2-Aminopyridine (1.7 g, 3 equiv) was dissolved in CH_3_CN and ethyl 3-(4-chlorophenyl)-3-oxopropanoate (1,36 g, 1 equiv) was added as a solution in CH_3_CN followed by an addition of CBr_4_ (2 equiv). A reaction mixture was stirred at 80 °C overnight. After the completion of the reaction, the mixture was evaporated to a minimal volume, diluted with water and extracted with EtOAc. Combined organic phases were dried over sodium sulfate and purified by flash column chromatography, mobile phase hexane/EtOAc (20–50%). Yield 1.61g (89%). ^1^H NMR (401 MHz, DMSO) δ 9.29 (dt, *J* = 7.0, 1.2 Hz, 1H), 7.82–7.75 (m, 3H), 7.58 (ddd, *J* = 9.0, 6.9, 1.3 Hz, 1H), 7.52–7.47 (m, 2H), 7.23 (td, *J* = 6.9, 1.3 Hz, 1H), 4.25 (q, *J* = 7.1 Hz, 2H), 1.18 (t, *J* = 7.1 Hz, 3H). ^13^C NMR (101 MHz, DMSO) δ 160.24, 151.11, 146.59, 133.61, 133.19, 132.04, 128.88, 128.22, 127.72, 117.34, 114.98, 111.61, 60.53, 13.95.

#### 2-(4-Chlorophenyl)­imidazo­[1,2-*a*]­pyridine-3-carbohydrazide (Intermediate Compound) (**19**)

Ester derivative **18** was dissolved in absolute EtOH and N_2_H_4_.H_2_O (10 equiv) was added. The reaction mixture was refluxed overnight. The reaction mixture was cooled to r.t. and the formed precipitate was filtered, washed with EtOH and dried. Yield: 412 mg (87%). ^1^H NMR (401 MHz, DMSO-*d*_6_) δ 9.70 (s, 1H), 8.57 (d, *J =* 7.0 Hz, 1H), 7.92–7.77 (m, 2H), 7.66 (d, *J =* 9.1 Hz, 1H), 7.56–7.43 (m, 2H), 7.40 (t, *J =* 8.0 Hz, 1H), 7.04 (t, *J =* 6.9 Hz, 1H), 4.67 (s, 2H). ^13^C NMR (101 MHz, DMSO) δ 160.38, 144.68, 143.00, 132.96, 132.64, 129.97, 128.57, 126.85, 126.37, 117.04, 115.62, 113.53. HRMS: calcd for [M + H], 287.06942; found, 287.06950.

#### General Procedure V. HATU Acylation

Hydrazide derivative was dissolved with dry DMF and phenylacetic acid derivative (1 equiv) was added. The reaction mixture was degassed and refilled with argon. HATU (1.2 equiv) was added followed by an addition of DIPEA (1.2 equiv) (Scheme 2). The reaction mixture was stirred at r.t. and monitored by UPLC. After the completion of the reaction, the solution was evaporated to a minimal volume and purified by reverse-phase flash column chromatography.

#### 2-Chloro-5-(2-(2-(2-(4-chlorophenyl)­imidazo­[1,2-*a*]­pyridine-3-carbonyl)­hydrazineyl)-2-oxoethyl)-*N*-methylbenzamide (Intermediate Compound) (**20**)

The title compound was prepared according to General Procedure V. Mobile phase H_2_O/CH_3_CN (20–80%). Yield: 312 mg (86%). ^1^H NMR (401 MHz, DMSO-*d*_6_) δ 10.50 (s, 1H), 10.46 (s, 0H), 8.85–8.78 (m, 1H), 8.36 (q, *J =* 4.6 Hz, 1H), 8.04–7.97 (m, 2H), 7.75–7.67 (m, 1H), 7.52–7.36 (m, 7H), 7.10 (td, *J =* 6.9, 1.2 Hz, 1H), 3.62 (s, 2H), 2.75 (d, *J =* 4.6 Hz, 3H). ^13^C NMR (101 MHz, DMSO) δ 169.39, 166.80, 160.29, 144.98, 143.94, 137.09, 134.74, 133.27, 132.26, 131.57, 130.14, 129.68, 129.64, 128.58, 128.30, 127.29, 126.68, 117.14, 114.67, 113.86, 26.16. HRMS: calcd for [M + Na], 496.09377; found, 496.09422.

#### 2-Chloro-5-(2-(2-(2-(4-chlorophenyl)­imidazo­[1,2-*a*]­pyridine-3-carbonyl)­hydrazineyl)-2-oxoethyl)-*N*,*N*-dimethylbenzamide (Intermediate Compound) (**21**)

The title compound was prepared according to General Procedure V. Mobile phase H_2_O/CH_3_CN (10–70%). Yield: 290 mg (83%). ^1^H NMR (401 MHz, DMSO-*d*_6_) δ 10.54–10.48 (m, 1H), 10.46–10.41 (m, 1H), 8.81 (dt, *J =* 6.9, 1.3 Hz, 1H), 8.04–7.96 (m, 2H), 7.72–7.66 (m, 1H), 7.52–7.46 (m, 4H), 7.42 (td, *J =* 9.2, 8.3, 1.8 Hz, 2H), 7.32 (d, *J =* 2.1 Hz, 1H), 7.11 (td, *J =* 6.9, 1.2 Hz, 1H), 3.64 (s, 2H), 3.01 (s, 3H), 2.79 (s, 3H). ^13^C NMR (101 MHz, DMSO) δ 169.40, 167.10, 160.27, 144.95, 143.91, 136.29, 135.35, 133.31, 132.19, 131.26, 130.18, 129.41, 128.64, 128.57, 127.55, 127.37, 126.70, 117.09, 114.66, 113.92, 48.79, 37.71, 34.20. HRMS: calcd for [M + H], 510.10942; found, 510.10991.

#### 2-Chloro-5-((5-(2-(4-chlorophenyl)­imidazo­[1,2-*a*]­pyridin-3-yl)-1,3,4-oxadiazol-2-yl)­methyl)-*N*-methylbenzamide (**5**)

Hydrazide intermediate **20** was dissolved in dry DCM and tosyl chloride (1.5 equiv) was added followed by TEA (3 equiv) at 0 °C. The reaction mixture was stirred at r.t. overnight. The mixture was diluted with water, extracted with EtOAc and the organic phase was washed with saturated NaHCO_3_ solution. The organic phase was dried over sodium sulfate. Product was isolated by flash column chromatography, mobile phase: hexane/EtOAc (30–100%). Yield 16 mg (6%).^1^H NMR (401 MHz, DMSO-*d*_6_) δ 9.34 (dt, *J =* 6.9, 1.2 Hz, 1H), 8.35 (q, *J =* 4.5 Hz, 1H), 7.85 (dt, *J =* 9.0, 1.2 Hz, 1H), 7.81–7.76 (m, 2H), 7.61 (ddd, *J =* 9.0, 6.9, 1.3 Hz, 1H), 7.49–7.43 (m, 3H), 7.40–7.33 (m, 2H), 7.30 (td, *J =* 6.9, 1.3 Hz, 1H), 4.34 (s, 2H), 2.75 (d, *J =* 4.6 Hz, 3H). ^13^C NMR (101 MHz, DMSO) δ 166.56, 163.71, 157.77, 147.36, 146.56, 137.36, 133.96, 133.63, 132.00, 131.53, 131.16, 129.96, 129.50, 129.01, 128.53, 128.37, 127.79, 117.40, 115.00, 106.35, 29.84, 26.14. HRMS: calcd for [M + Na], 500.06515; found, 500.06550.

#### 2-Chloro-5-((5-(2-(4-chlorophenyl)­imidazo­[1,2-*a*]­pyridin-3-yl)-1,3,4-oxadiazol-2-yl)­methyl)-*N*-methylbenzothioamide (**6**)

A round-bottom flask was charged with a hydrazide derivative **20**, degassed and refilled with argon. Dry toluene was added and the mixture was degassed once more and refilled with argon. P_2_S_5_ (2 equiv) was added and the mixture was stirred at 100 °C overnight. After cooling to r.t. the mixture was diluted with water and extracted with EtOAc. An organic phase was dried over sodium sulfate and evaporated. A residue was purified by RP-flash column chromatography. Mobile phase H_2_O/MeOH (30–100%). Yield: 35 mg (29%). ^1^H NMR (401 MHz, DMSO-*d*_6_) δ 10.50 (p, *J =* 5.0 Hz, 1H), 9.60–9.37 (m, 1H), 7.80 (dt, *J =* 9.0, 1.2 Hz, 1H), 7.68–7.63 (m, 1H), 7.57 (dq, *J =* 9.5, 3.4, 2.3 Hz, 1H), 7.45–7.39 (m, 1H), 7.33 (d, *J =* 7.4 Hz, 1H), 7.24 (td, *J =* 6.9, 1.3 Hz, 1H), 4.50 (s, 1H), 3.09 (d, *J =* 4.7 Hz, 2H). ^13^C NMR (101 MHz, DMSO) δ 195.66, 166.53, 158.33, 148.12, 145.98, 142.68, 136.41, 134.43, 132.18, 131.47, 130.43, 129.81, 129.25, 129.10, 128.25, 127.56, 127.05, 117.29, 114.84, 112.05, 33.47, 32.60. HRMS: calcd for [M + H], 494.06036; found, 494.06045.

#### 2-Chloro-5-((5-(2-(4-chlorophenyl)­imidazo­[1,2-*a*]­pyridin-3-yl)-1,3,4-thiadiazol-2-yl)­methyl)-*N*,*N*-dimethylbenzamide (**7**)

A round-bottom flask was charged with a hydrazide derivative, degassed and refilled with argon. Dry toluene was added and the mixture was degassed once more and refilled with argon. P_2_S_5_ (2 equiv) was added and the mixture was stirred at 100 °C overnight. After cooling to r.t. the mixture was diluted with water and extracted with EtOAc. An organic phase was dried over sodium sulfate and evaporated. A residue was purified by RP-flash column chromatography. Mobile phase H_2_O/MeOH (30–100%). Yield: 75 mg (36%). ^1^H NMR (401 MHz, DMSO-*d*_6_) δ 9.49 (dt, *J =* 7.0, 1.2 Hz, 1H), 7.81 (dt, *J =* 9.1, 1.2 Hz, 1H), 7.67–7.62 (m, 2H), 7.57 (td, *J =* 8.4, 6.7 Hz, 3H), 7.44 (dd, *J =* 8.5, 5.0 Hz, 1H), 7.34–7.22 (m, 3H), 4.49 (s, 2H), 3.50 (s, 3H), 3.01 (s, 3H). ^13^C NMR (101 MHz, DMSO) δ 166.88, 158.33, 148.17, 145.97, 137.17, 136.65, 134.40, 132.20, 131.49, 131.13, 131.05, 129.82, 129.04, 128.38, 128.32, 128.25, 127.94, 127.59, 117.29, 114.84, 112.08, 37.63, 34.17, 33.67. HRMS: calcd for [M + H], 508.07601; found, 508.07605.

### Synthesis of PROTAC Degraders

#### 2-Azido-1,4-dimethoxybenzene (**27**)

2,5-Dimethoxyaniline (1.0 g, 0.007 mol, 1 equiv) was mixed with conc.HCl (3.0 mL, 5 equiv) at 0 °C, solution of NaNO_2_ (0.45 g, 1 equiv) in H_2_O (6.0 mL) was added dropwise, the mixture was stirred 15 min at 0 °C, followed by addition of hexane 2 mL and NaN_3_ (0.47 g, 1.1 equiv) in H_2_O (2 mL). The mixture was stirred at rt overnight. Then water was added and the mixture extracted with EtOAc/hexane 1:1 mixture. The organic phase was dried over sodium sulfate, evaporated and the crude purified by column chromatography, mobile phase cyclohexane/EtOAc 4:1. Yield 373 mg (32%). Analytical data agrees with the literature. ^1^H NMR (500 MHz, DMSO) δ 6.17 (d, *J* = 9.0 Hz, 1H), 5.88 (dd, *J* = 9.0, 3.0 Hz, 1H), 5.75 (d, *J* = 3.0 Hz, 1H), 2.94 (s, 3H), 2.87 (s, 3H). ^13^C NMR (126 MHz, DMSO) δ 153.89, 146.49, 128.08, 114.24, 110.89, 107.01, 56.80, 55.68.

#### 4-Amino-2-(2,6-dioxopiperidin-3-yl)­isoindoline-1,3-dione (**29**)

4-Fluoroisobenzofuran-1,3-dione **28** (1.0 g, 0.006 mol, 1 equiv) and 3-aminopiperidine-2,6-dione hydrobromide (1.26 g, 1 equiv) were combined in AcOH (8 mL) and NaOAc (0.74 g, 1.5 equiv) was added, the mixture was heated up to reflux overnight. After the completion of the reaction the mixture was evaporated, diluted with water and the suspension filtered. Yield 1.62 g (97%). ^1^H NMR (401 MHz, DMSO) δ 11.15 (s, 1H), 7.95 (ddd, *J* = 8.4, 7.3, 4.5 Hz, 1H), 7.83–7.67 (m, 2H), 5.16 (dd, *J* = 12.9, 5.4 Hz, 1H), 2.89 (ddd, *J* = 17.0, 13.9, 5.5 Hz, 1H), 2.65–2.55 (m, 1H). overlay with DMSO ^13^C NMR (101 MHz, DMSO) δ 172.91, 169.85, 166.29, 166.26, 164.14, 158.28, 155.68, 138.27, 138.19, 133.63, 133.62, 123.27, 123.08, 120.24, 120.20, 117.27, 117.15, 49.26, 31.07, 22.01. HRMS: calcd for [M + Na], 299.04386, found 299.04382

#### 2-(2,6-Dioxopiperidin-3-yl)-4-(prop-2-yn-1-ylamino)­isoindoline-1,3-dione (**30**)

4-Amino-2-(2,6-dioxopiperidin-3-yl)­isoindoline-1,3-dione **29** (0.3 g, 1 equiv) was dissolved in dry DMSO (4.5 mL) and propargyl amine (0.073 g, 1.1 equiv) was added followed by an addition of DIPEA (0.63 mL, 3 equiv) and the mixture was stirred at 90 °C for 1 h, when the TLC indicated completion of the reaction. The mixture was diluted with water and extracted with EtOAc. Organic phase was washed with brine and dried over sodium sulfate. The crude was purified by flash column chromatography. Yield 115 mg of yellow foam (31%). ^1^H NMR (401 MHz, DMSO) δ 11.10 (s, 1H), 7.65 (dd, *J* = 8.5, 7.1 Hz, 1H), 7.21–7.06 (m, 2H), 6.93 (t, *J* = 6.2 Hz, 1H), 5.07 (dd, *J* = 12.8, 5.4 Hz, 1H), 4.17 (dd, *J* = 6.2, 2.4 Hz, 2H), 3.17 (t, *J* = 2.4 Hz, 1H), 2.94–2.83 (m, 1H), 2.65–2.52 (m, 1H), 2.07–1.98 (m, 1H). DMSO overlap ^13^C NMR (101 MHz, DMSO) δ 173.01, 170.25, 168.77, 167.44, 145.37, 136.26, 132.33, 118.15, 111.56, 110.46, 81.09, 73.95, 48.79, 31.70, 31.16, 22.30. HRMS: calcd for [M + H], 312.09788, found 312.09779.

#### *N*-(4-((2-Oxopropyl)­sulfonyl)­phenyl)­acetamide (**32**)

4-Acetamidobenzenesulfonyl chloride (**31**) (2 g) was added to the solution of NaHCO_3_ (1.44 g, 0.24 equiv) and Na_2_SO_3_ (2.37 g, 1.2 equiv) suspended in water (40 mL). The mixture was stirred overnight. Then the reaction was evaporated, diluted with hot EtOH and filtered. The solution was evaporated providing the sodium salt intermediate in quantitative yield. Next, chloroacetone (ml, 1.1 equiv) was added to the solution of intermediate in EtOH (20 mL) and catalytic amount of KI was added. The mixture was stirred at reflux overnight. The mixture was evaporated and the crude was purified by flash column chromatography, mobile phase cyclohexan/EtOAc (30–100%). Yield 1,66 g (76%). ^1^H NMR (401 MHz, DMSO) δ 10.41 (s, 1H), 7.81 (s, 4H), 4.60 (s, 2H), 2.20 (s, 3H), 2.10 (s, 3H). ^13^C NMR (101 MHz, DMSO) δ 197.38, 169.39, 144.38, 132.80, 129.38, 118.71, 66.62, 31.35, 24.37. HRMS: calcd for [M + Na], 278.04575; found, 278.04570.

#### 4-((1-(2,5-Dimethoxyphenyl)-5-methyl-1*H*-1,2,3-triazol-4-yl)­sulfonyl)­aniline (**33**)

Dimroth cyclization: **27** (100 mg, 1 equiv) was dissolved in NaOMe solution (1 M in methanol) (3 mL), degassed at 0 °C and *N*-(4-((2-oxopropyl)­sulfonyl)­phenyl)­acetamide (142 mg, 1 equiv) was added.The mixture was stirred at 60 °C for 8 h and another NaOMe solution (3 mL) was added and the mixture was stirred overnight. The mixture was evaporated, diluted with water and extracted with EtOAc, organic phase was dried over sodium sulfate and evaporated. The crude was purified by flash column chromatography, mobile phase cyclohexan/EtOAc. Yield 116 mg (56%). NMR: ^1^H NMR (401 MHz, DMSO) δ 7.65–7.60 (m, 2H), 7.26 (d, *J* = 9.1 Hz, 1H), 7.22–7.14 (m, 2H), 6.70–6.64 (m, 2H), 6.27 (s, 2H), 3.73 (d, *J* = 5.2 Hz, 6H), 2.32 (s, 3H). ^13^C NMR (101 MHz, DMSO) δ 154.29, 153.24, 147.79, 144.94, 137.33, 129.71, 125.19, 123.34, 118.08, 114.08, 114.07, 113.11, 56.50, 56.05, 8.66. HRMS: calcd for [M + H], 375.11215 found 375.11188

#### 3-(2-(2-Azidoethoxy)­ethoxy)-*N*-(4-((1-(2,5-dimethoxyphenyl)-5-methyl-1*H*-1,2,3-triazol-4-yl)­sulfonyl)­phenyl)­propenamide (**34a**)

Compound **33** (265 mg, 1 equiv) was added to a solution of 3-(2-(2-azidoethoxy)­ethoxy)­propanoyl chloride (1.3 equiv) in dry NMP and stirred at r.t. The mixture was diluted with EtOAc, washed with water and purified by column chromatography hexane/acetone (1:1, 5% TEA). Yield 249 mg (65%) as a partial mixture with **33**. Pure analytical sample for analysis. ^1^H NMR (500 MHz, DMSO) δ 10.45 (s, 1H), 8.08–7.90 (m, 3H), 7.90–7.78 (m, 2H), 7.26 (d, *J* = 9.2 Hz, 1H), 7.24–7.14 (m, 2H), 3.75–3.69 (m, 7H), 3.59–3.55 (m, 2H), 3.53 (d, *J* = 1.6 Hz, 3H), 2.61 (t, *J* = 6.2 Hz, 2H), 2.36 (s, 3H). ^13^C NMR (126 MHz, DMSO) δ 170.67, 153.54, 148.07, 144.66, 143.84, 138.88, 134.55, 129.21, 123.47, 119.56, 118.49, 114.40, 114.37, 70.17, 70.04, 69.70, 66.92, 56.83, 56.35, 50.41, 31.15, 9.01. HRMS: calcd for [M + H], 560,19219; found 560.1913.

#### 3-(2-(2-(2-azidoethoxy)­ethoxy)­ethoxy)-*N*-(4-((1-(2,5-dimethoxyphenyl)-5-methyl-1*H*-1,2,3-triazol-4-yl)­sulfonyl)­phenyl)­propenamide (**34b**)

Compound **33** (108 mg, 1 equiv) was dissolved in dry DMF (2.0 mL), degassed and HATU (120 mg, 1.1 equiv) was added followed by TEA (0.1 mL, 2.7 equiv). Finally, linker 3-(2-(2-(2-azidoethoxy)­ethoxy)­ethoxy)­propanoic acid (71 mg, 1.0 equiv) was added and the mixture stirred at r.t.. The mixture was directly purified by reverse-phase flash column chromatography H_2_O/MeOH (5–80%). Yield 35 mg (20%). ^1^H NMR (401 MHz, MeOD) δ 8.01–7.95 (m, 1H), 7.92–7.82 (m, 1H), 7.23–7.15 (m, 1H), 7.03 (dd, *J* = 2.7, 0.7 Hz, 1H), 3.84 (t, *J* = 6.0 Hz, 1H), 3.78 (s, 2H), 3.77 (s, 2H), 3.67–3.55 (m, 8H), 3.32–3.27 (m, 1H), 2.68 (t, *J* = 6.0 Hz, 1H), 2.44 (s, 2H). ^13^C NMR (101 MHz, MeOD) δ 172.75, 155.18, 149.32, 145.29, 140.23, 136.47, 129.89, 124.79, 120.74, 119.12, 114.89, 114.71, 71.57, 71.52, 71.47, 71.46, 71.44, 71.02, 67.94, 56.93, 56.51, 51.68, 38.70, 9.05. HRMS: calcd for [M + H], 516.16598, found 516.16577.

#### 1-azido-*N*-(4-((1-(2,5-dimethoxyphenyl)-5-methyl-1*H*-1,2,3-triazol-4-yl)­sulfonyl)­phenyl)-3,6,9,12-tetraoxapentadecan-15-amide (**34c**)

Compound **33** (257 mg, 1 equiv) was added to to solution of 1-azido-3,6,9,12-tetraoxapentadecan-15-oyl chloride (1.3 equiv) in dry NMP. The mixture stirred at r.t. The mixture was diluted with EtOAc, washed with water and purified by column chromatography hexane/acetone (2:1). Yield 82 mg (20%). ^1^H NMR (500 MHz, DMSO) δ 10.45 (s, 1H), 8.05–7.94 (m, 2H), 7.92–7.80 (m, 2H), 7.35–7.11 (m, 3H), 3.76–3.68 (m, 8H), 3.61–3.56 (m, 2H), 3.54–3.47 (m, 11H), 3.38 (dd, *J* = 5.6, 4.3 Hz, 2H), 3.32–3.28 (m, 2H), 2.70 (t, *J* = 0.8 Hz, 2H), 2.61 (t, *J* = 6.1 Hz, 2H), 2.37 (s, 3H). ^13^C NMR (126 MHz, DMSO) δ 174.23, 170.70, 153.54, 148.07, 144.66, 143.84, 138.88, 134.56, 129.21, 123.47, 119.56, 118.49, 114.40, 114.38, 70.27, 70.23, 70.18, 70.14, 69.69, 66.88, 56.83, 56.35, 50.45, 48.95, 37.77, 29.47, 9.01. HRMS: calcd for [M + H], 648,24462; found 648,2433.

#### *N*-(4-((1-(2,5-Dimethoxyphenyl)-5-methyl-1*H*-1,2,3-triazol-4-yl)­sulfonyl)­phenyl)-3-(2-(2-(4-(((2-(2,6-dioxopiperidin-3-yl)-1,3-dioxoisoindolin-4-yl)­amino)­methyl)-1*H*-1,2,3-triazol-1-yl)­ethoxy)­ethoxy)­propenamide (**35**)

Compound **34a** (249 mg, 1 equiv) was dissolved in THF/water 1:1 mixture and degassed at 0 °C and refilled with argon. Compound **30** (110 mg, 0.8 equiv) was added together with sodium ascorbate (88 mg, 1 equiv) and CuSO_4_.5H_2_O (10 mg, 0.05 equiv). The mixture was degassed again and stirred at r.t. for 3 h. Completion of the reaction monitored by TLC. After completion of the reaction, the mixture was diluted with EtOAc, washed with water and purified by column chromatography (eluent EtOAc/MeOH (8:1). Yield 237 mg (70%). ^1^H NMR (500 MHz, DMSO) δ 11.10 (s, 1H), 10.43 (s, 1H), 7.97 (d, *J* = 8.7 Hz, 3H), 7.92–7.79 (m, 2H), 7.65–7.51 (m, 1H), 7.31–7.14 (m, 3H), 7.14–7.00 (m, 2H), 5.75 (s, 2H), 5.06 (dd, *J* = 12.8, 5.4 Hz, 1H), 4.59 (d, *J* = 6.1 Hz, 2H), 4.45 (t, *J* = 5.2 Hz, 2H), 3.79–3.74 (m, 2H), 3.73 (d, *J* = 5.2 Hz, 5H), 3.65 (t, *J* = 6.1 Hz, 2H), 3.46 (tt, *J* = 7.8, 3.7 Hz, 4H), 2.95–2.82 (m, 1H), 2.64–2.52 (m, 4H), 2.37 (s, 3H), 2.07–1.99 (m, 1H). ^13^C NMR (126 MHz, DMSO) δ 172.98, 170.38, 170.24, 168.96, 167.45, 153.24, 147.77, 146.01, 144.51, 144.33, 143.55, 138.60, 136.27, 134.27, 132.29, 128.92, 123.42, 123.18, 119.27, 118.17, 117.79, 114.08, 111.10, 109.88, 69.69, 69.63, 68.88, 66.56, 59.94, 56.53, 56.05, 55.08, 49.53, 48.76, 37.78, 37.44, 31.16, 22.33, 8.73. HRMS: calcd for [M + H], 871,2828; found 871.2822.

#### *N*-(4-((1-(2,5-dimethoxyphenyl)-5-methyl-1*H*-1,2,3-triazol-4-yl)­sulfonyl)­phenyl)-3-(2-(2-(2-(4-(((2-(2,6-dioxopiperidin-3-yl)-1,3-dioxoisoindolin-4-yl)­amino)­methyl)-1*H*-1,2,3-triazol-1-yl)­ethoxy)­ethoxy)­ethoxy)­propenamide (**36**)

Compound **34b** (35 mg, 1 equiv) was dissolved in THF/water 1:1 mixture and degassed at 0 °C and refilled with argon. Compound **30** (18.0 mg, 1 equiv) was added together with sodium ascorbate (11 mg, 1 equiv) and CuSO_4_.5H_2_O (0.7 mg, 0.05 equiv). The mixture was degassed again and stirred at r.t. for 3 h. Completion of the reaction monitored by UPLC. After the completion the mixture was evaporated, dissolved in DMSO/H_2_O 1:1 mixture (4 mL) and purified by HPLC (H_2_O/ACN, 10–60%). Yield 39 mg (74%). ^1^H NMR (600 MHz, DMSO) δ 11.10 (s, 1H), 10.45 (s, 1H), 7.99–7.94 (m, 3H), 7.89–7.84 (m, 2H), 7.56 (dd, *J* = 8.5, 7.1 Hz, 1H), 7.26 (d, *J* = 9.2 Hz, 1H), 7.20 (dd, *J* = 9.1, 3.1 Hz, 1H), 7.18–7.15 (m, 2H), 7.04 (d, *J* = 7.0 Hz, 1H), 5.06 (dd, *J* = 12.8, 5.5 Hz, 1H), 4.59 (s, 2H), 4.45 (t, *J* = 5.2 Hz, 2H), 3.76–3.74 (m, 2H), 3.73 (d, *J* = 6.1 Hz, 5H), 3.68 (t, *J* = 6.2 Hz, 2H), 3.48–3.38 (m, 11H), 2.88 (ddd, *J* = 16.9, 13.8, 5.4 Hz, 1H), 2.59 (p, *J* = 4.3 Hz, 3H), 2.36 (s, 3H), 2.08 (s, 1H), 2.02 (ddq, *J* = 11.0, 5.4, 2.8 Hz, 1H). ^13^C NMR (151 MHz, DMSO) δ 172.99, 170.41, 170.24, 168.96, 167.46, 153.24, 147.78, 146.02, 144.53, 144.36, 143.55, 138.61, 136.29, 134.26, 132.29, 128.91, 123.44, 123.18, 119.26, 118.18, 117.80, 114.09, 111.11, 109.86, 69.85, 69.80, 69.77, 69.69, 68.84, 66.56, 56.54, 56.05, 49.55, 48.75, 37.80, 37.45, 31.16, 22.33, 8.73. HRMS: calcd for [M + H], 915.30901, found 915.3087.

#### *N*-(4-((1-(2,5-dimethoxyphenyl)-5-methyl-1*H*-1,2,3-triazol-4-yl)­sulfonyl)­phenyl)-1-(4-(((2-(2,6-dioxopiperidin-3-yl)-1,3-dioxoisoindolin-4-yl)­amino)­methyl)-1*H*-1,2,3-triazol-1-yl)-3,6,9,12-tetraoxapentadecan-15-amide (**37**)

Compound **34c** (82 mg, 1 equiv) was dissolved in THF/water 1:1 mixture and degassed at 0 °C and refilled with argon. Compound **30** (40 mg, 1 equiv) was added together with sodium ascorbate (25 mg, 1 equiv) and CuSO_4_.5H_2_O (0.7 mg, 0.05 equiv). The mixture was degassed again and stirred at r.t. for 3 h. Completion of the reaction monitored by TLC. After completion of the reaction, the mixture was diluted with EtOAc, washed with water and purified by column chromatography (eluent EtOAc/MeOH (8:1). Yield 89 mg (74%). ^1^H NMR (500 MHz, DMSO) δ 11.09 (s, 1H), 10.45 (s, 1H), 8.00–7.94 (m, 3H), 7.88–7.84 (m, 2H), 7.56 (dd, *J* = 8.6, 7.1 Hz, 1H), 7.26 (d, *J* = 9.2 Hz, 1H), 7.23–7.14 (m, 3H), 7.04 (d, *J* = 7.0 Hz, 2H), 5.05 (dd, *J* = 12.8, 5.4 Hz, 1H), 4.59 (d, *J* = 4.3 Hz, 2H), 4.47 (t, *J* = 5.2 Hz, 2H), 3.76 (t, *J* = 5.2 Hz, 2H), 3.72 (d, *J* = 6.0 Hz, 6H), 3.68 (t, *J* = 6.1 Hz, 2H), 3.51–3.42 (m, 8H), 3.39 (tt, *J* = 4.4, 1.6 Hz, 6H), 3.32–3.27 (m, 3H), 2.36 (s, 3H), 2.17 (t, *J* = 8.1 Hz, 2H), 1.96–1.85 (m, 2H). ^13^C NMR (126 MHz, DMSO) δ 173.94, 172.97, 170.39, 170.21, 168.93, 167.44, 153.23, 147.76, 146.00, 144.35, 143.53, 138.58, 136.29, 134.24, 132.27, 128.90, 123.47, 123.16, 119.25, 118.18, 117.80, 114.09, 114.07, 111.09, 109.84, 69.89, 69.86, 69.83, 69.79, 69.74, 69.67, 68.84, 66.55, 56.53, 56.05, 49.54, 48.73, 48.65, 37.78, 37.43, 31.14, 30.27, 29.17, 17.39, 8.71. HRMS: calcd for [M + H], 959,33523; found 959,3353.

Compounds MI891 (**9**) and MI1013 (**36**), as well as other compounds in Table 1 and PROTAC (**36**) derivatives, used for biological experiments, were >95% pure by HPLC analysis (Figure S13A,B).

### Cell Culture

Human hepatocellular carcinoma HepG2 (RRID: CVCL_0027), human kidney epithelial cell line HK2 (CRL-2190, ATCC), and monkey fibroblast-like COS-1 (RRID: CVCL_0223) cell lines were cultured as previously described, with experiments conducted between passages 5 and 25. LS174T (RRID: CVCL_1384), an epithelial colon adenocarcinoma cell line (Merck Life Science), with functional PXR nuclear receptor expression, was also cultured as previously described.^25^

HepaRG cells (Sigma-Aldrich/Merck) were cultivated and differentiated on 12-well plates. Seeded at 26,600 cells/cm^2^, they were maintained in William’s medium with supplements. After 14 days, differentiation into hepatocyte-like cells was induced using 1.5% DMSO for another 14 days. Differentiated HepaRG cells were treated with test compounds for 48 h as follows: control (DMSO vehicle 0.1% (v/v)), rifampicin, propiconazole, and SPA70 (5 μM), MI891 (5 μM), and combinations for an antagonist mode (rifampicin 5 μM + MI891 5 μM, rifampicin 5 μM + SPA70 5 μM or rifampicin 5 μM + MI1013 2 μM). In dose-dependent experiments, HepaRG were treated with MI891 in the range from 1 up to 20 μM alone or with rifampicin (5 μM) or CITCO (1 μM) for 48 h.

### Primary Human Hepatocytes (PHH)

Cryopreserved human hepatocytes from three donors were purchased from BioIVT (Westbury, New York, USA). The characteristics of the donors were as follows: donor 1 - female, 30 years, Hispanic, donor 2 - female, 78 years, Caucasian, donor 3 - male, 58 years, African American. PHHs were seeded in PHH medium supplemented with 10% fetal bovine serum at a density of 350,000 cells per well of rat tail collagen type I-coated 24-well plates (catalog number 354408, Corning, New York, USA).^44^ Cultivation medium was replaced with serum-free PHH medium 2 h later. The next day, 2D PHHs were treated with test compounds for 24 h as follows: control (DMSO vehicle 0.1% (v/v)), rifampicin and SPA70 (10 μM), MI891 (5 μM), and combinations for an antagonist mode (rifampicin 10 μM + MI891 5 μM or rifampicin 10 μM + SPA70 10 μM). Rifampicin, SPA70, and dimethyl sulfoxide (DMSO) were all purchased from Sigma-Aldrich (St. Louis, Missouri, USA, now part of Merck).

### Plasmids

The *CYP3A4* gene promoter luciferase reporter construct (p3A4-luc) bears a distal XREM (−7836/–7208) and a proximal promoter sequence (prPXRE, −362/+53) from the CYP3A4 gene promoter region. The PXR activity was assessed as the PXR-dependent transcriptional activation using the construct. The human PXR expression vector (variant 1, NM_003889.3) as well as vectors for mouse, rat, and monkey orthologues were described in our recent paper (39).^44^ The control vector pRL-TK (Promega) constitutively expresses *Renilla* luciferase. CYP2B6-luc reporter plasmid (originally entitled as B-1.6k/PB/XREM) was kindly donated by Dr. Hongbing Wang (University of Maryland School of Pharmacy, Baltimore, MD, USA) and was used in assays with wild-type CAR (NM_005122.4, transcript variant 3) and CAR3 (XM_005245697.4, transcript variant X4) variants and mouse CAR variant as described before.^25^ Rhesus monkey, mouse, and rat PXR expression vectors (100 ng/well each) were also obtained from GenScript. The pSG5-RXRα expression construct was kindly provided by Dr. C. Carlberg (University of Kuopio, Kuopio, Finland). Transient transfection experiments followed published protocols used in our laboratory and described before.^25,44^

The CAR LBD assembly assay used constructs encoding helices 3–12 and helix 1 of human CAR LBD as described before.^25^ GL4.31­[luc2P/GAL4UAS/Hygro] (pGAL4 UAS-luc) luciferase reporter vector and pRL-TK *Renilla* expression vector (Promega) were used in the assay in HepG2 cells transfected using Lipofectamine 3000.

Additionally, the GAL4-PXR-LBD triple mutants (S247W/C284W/S208W) construct and wild-type GAL4-wtPXR vector were used for PXR LBD mutant functional two-hybrid assay as we described before.^45^ In the mammalian two-hybrid assay, HepG2 cells were cotransfected with a GAL4-hPXR LBD construct and pSCR1-VP16 construct. *Renilla* luciferase vector was used for the normalization of firefly luciferase activity to *Renilla* luciferase activity.

For the farnesoid X receptor (FXR) luciferase gene reporter assay, the full-length expression vector was paired with pFXRE-luc luciferase construct. The p1A1-luc construct was used in the aryl hydrocarbon receptor (AhR) assays with pRL-TK for normalization. Gal4-LXR-α/β, and Gal4-PPAR-γ constructs were used with the pGAL4 UAS-luc construct. For the VDR assay, expression vector pSG5-hVDR together with pVDRE-luc constructs was used. Firefly and Renilla luciferase activities were analyzed using a Dual reporter assay (Promega). For the nuclear receptor assays, prototype ligands were used at the following concentrations: Calcitriol for VDR (100 nM), GW3965 for LXRα and LXRβ (10 μM), 6-OCA for FXR (1 μM), fenofibrate for PPARα (10 μM), estradiol for ERα and β (10 μM), rosiglitazone for PPARγ (10 μM), 3-MC for AhR (5 μM), and TCPOBOP for mCAR (10 μM). Detailed descriptions and protocols are detailed in our recent publication.^25,44^

### Agonist, Antagonist, and Inverse Agonist Gene Reporter and Two-Hybrid Luciferase Assays

Luciferase reporter assays were conducted to evaluate interactions with PXR or CAR and their variants in HepG2, or COS-1. Cells were seeded at a density of 40,000 cells/cm^2^ in 48-well plates (ThermoFisher Scientific) and maintained at 37 °C in a humidified atmosphere with 5% CO_2_. The cells were cultured in DMEM medium (Merck) supplemented with 10% fetal bovine serum. Following seeding, HepG2 cells were transfected with 150 ng/well of a luciferase reporter construct and 100 ng/well of a nuclear receptor expression vector using Lipofectamine 3000 (ThermoFisher Scientific) and with pRL-TK (30 ng/well) serving as the normalization control. After 24 h, cells were treated with test compounds for 24 h before cell lysis and subsequent firefly and *Renilla* luciferase activity analysis using the Dual-Glo Luciferase Assay System (Promega). Data were normalized to the DMSO control and presented as fold change in *Renilla*-normalized firefly luciferase activity.^25,44^

In agonistic assays, vehicle (DMSO, 0.1%) served as the control (0% activation), while rifampicin (10 μM) activity was the positive control (set to be 100% activation). For antagonistic assays, rifampicin alone (10 μM) was set to be 0% inhibition), while the activity of vehicle (DMSO, 0.1%) was set to 100% inhibition. Inverse agonist assays used SPA70 (10 μM) as the positive control (100% inverse agonistic activity). The activities of test compounds were normalized to the respective controls to determine % activation or inhibition. Results are expressed as mean ± SD from three or more independent biological replicates, each performed in technical triplicates.^12,15,25^

The half-maximal effective concentration (EC_50_) for activating PXR was determined using at least six or eight data points, ranging from 1 nM to 30 μM. The half-maximal effective concentration (IC_50_) for inhibiting PXR (either in antagonistic or inverse agonistic modes) was determined using at least six data points, ranging from 1 nM to 30 μM.

### RT-qPCR

RT-qPCR was utilized to investigate gene expression in HepaRG and PHH. Total RNA was extracted from these cells using the phenol–chloroform method. The concentration of the isolated RNA was then measured using a Nanodrop 1000 spectro-photometer (Thermo Fisher Scientific).

Following RNA isolation, reverse transcription was performed to convert RNA into complementary DNA (cDNA). This step was carried out using the High-capacity cDNA reverse transcription kit (Cat. No. 4374966, ThermoFisher Scientific). The resulting cDNA served as the template for subsequent qPCR reactions.

The qPCR reactions were conducted on the QuantStudio 6 Flex Real-Time PCR System (ThermoFisher Scientific, Waltham, Mas-sachusetts). For these reactions, TaqMan Universal master mix II (Cat. No. 4440047, ThermoFisher Scientific) and TaqMan assays (FAM) utilized in this study are detailed in the Supporting Information. To quantify gene expression, the cycle threshold (Ct) values of the target genes were normalized to the Ct values of reference genes HPRT1 or GAPDH (HepaRG) or TBP (PHH). The normalized values were then expressed as fold changes (2−ΔΔCt) relative to the DMSO control at the specified time points, with the control set as 1.^44^

### TR-FRET CAR Coactivator Binding Assay

We utilized the LanthaScreen TR-FRET CAR Coactivator Binding Assay Kit, goat (Thermo Fisher Scientific, Cat. No. PV4836), which includes GST-tagged human CAR LBD and a fluorescein-labeled PGC1α coactivator peptide. The assay was performed with slight modifications to the manufacturer’s protocol, as previously reported. The half-maximal effective concentration (EC_50_) for activating CAR LBD was determined using at least six data points, ranging from 10 pM to 10 μM.^25^ The interaction with the PXR ligand-binding domain (LBD) was assessed using the LanthaScreen TR-FRET PXR Competitive Binding Assay (Thermo Fisher Scientific, PV4839), as previously described.^26^ This assay quantifies the ability of test compounds to displace a fluorescent PXR ligand from the receptor. Fluorescence readings were taken on a Synergy 2 Multi-Mode Microplate Reader (BioTek, Winooski, VT). The half-maximal inhibitory concentration (IC_50_) for PXR LBD was calculated from at least eight data points using GraphPad Prism software.

### Human and Mouse Plasma Protein Binding, Metabolic Stability in Human or Mouse Liver Microsomes, and Human Liver S9 Fraction

The protocols and reagents for the plasma protein binding assay and metabolic stability testing in human and mouse liver microsomes are detailed in our previous report.^25^ Briefly, metabolic stability of compounds was assessed at a single concentration (5 μM) at t = 0, 10, 30, and at t = 45 min. The stability of compounds was tested in human and mouse microsomes (ThermoFisher Scientific). Compounds were tested in triplicate with or without NADPH wells as a negative control for cytochrome P450 metabolism. In addition to hepatic metabolism, compounds were also subjected to degradation/modification by enzymes in human (VWR International) and mouse (Biomedica, CZ) plasma for *t* = 0, 30, 60, and at *t* = 120 min. Each assay included a substrate with known activity as a positive control. Time points were analyzed by LC/MS/MS. The peak area for the parent compound is compared to the time zero sample in order to assess the amount of remaining compound.

### Cytotoxicity Analysis

For the cytotoxicity assays involving HepG2, COS-1, and HK-2 cell lines, serial dilutions of MI891 or MI1013 were added to white 96-well tissue culture-treated plates containing these cell lines. After incubating the plates for 24 h at 37 °C, cytotoxicity was assessed using the CellTiter-Glo luminescent cell viability assay (Promega). The luminescence signal was measured with a Synergy2 plate reader (BioTek, Winooski, Vermont, USA) and presented as a relative change compared to the vehicle (DMSO, 0.1%) control, which was set at 100%.

### Quantitative Proteomics

MOLT4 cells were treated with DMSO or 5 μM of MI1013 or pomalidomide (positive control) for 5 h, and then cells were harvested by centrifugation at 4 °C before being snap-frozen in liquid nitrogen. Cells were lysed by the addition of lysis buffer (8 M Urea, 50 mM NaCl, 50 mM 4-(2-hydroxyethyl)-1-piperazineethanesulfonic acid (EPPS) pH 8.5, Protease and Phosphatase inhibitors) and homogenization by bead beating (BioSpec) for three repeats of 30 s at 2400. Bradford assay was used to determine the final protein concentration in the clarified cell lysate. 50 μg of protein for each sample was reduced, alkylated, and precipitated using methanol/chloroform as previously described and the resulting washed precipitated protein was allowed to air-dry. Precipitated protein was resuspended in 4 M Urea, 50 mM HEPES pH 7.4, followed by dilution to <1 M urea with the addition of 200 mM EPPS, pH 8. Proteins were digested overnight at 37 °C with LysC (1:50; enzyme:protein) and trypsin (1:50; enzyme:protein). Sample digests were acidified with formic acid to a pH of 2–3 prior to desalting using C18 solid-phase extraction plates (SOLA, Thermo Fisher Scientific). Desalted peptides were dried in a vacuum-centrifuge and reconstituted in 0.1% formic acid for LC-MS analysis.^46^

Data were collected using a TimsTOF Pro2 (Bruker Daltonics, Bremen, Germany) coupled to a nanoElute LC pump (Bruker Daltonics, Bremen, Germany) via a CaptiveSpray nanoelectrospray source. Peptides were separated on a reversed-phase C18 column (25 cm × 75 μm ID, 1.6 μM, IonOpticks, Australia) containing an integrated captive spray emitter. Peptides were separated using a 50 min gradient of 2–30% buffer B (acetonitrile in 0.1% formic acid) with a flow rate of 250 nL/min and a column temperature maintained at 50 °C.

DDA was performed in Parallel Accumulation-Serial Fragmentation (PASEF) mode to determine effective ion mobility windows for downstream diaPASEF data collection.^47^ The ddaPASEF parameters included: 100% duty cycle using accumulation and ramp times of 50 ms each, 1 TIMS-MS scan and 10 PASEF ramps per acquisition cycle. The TIMS-MS survey scan was acquired between 100–1700 *m*/*z* and 1/*k*_0_ of 0.7–1.3 V s/cm^2^. Precursors with 1–5 charges were selected and those that reached an intensity threshold of 20,000 arbitrary units were actively excluded for 0.4 min. The quadrupole isolation width was set to 2 *m*/*z* for *m*/*z* < 700 and 3 *m*/*z* for *m*/*z* > 800, with the *m*/*z* between 700 and 800 *m*/*z* being interpolated linearly. The TIMS elution voltages were calibrated linearly with three points (Agilent ESI-L Tuning Mix Ions; 622, 922, 1,222 *m*/*z*) to determine the reduced ion mobility coefficients (1/*k*_0_). To perform diaPASEF, the precursor distribution in the DDA *m*/*z*-ion mobility plane was used to design an acquisition scheme for DIA data collection, which included two windows in each 50 ms diaPASEF scan. Data were acquired using 16 of these 25 Da precursor double window scans (creating 32 windows), which covered the diagonal scan line for doubly and triply charged precursors, with singly charged precursors able to be excluded by their position in the *m*/*z*-ion mobility plane. These precursor isolation windows were defined between 400–1200 *m*/*z* and 1/k0 of 0.7–1.3 V s/cm^2^.

### LC-MS Data Analysis

The diaPASEF raw file processing and controlling peptide and protein level false discovery rates, assembling proteins from peptides, and protein quantification from peptides were performed using library-free analysis in DIA-NN 1.8.^48^ Library free mode performs an in-silico digestion of a given protein sequence database alongside deep learning-based predictions to extract the DIA precursor data into a collection of MS2 spectra. The search results are then used to generate a spectral library, which is then employed for the targeted analysis of the DIA data searched against a SwissProt human database (January 2021). Database search criteria largely followed the default settings for directDIA including tryptic with two missed cleavages, carbamidomethylation of cysteine as a fixed modification, and oxidation of methionine as a variable modification and precursor Q-value (FDR) cutoff of 0.01. Precursor quantification strategy was set to Robust LC (high accuracy) with RT-dependent cross-run normalization.

Proteins with low sum of abundance (<2000 × no. of treatments) were excluded from further analysis and proteins with missing values were imputed by random selection from a Gaussian distribution either with a mean of the nonmissing values for that treatment group or with a mean equal to the median of the background (in cases when all values for a treatment group are missing). Protein abundances were scaled using in-house scripts within the R framework (R Development Core Team, 2014), and the resulting data were filtered to include only proteins with a minimum of 2 counts in at least four replicates of each independent comparison between treatment samples and the DMSO control. Significant changes comparing the relative protein abundance of these treatments to DMSO control comparisons were assessed by moderated *t* test as implemented in the limma package within the R framework.^49^

### Microscale Thermophoresis (MST)

Microscale Thermophoresis (MST) experiments were conducted using the Monolith NT.115 (201810-BR-N006) (Blue/Red) instrument from NanoTemper Technologies GmbH, Germany. Cell lysates from COS-1 cells expressing PXR-eGFP served as the source of fluorescently labeled PXR, allowing the study of proteins in near-native conditions. COS-1 cells were transfected with 1000 ng of GFP-fused PXR (PXR-eGFP) using Lipofectamine 3000 and lysed 48 h post-transfection with RIPA buffer. To assess MI891 binding to PXR, cell lysates were diluted 2x with MST buffer (10 mM Na-phosphate buffer, pH 7.4, 1 mM MgCl_2_, 3 mM KCl, 150 mM NaCl, 0.05% Tween-20) to achieve optimal fluorescence levels. A titration series of MI891 (0–30 μM) was incubated with the diluted cell lysates. Measurements were taken in premium-coated capillaries (NanoTemper Technologies GmbH, MO-K025) using an LED source (Blue LED: Ex: 470(±10)/Em: 520(±10) nm) with an excitation wavelength of 470 nm and a 50% maximum stimulation time (MST). The excitation power was set at 25 °C. Binding affinity was evaluated using the Hill model, with each data point representing mean fraction bound values from three independent experiments. The bound fraction of PXR-eGFP was determined, and fitting was performed using the Hill model method in MO Affinity Analysis v2.3 software (NanoTemper Technologies). Results were fitted to specific binding with Hill slope equations from GraphPad Prism. The protocol was adopted from the published report.^50^

### Fluorescence (eGFP) Intensity Assay

COS-1 cells were transfected with 150 ng of PXR-eGFP (NR1I2_pcDNA3.1­(+)-N-eGFP, GenScript Biotech) and 150 ng of CRBN (GenScript - CRBN_OHu21180D_pcDNA3.1+/C-(K)-DYK) in a 48-well plate using Lipofectamine 3000, following the manufacturer’s protocol. After 24 h, the transfected cells were treated with varying concentrations of the MI1013 compound and incubated for another 24 h. The eGFP fluorescence was then checked and imaged using the Invitrogen EVOS imaging system. Fluorescence intensity was measured using the Spark Multimode Microplate Reader (V2.2) in Fluorescence Intensity Scan Top Reading mode. The excitation wavelength was set between 440 and 500 nm, and the emission wavelength was set at 545 nm (Monochromator), with a gain of 100 and an integration time of 40 μs. Graphs were generated using GraphPad Prism.

### HiBiT Assay

For HiBiT assay, HepG2 cells (40,000 cells/well) were seeded in a 48-well plate. The following day, cells were transfected with 0.3–100 ng of PXR-HiBiT (GenScript-HiBiT_NR1I2_OHu23779D_pcDNA3.1+/C-(K)-DYK) and 0.3–100 ng of CRBN (GenScript-CRBN_OHu21180D_pcDNA3.1+/C-(K)-DYK) in a 1:1 ratio using Lipofectamine 3000 (ThermoFisher Scientific), following the manufacturer’s protocol. After 24 h, cells were treated with varying concentrations of MI1013, and vehicle (0.1% DMSO). The plates were incubated for 8 h, and the HiBiT NanoLuc signal was measured using the Nano-Glo HiBiT Lytic Detection System (Promega; Hercules, CA) in a Synergy microplate reader (Biotek) under luminescence mode with a read time of 0.2 s/well, gain set as auto, and integration time 2 s. Results are reported as relative abundance or response (%) change relative to DMSO control cells. A response of 100% indicates that the HiBiT luminescence signal equals that of the DMSO-treated controls, while 0% indicates it equals that of the media-only controls. The DC_50_ value was calculated using a curve fit—log­(inhibitor) vs response—with a variable slope (four-parameter) logistic fitting using GraphPad Prism. Standardization with different ratios and transfection levels of PXR-HiBiT:CRBN showed that at higher transfection levels (greater than 0.3 ng), degradation was not observed due to the redundant expression level of the protein.

### Molecular Docking

Docking studies were conducted using the Schrödinger Maestro 14.1 software package. Compound conformations were generated and energy-minimized with the LigPrep tool, utilizing the OPLS4 force field and Epik for ionization (pH 7.40 ± 2.0). The 3D crystal structures of PXR LBD (PDB codes: 8SVT) were sourced from the RCSB Protein Data Bank. Receptor preparation involved optimization with PROPKA, charge calculation using OPLS4, removal of cocrystal ligands, and addition of hydrogens and missing side chains, all performed with the Protein Preparation Workflow. Docking was performed using Glide within a box of 20 Å centered in the cocrystallized ligands and using the extra precision XP scoring function.^51^ Redocking of crystal-bound structures was performed as needed to confirm ligand poses. Images were generated using PyMOL v2.5.

### Protein–Protein Docking

The crystal structures of PXR (PDB ID: 5 × 0R) and CRBN-DDB1 (PDB ID: 4CI3) were obtained from the protein data bank (PDB) database. The high-ambiguity driven protein–protein docking (HADDOCK molecular dynamics) v2.4 server was used for computational biomolecular interaction studies (protein–protein mode),^33^ and a simulated model was generated of the PXR:CRBN complex. After simulated structures were superimposed with docked PXR-SPA70 (PDB ID: 5 × 0R) and CRBN-DDB1 (PDB ID: 4CI3) and the distance was measured using the PyMOL tool, the designed MI1013 distance was measured using ChemDraw 3D after MM2 minimization and MM2 dynamics.^52^ Further results were visualized and analyzed using PyMOL. Protein–protein interaction active residues predicted with the HDOCK Web server^53^

The obtained HADDOCK results of the PXR:CRBN complexes were used to predict the values of the binding affinity (Δ*G*) (kcal/mol^–1^) and dissociation constant [nM] at 37.0 °C using the PROtein binDIng enerGY prediction (PRODIGY) online server tool, and LigPlot+ software was used to obtain two-dimensional protein–peptide interaction diagrams.^35^

### Western Blot Analysis

The harvested HepaRG cells were lysed in RIPA lysis buffer (Cat. no 89900, Thermo Scientific) containing Halt protease inhibitor cocktail inhibitors (Thermo Scientific). Protein concentration was measured using the Pierce BCA Protein Assay Kit (Cat. no. 23225, Thermo Scientific), samples were diluted in 4 × Laemmli sample buffer (Cat. no. 1610747, BioRad) supplemented with mercaptoethanol and heated at 95 °C for 5 min. Equal amounts of protein were loaded in 4–20% Mini-PROTEAN TGX Stain-Free Protein Gels (12 well. cat. no. 4561095 or 15 well, cat. no. 4568096, Bio-Rad), electrophorised, and transferred onto Trans-Blot Turbo Midi-size PVDF Membrane (cat. no. 1704273, Bio-Rad), and blocked with EveryBlot Blocking buffer for 10 min (cat. no. 12010020, Bio-Rad). The membranes were incubated overnight at 4 °C with the following antibodies: PXR polyclonal antibody (1:500, PA5–41170, Invitrogen), RXRA polyclonal antibody (1:1000, 21218–1-AP, Proteintech), and GSPT1 polyclonal antibody (1:1000, 10763-a-AP, Proteintech), washed with TBS-T for 3 times, and followed by incubation with goat antirabbit IgG (H + L) Secondary antibody, HRP (1:200, 000, 1 h, cat. no. 31460, Invitrogen). Chemiluminescence signals were detected using SuperSignal West Femto Maximum Sensitivity Substrate (cat. no. 34096, Thermo Scientific) and visualized by the ChemiDoc MP Imaging system (Universal Hood III, #731BR02821, Bio-Rad), and the bands were quantified using β-actin (1:2000, recombinant rabbit conjugated monoclonal antibody (JF53-10), cat. no. MA5-32540, Invitrogen) as loading control with ImageJ (version 1.54g) software.^54^

### DigiWest

DigiWest was performed as published^39^ using 12 μg of cellular protein from HepaRG lysates. In brief, the NuPAGE system (Life Technologies) was used for gel electrophoresis and blotting onto PVDF membranes. Proteins were biotinylated on the membrane using NHS-PEG12-Biotin (50 μM) in PBST for 1 h. Sample lanes were cut into 96 strips (0.5 mm each) and placed in one well of a 96-well plate before adding 10 μL of elution buffer (8 M urea, 1% Triton X-100 in 100 mM Tris-HCl, pH 9.5). Each strip or protein fraction was incubated with a distinct Neutravidin-coated MagPlex bead population (Luminex, Austin, TX, USA). Coupling was performed overnight and nonbound binding sites were blocked with 500 μM deactivated NHS-PEG12-Biotin for 1 h. By pooling all 96 protein-loaded bead populations, the original sample lane was reconstituted.

Five μL aliquots of bead mix were added to 96-well plates containing 50 μL assay buffer (Blocking Reagent for ELISA (Roche) supplemented with 0.2% milk powder, 0.05% Tween-20 and 0.02% sodium azide). Upon discarding of the assay buffer, 30 μL of primary antibody (diluted in assay buffer) was added per well. After overnight incubation at 15 °C, the bead-mixes were washed twice with PBST and species-specific PE-labeled (Phycoerythrin) secondary antibodies (Dianova, Hamburg, Germany) were added for 1 h at 23 °C. Beads were washed twice with PBST before readout on a Luminex FlexMAP 3D instrument.

57 primary antibodies (see Table S5) were selected from a collection of >1,500 available antibodies, all of which are performance-evaluated and routinely used in DigiWest. Peak integration was performed using an Excel-based analysis tool. Signal intensities (AFI = accumulated fluorescent intensity) were normalized to the total protein amount loaded onto the beads. A total of 66 peaks were identified, with 50 of those peaks yielding reliable signals (75.5%, AFI > 50). The entire DigiWest data set (as normalized AFI) is enclosed as Supporting Information. The software package MeV 4.9.0 was used for heatmap generation and differential expression analysis (Wilcoxon-Rank sum Test).^55^

### Statistics and Data Visualization

The curve-fitting software GraphPad Prism 10.3.1 (GraphPad Software, La Jolla, CA) was used to generate the dose–response curves (DRC) and determine the IC_50_ and EC_50_ values. We performed statistical calculations by using a one-way analysis of variance (ANOVA) with Tukey’s multiple-comparison test, Wilcoxon test, or a paired *t* test when comparing two groups. We used SRplot for PCA analysis in RT-qPCR PHH experiment after normalization of values (Figure S10) and PCA analysis.^56^

## Supplementary Material











## Notes

Plasma and microsomal stability of fluorinated compounds in human plasma and microsomes; comparison of SPA70 and MI891 in antagonist mode and inverse agonistic mode; cytotoxicity of compounds; *in silico* analysis of ADME and kinome profile; off-target inhibition of kinases by MI891; *In silico*: structure-based MI1013 design and selection; PXR-CRBN docking score; PXR/CRBN interaction sites; PXR/CRBN complex interaction sites; PXR-CRBN binding affinity; Ternary complex (PXR: MI1013:CRBN); analysis of PXR degradation, binding, and cytotoxicity; analysis of MI1013 and its derivatives; MI1013 degradation on LS174T cells and PXR specificity profile; DigiWest protein profiling using 57 antibodies in HepaRG cells; crosstalk of heterodimerization between PXR and CAR; comparison of mRNA expression in primary human hepatocytes (PHH) using microarray data (GSE90122) versus qPCR; purity of the final compounds was determined by UPLC MS and was 95% or higher; and NMR data; DigiWest antibody; RT-qPCR primers (PDF)
Molecular formula strings docking data (CSV)
Docking data for PXR LBD (PDB)
Docking data for CAR LBD (PDB)
Docking data for PROTAC MI1013 PXR:CRBN (PDB)

## Abbreviations Used

Å: angstrom(s)
μ: micro
ADME: absorption, distribution, metabolism and excretion
CYP2B6: cytochrome P450 family two subfamily B member six
CYP3A4: cytochrome P450 family three subfamily A member four
DC_50_: half maximal degradation concentration
DDI: drug–drug interaction
DMSO: dimethyl sulfoxide
EC_50_: half maximal effective concentration
EGFP: enhanced green fluorescent protein
Emax: maximal calculated activation
IC_50_: half-maximum inhibitory concentration
LBD: ligand-binding domain
LBP: ligand-binding pocket
PDB: protein data bank
PEG: polyethylene glycol
P-gp: P-glycoprotein
PHH: primary human hepatocyte
PROTAC: proteolysis targeting chimera
PXR: pregnane X receptor
SRC-1: steroid receptor coactivator one
t1/2: half-time
TR-FRET: time-resolved fluorescence energy transfer
