# Supplementary material for: Discovery of PXR Antagonist MI891 and PXR Degrader MI1013 and Their Roles in Hepatic Gene Regulation
Source: J Med Chem. 2025 Jul 12;68(14):14271–99. doi: 10.1021/acs.jmedchem.4c03134 (PMC12305494; doi:10.1021/acs.jmedchem.4c03134)
Supplement: Supplementary file 1 [file jm4c03134_si_001.pdf]

## Supporting Information

# Discovery of PXR antagonist MI891 and PXR degrader MI1013 and their roles in hepatic gene regulation

Rajamanikkam Kamaraj[1f], Ivana Mejdrová[2f], Maria Krutakova[1], Tomas Smutny[1], Kryštof Škach[2], Klara Dohnalova[3,4], Lucie Smutna[1], Dharani Sai Sreekanth Nellore[1], Jan Dusek[1], Karel Chalupsky[3], Jana Hricová[2], Thales Kronenberger[5], Aaron Stahl[6], Markus Templin[6], Albert Braeuning[7], Radim Nencka[2,]* and Petr Pavek[1,]*

[1]Department of Pharmacology and Toxicology, Faculty of Pharmacy in Hradec Kralove, Charles University, Akademika Heyrovskeho 1203, 500 05, Hradec Kralove, Czech Republic; [2]Institute of Organic Chemistry and Biochemistry, Czech Academy of Sciences, Flemingovo nám. 2, 166 10 Prague 6, Czech Republic; [3]Czech Centre for Phenogenomics, Institute of Molecular Genetics of the Czech Academy of Sciences, Vídeňská 1083, 142 20, Prague; Czech Republic; [4]First Faculty of Medicine, Charles University, Katerinska 32, 112 08, Prague, Czech Republic; [5]DZIF Tübingen partner site, University Hospital Tübingen, 72076, Tuebingen, Germany; [6]NMI - Natural and Medical Sciences Institute at the University of Tuebingen, Markwiesenstr. 55, 72770 Reutlingen, Germany; [7]Department Food Safety, German Federal Institute for Risk Assessment, Max-Dohrn-Str. 8-10, Berlin, 10589, Germany.

*Corresponding author; address: [1]Department of Pharmacology and Toxicology, Faculty of Pharmacy in Hradec Kralove, Charles University, Akademika Heyrovskeho 1203, 500 05, Hradec Kralove, Czech Republic; [2]Institute of Organic Chemistry and Biochemistry, Czech Academy of Sciences, Flemingovo nám. 2, 166 10 Prague 6, Czech Republic; email address: pavek@faf.cuni.cz; and radim.nencka@uochb.cas.cz.

**Contents:**

**Page S2,** Table S1. Plasma and microsomal stability of fluorinated compounds in human plasma and microsomes

**Page S3,** Figure S1: Comparison of SPA70 and MI891 in antagonist mode and inverse agonistic mode.

**Page S4,** Figure S2: Cytotoxicity and in silico ADME properties.

**Page S5,** Figure S3: *In silico* membrane permeability.

**Page S5**, Table S2: Off-target activation of kinases by MI891

**Page S7,** Figure S4: *In silico*: structure-based MI1013 design and selection.

**Page S8,** Table S3. PXR-CRBN docking score.

**Page S8,** Figure S5: PXR/CRBN interaction sites.

**Page S8,** Figure S6: PXR/CRBN complex interaction sites.

**Page S9,** Table S4. PXR-CRBN binding affinity.

**Page S10,** Figure S7: Ternary complex (PXR:MI1013:CRBN).

**Page S11,** Figure S8: Analysis of PXR degradation, binding, and cytotoxicity.

**Page S12,** Figure S9: Analysis of MI1013 and its derivatives.

**Page S13,** Figure S10: MI1013 degradation on LS174T cells and PXR specificity profile.

**Page S14,** Figure S11: DigiWest protein profiling using 60 antibodies in HepaRG cells.

**Page S15,** Figure S12: Crosstalk of heterodimerization between PXR and CAR.

**Page S16-17,** Figure 13: HPLC chromatograms of MI891 and MI1013 PROTAC.

**Page S18-23,** General procedure-SI-I: Benzylic bromination and SI NMR.

**Page S24-38,** SI NMR spectra.
**Page S39-40,** Table S5. DigiWest antibody list
**Page S40-41,** Table S6. RT-qPCR primer list
**Page S41,** SI Reference.

**Table S1.** Plasma and microsomal stability of fluorinated compounds in human plasma and microsomes

| **Compound code** | **Plasma stability, $t^{1/2}$ (min)** | | **Microsomal stability, $t^{1/2}$ (min)*** | | **Microsomal clearance $CL^{int}$ (µl/min/mg)** | |
|---|---|---|---|---|---|---|
| | human | mouse | human | mouse | human | mouse |
| **compound 8** | >120 | >120 | 50±4 | 8±0 | 28±2 | 168±8 |
| **compound 10** | >120 | >120 | 55±9 | >45 | 26±4 | nd |
| **compound 11 (MI891)** | >120 | >120 | >45 (nd)# | 14±2 | nd# | 98±13 |
| **Propantheline** | 24±0 | 28±1 | | | | |
| **Verapamil** | | | 28±1 | 12±1 | 50±2 | 115±8 |

*0.5 mg/mL of human or mouse microsomes; nd – not determined due to high stability and low microsomal clearance. #estimation, the compound is stable over 45 minutes of the assay in the presence of human microsomes.

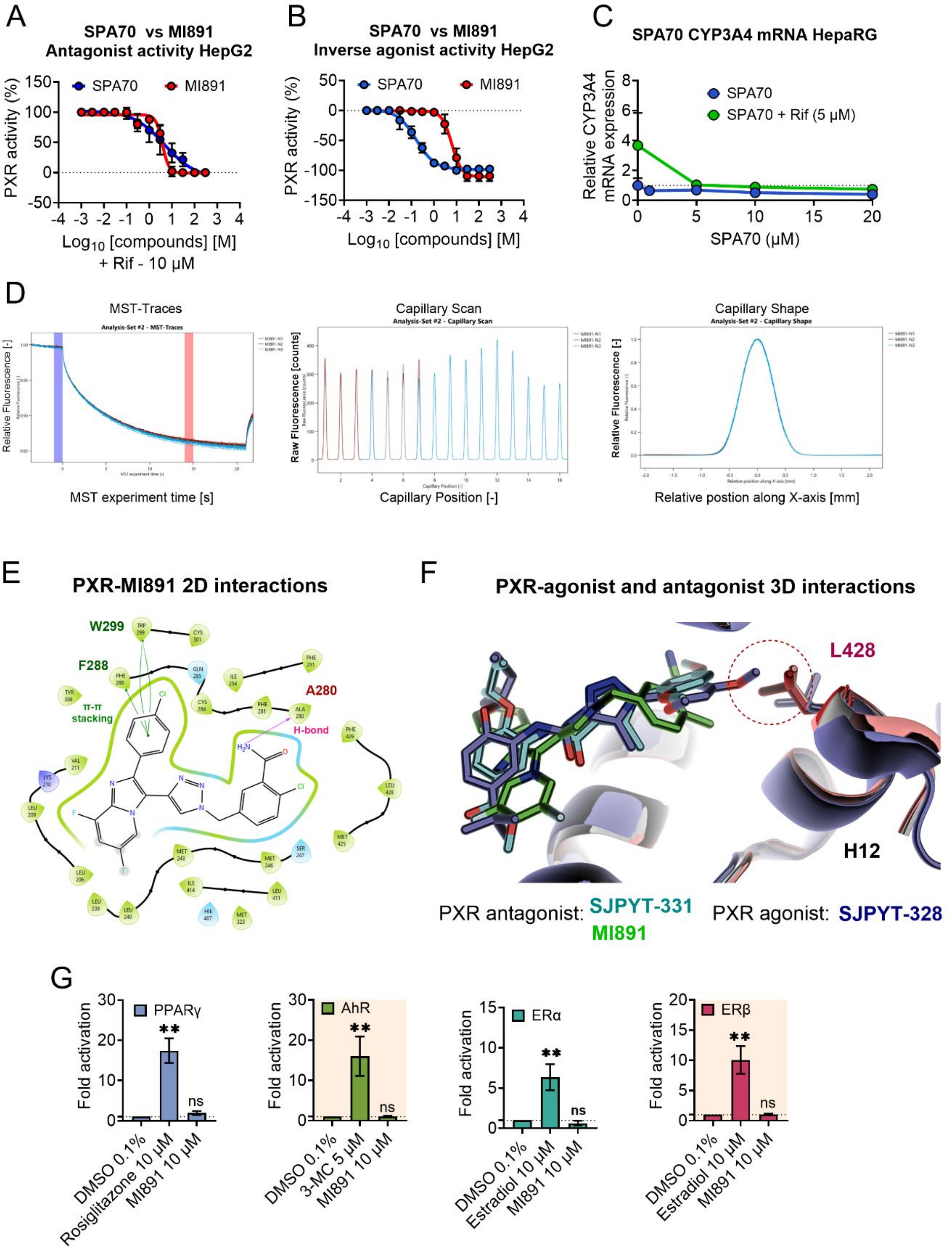


**Figure S1:** Comparison of SPA70 and MI891 in antagonist mode (A) and inverse agonistic mode (B). Dose-response curves of MI891 and SPA70 in HepG2 cells transfected with the PXR-responsive CYP3A4 promoter luciferase reporter construct at a constant rifampicin concentration (10 µM) (A) and with SPA70 or MI891 alone (inverse agonistic mode) (B). SPA70 represents 100% inhibition at 10 µM. (C) Dose-response effect of SPA70 on CYP3A4 mRNA expression in HepaRG cells treated with SPA70 alone or in combination with rifampicin (5µM). HepaRG cells have been treated for 48 h before RT-PCR analysis. (D) Microscale thermophoresis (MST) traces (left), capillary scan (center), and capillary shape (right) for quality check of the PXR-MI891 binding assay. The MST traces show temperature-dependent variations in fluorescence intensity, while the capillary scan and shape are used for fluorescence and absorption quality checks. (E) 2D interactions diagram of the PXR-MI891 docking result (PDB ID: 8SVT), showing the π-π stacking, hydrogen bond interactions, and surrounding

hydrophobic interactions. (F) Superimposed diagram of PXR bound with agonist (SJPYT-328) and antagonists (MI891 and SJPYT-331) to differentiate the agonist and antagonist interactions with helix 12 (H12). The interaction of the agonist with PXR residue (L428) is circled. (G) Extended nuclear receptor selectivity profile. Agonist assay for PPARγ, AhR, or ERα/β treated with 10 μM MI891 and the prototypical agonist molecules for control: 10 μM rosiglitazone for PPARγ, 3-MC for AhR, and estradiol for ERα/β. Data are mean ± SD, n = 3. *p < 0.05; **p < 0.001; ***p < 0.005 (Dunnett test; MI891 and reference ligands vs DMSO 0.1%).

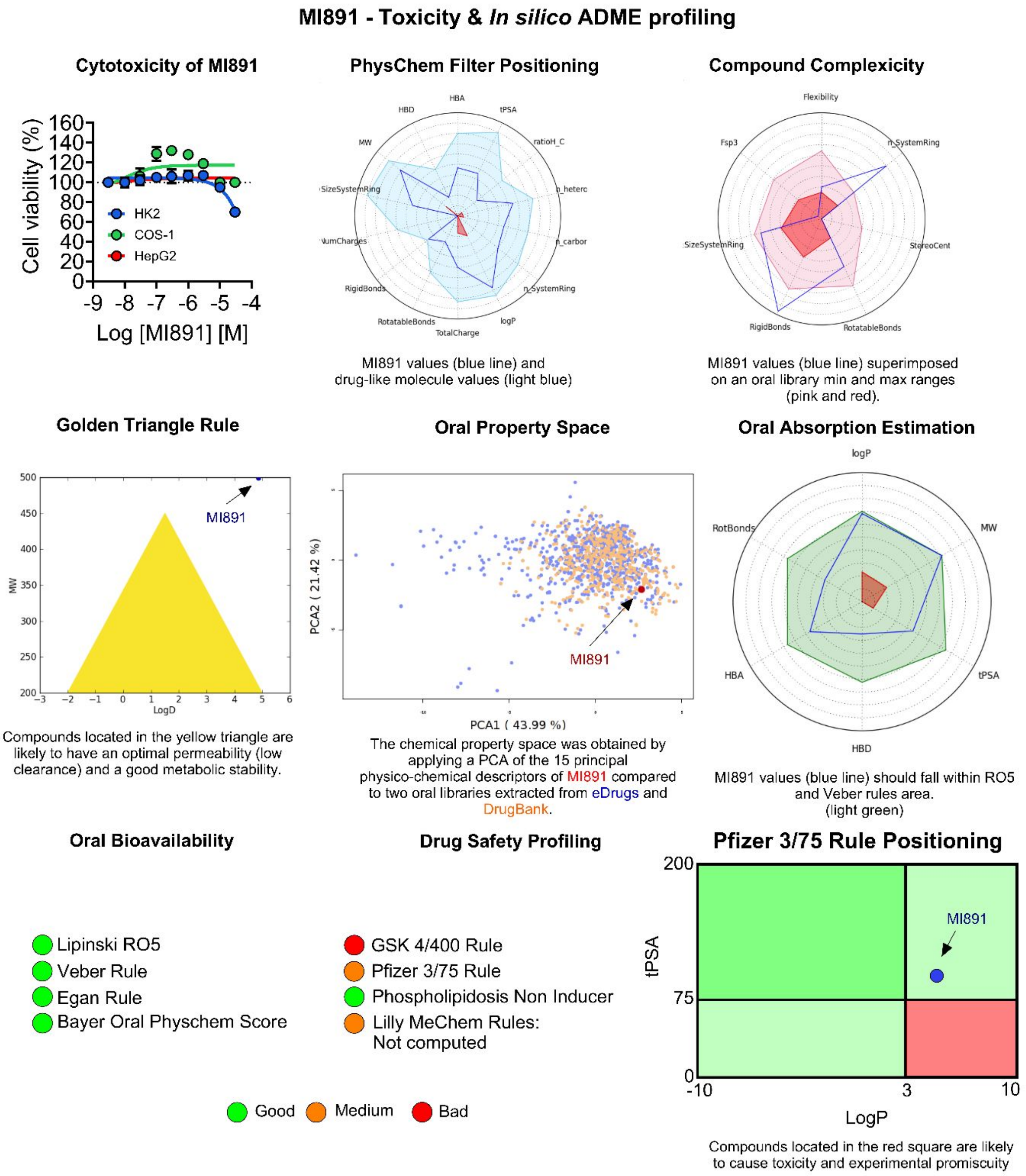


**Figure S2**: **Cytotoxicity and *in silico* ADME properties**. Cytotoxicity and *in silico* ADME properties. The cytotoxicity assay for MI891 was performed using three different cell lines (HepG2, HK-2, and COS-1) with an ATP-based CellTiter-Glo assay. The ADME properties were predicted using FAF-Drugs4 (INSERM, France).

**A**

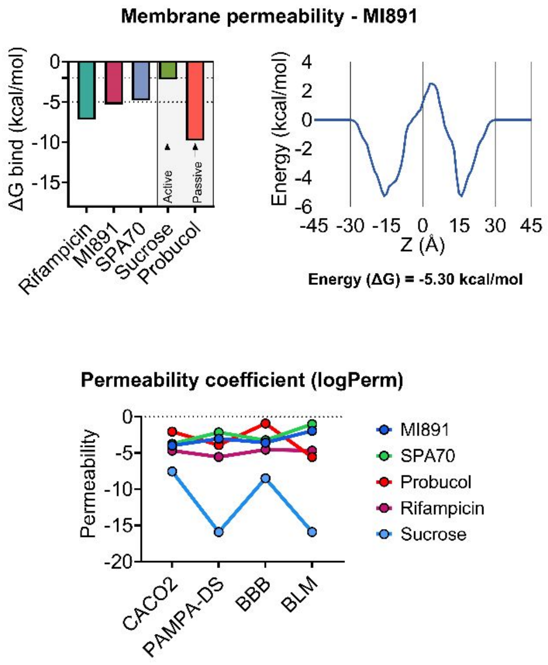


**Figure S3**: ***In silico* membrane permeability**. (A) Permeability of MI891 molecules across membranes was assessed using the PerMM web server (University of Michigan, USA). Probucol was used as the passive diffusion control and sucrose as the negative control for active transport.

**Table S2:** Off-target activation of kinases by MI891

| **Activity %** | |
|---|---|
| **Kinase** | **MI891 @ 1 µM** |
| Abl(h) | 104 |
| ALK(h) | 76 |
| AMPKα1(h) | 102 |
| ASK1(h) | 91 |
| Aurora-A(h) | 81 |
| CaMKI(h) | 95 |
| CDK1/cyclinB(h) | 96 |
| CDK2/cyclinA(h) | 106 |
| CDK6/cyclinD3(h) | 92 |
| CDK7/cyclinH/MAT1(h) | 94 |
| CDK9/cyclin T1(h) | 124 |
| CHK1(h) | 93 |
| CK1γ1(h) | 100 |
| CK2α2(h) | 109 |
| c-RAF(h) | 101 |
| DRAK1(h) | 105 |
| eEF-2K(h) | 112 |
| EGFR(h) | 103 |
| EphA5(h) | 106 |
| EphB4(h) | 97 |
| Fyn(h) | 81 |
| GSK3β(h) | 99 |
| IGF-1R(h) | 99 |
| IKKα(h) | 104 |

| | |
|---|---|
| IRAK4(h) | 112 |
| JAK2(h) | 101 |
| KDR(h) | 86 |
| LOK(h) | 94 |
| Lyn(h) | 88 |
| MAPKAP-K2(h) | 101 |
| MEK1(h) | 94 |
| MLK1(h) | 104 |
| Mnk2(h) | 86 |
| MSK2(h) | 110 |
| MST1(h) | 94 |
| mTOR(h) | 96 |
| NEK2(h) | 103 |
| p70S6K(h) | 105 |
| PAK2(h) | 84 |
| PDGFRβ(h) | 86 |
| Pim-1(h) | 53 |
| PKA(h) | 108 |
| PKBα(h) | 100 |
| PKCα(h) | 97 |
| PKCθ(h) | 90 |
| PKG1α(h) | 85 |
| Plk3(h) | 113 |
| PRAK(h) | 81 |
| ROCK-I(h) | 97 |
| Rse(h) | 109 |
| Rsk1(h) | 129 |
| SAPK2a(h) | 99 |
| SRPK1(h) | 98 |
| TAK1(h) | 90 |
| PI3 Kinase (p110b/p85a)(h) | 100 |
| PI3 Kinase (p120g)(h) | 102 |
| PI3 Kinase (p110d/p85a)(h) | 98 |
| PI3 Kinase (p110a/p85a)(h) | 92 |

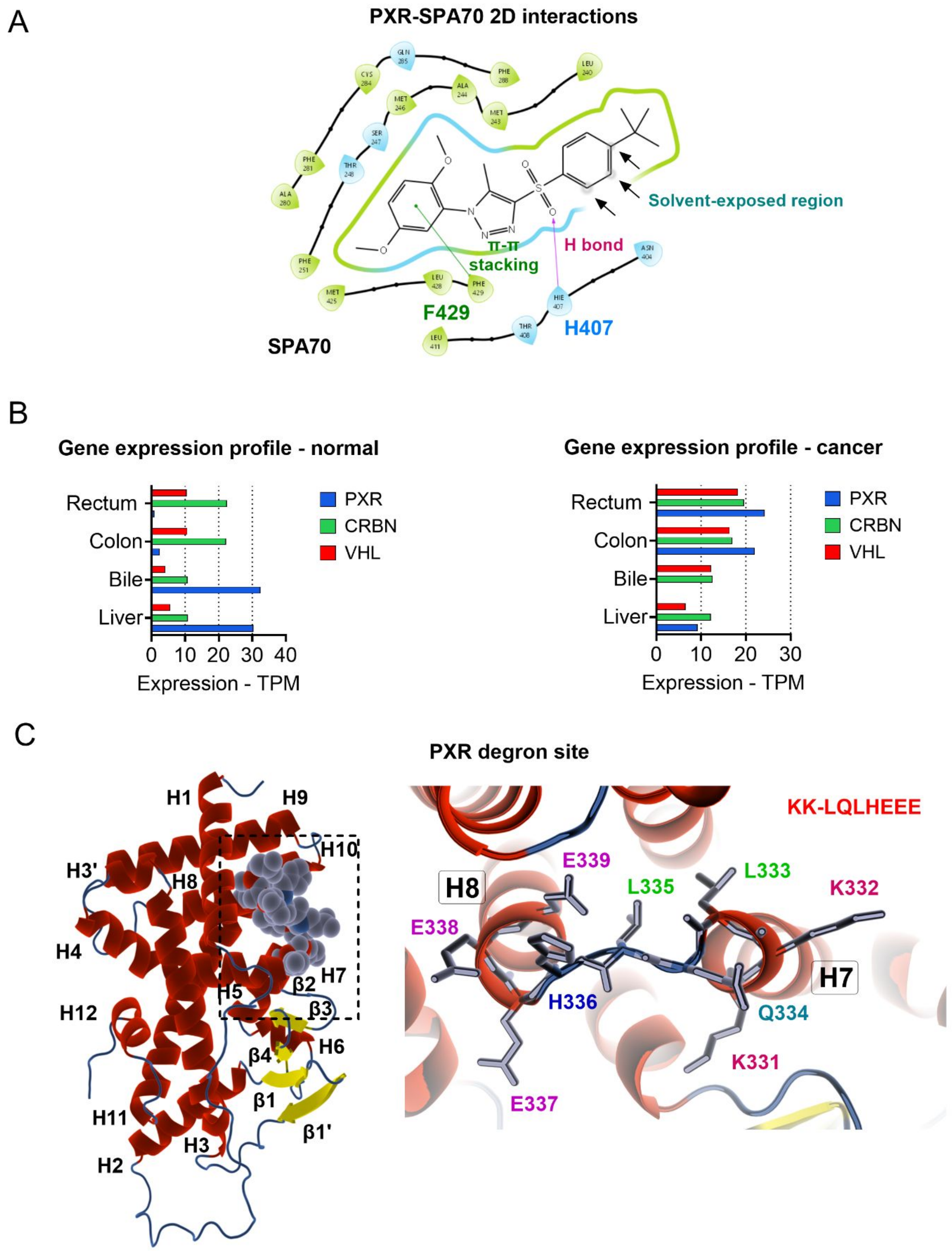


**Figure S4**: ***In silico*: structure-based MI1013 design and selection**. (A) 2D interactions diagram of the PXR-SPA70 docking result (PDB ID: 5X0R), showing the π-π stacking, hydrogen bond interactions, and surrounding hydrophobic interactions. Arrows indicate the solvent exposed site. (B) Gene expression levels of PXR, CRBN, and VHL in the liver and colon in normal and cancer human samples, data obtained from GEPIA2. TPM - transcripts per million. (C) PXR Degron Site Analysis: The degron site in the PXR protein was identified through a comprehensive proteome-wide analysis using the Degronopedia web server. The degron is indicated by a sphere in the PXR cartoon structure (left). The detailed amino acid residues are highlighted (right).

**Table S3.** PXR-CRBN docking score

| | | High ambiguity driven protein−protein docking (HADDOCK 2.4) | | | | | HDOCK server | |
|---|---|---|---|---|---|---|---|---|
| **Receptor** | **E3 ligase** | **HADDOCK score** | **Van der Waals** | **Electrostatic interactions** | **Desolvation energy** | **Z-score** | **Docking score** | **Confidence Score** |
| PXR | CRBN | -194.1 +/- 1.9 | -125.0 +/- 6.7 | -294.7 +/- 49.5 | -23.9 +/- 6.7 | -1.4 | -281.21 | 0.9324 |

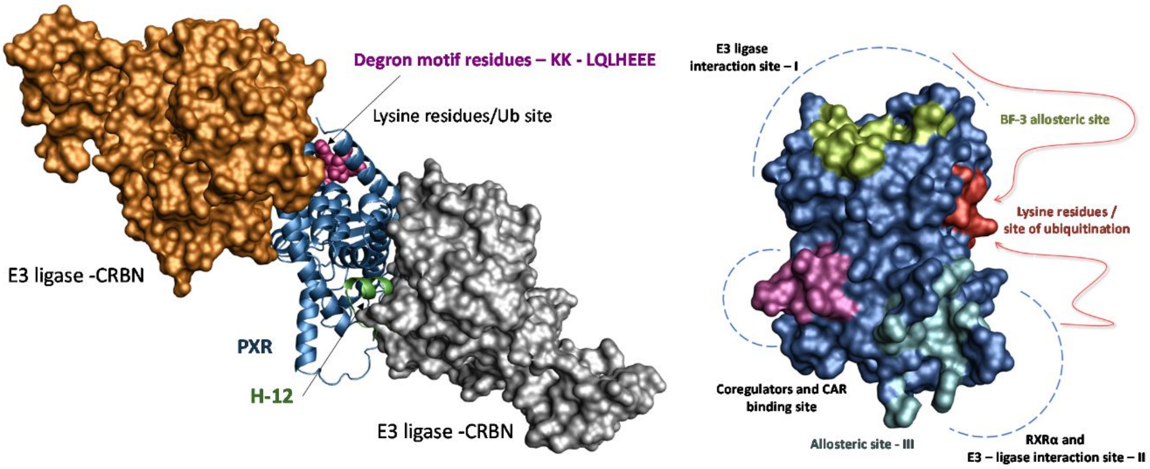


**Figure S5**: **PXR/CRBN interaction sites**. The three-dimensional (3D) PXR-CRBN interactions diagram depicts the E3 ligase CRBN as a brown and grey surface, while PXR is represented as a blue cartoon. Spheres indicate the degron or ubiquitin sites. On the right, the PXR surface structure highlights the E3 ligase and co-regulator interaction sites, as well as the PXR allosteric sites.

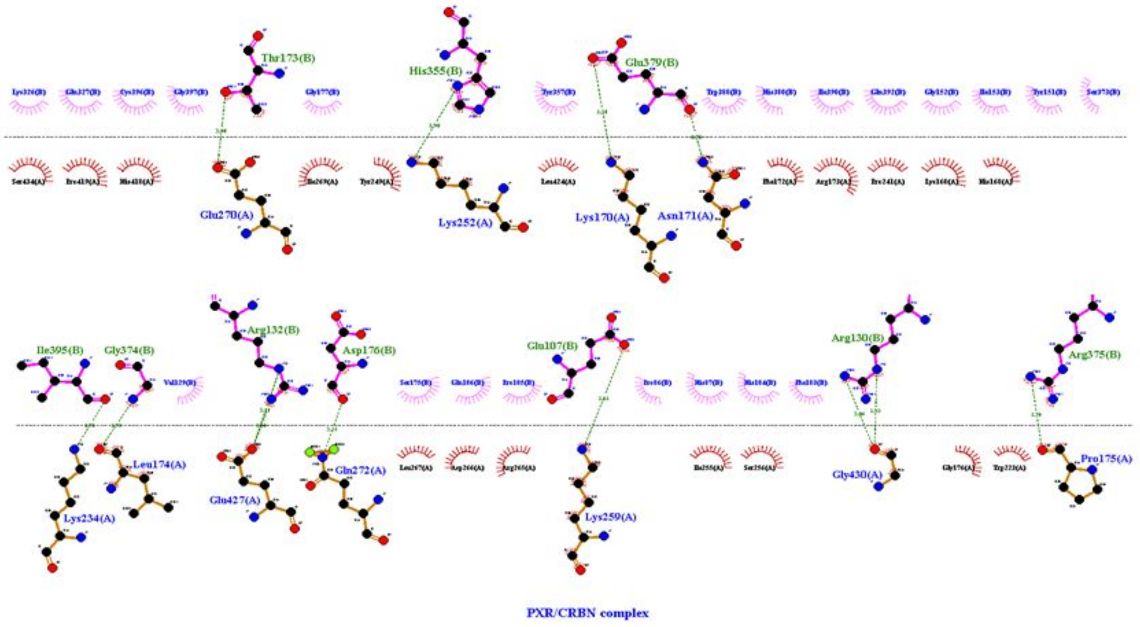


**Figure S6**: **PXR/CRBN complex interaction sites**. The two-dimensional (2D) PXR-CRBN interactions diagram (DIMPLOT) shows hydrophobic interactions as arches and hydrogen bond interactions as green dotted lines.

**Table S4.** PXR-CRBN binding affinity

| Binding affinity (PRODIGY & Area-Affinity) | | | |
|---|---|---|---|
| **ΔG [kcal/mol]** | **KD [M] (@ 37 °C)** | **KD [log(K)]** | **Energy [kcal/mol]** |
| -14.8 | 3.7e-11 | -6.5 | -8.9 |

## Ternary complex (PXR:MI1013:CRBN): PRosettaC docking

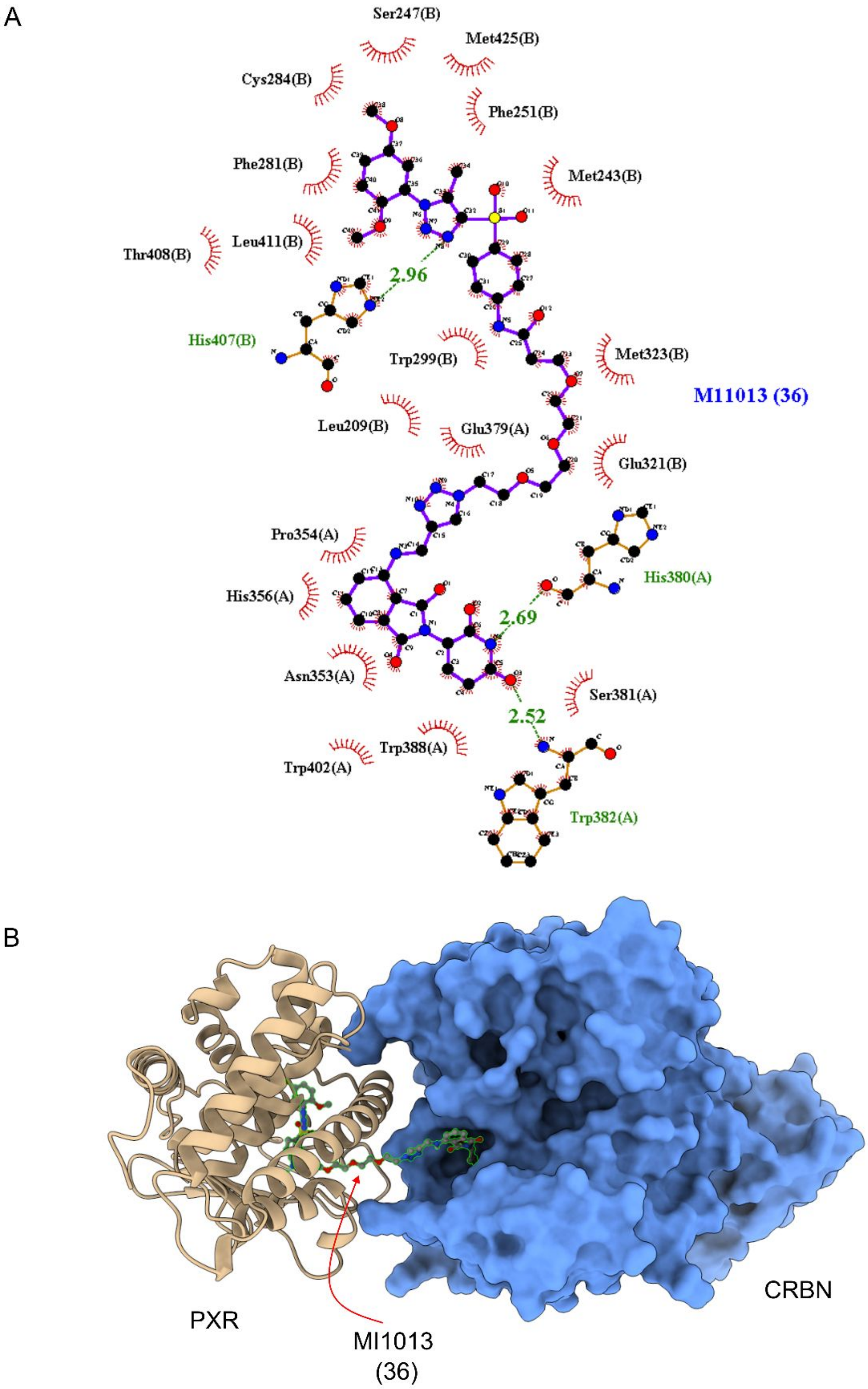


**Figure S7**: **Ternary complex (PXR:MI1013:CRBN)**. (A) The PRosettaC predicted ternary complex model includes a two-dimensional interaction diagram, where hydrogen bonds are shown as green dotted lines and hydrophobic interactions as red arches, identified using LigPlot+. (B) The three-dimensional ternary complex of MI1013 (36) bound to the PXR/CRBN

complex is depicted with PXR in brown, CRBN E3 ligase in light blue, and MI1013 (36) indicated in arrow.

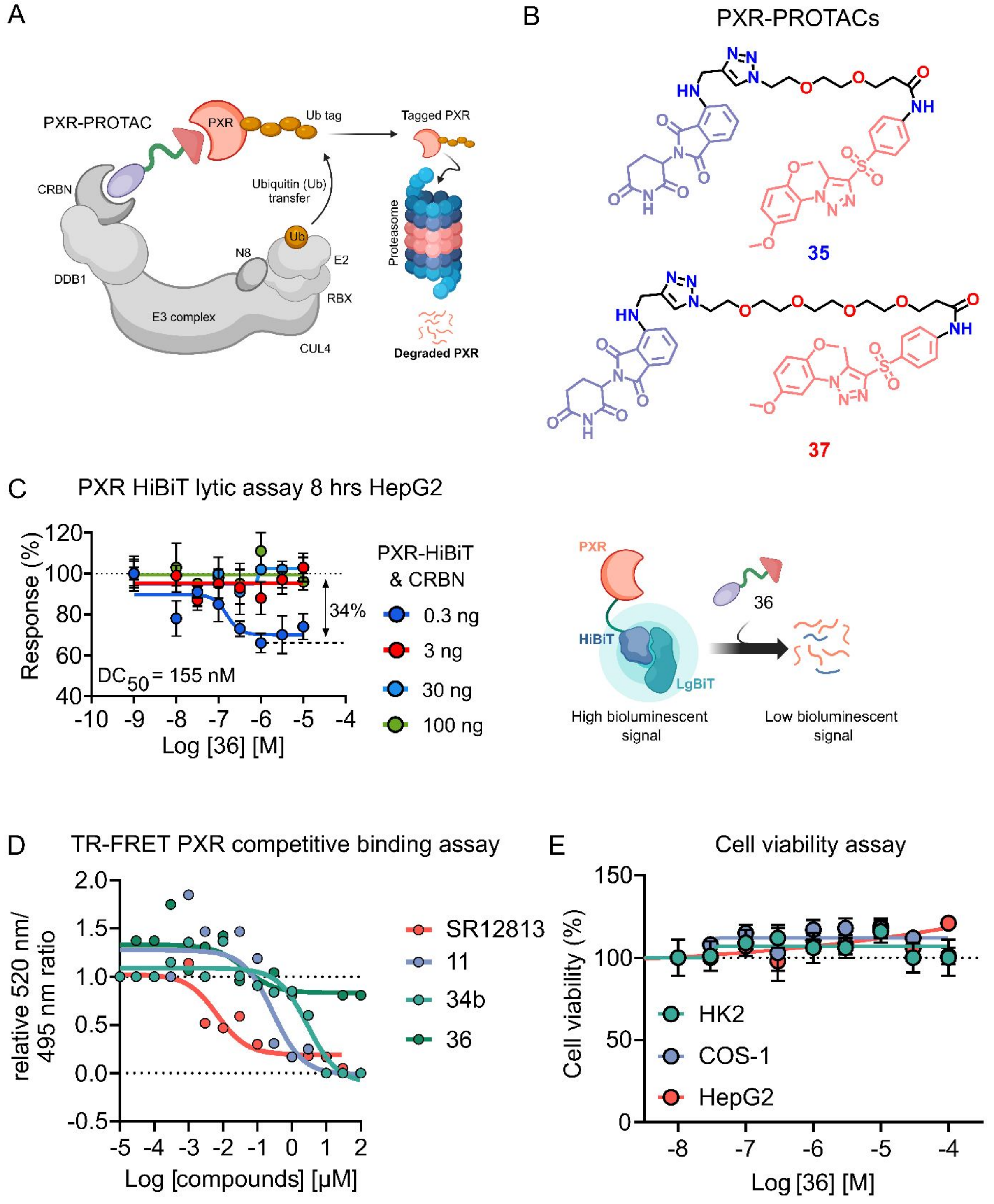


**Figure S8: Analysis of PXR degradation, binding, and cytotoxicity:** (A) Schematic representation of targeted protein degradation of PXR by a CRBN-based PROTAC. (B) PXR-PROTAC molecules with variable linker lengths. (C) Nano-Glo HiBiT lytic assay. PXR-HiBiT and CRBN (1:1) were transfected into HepG2 cells and treated with MI1013 at the indicated concentrations for 8 hours. Degradation is observed only at low expression levels. Left, schematic representation of HiBiT-tagged PXR binding to LgBiT, resulting in a high bioluminescent signal. When treated with PXR PROTAC or MI1013, the degradation of PXR leads to a low bioluminescent signal. (D) TR-FRET PXR competitive binding assay for compound 11, 34b, 36 and SR12813. (E) The cytotoxicity assay for MI1013 was performed using three different cell lines (HepG2, HK-1, and COS-1) with an ATP-based CellTiter-Glo assay.

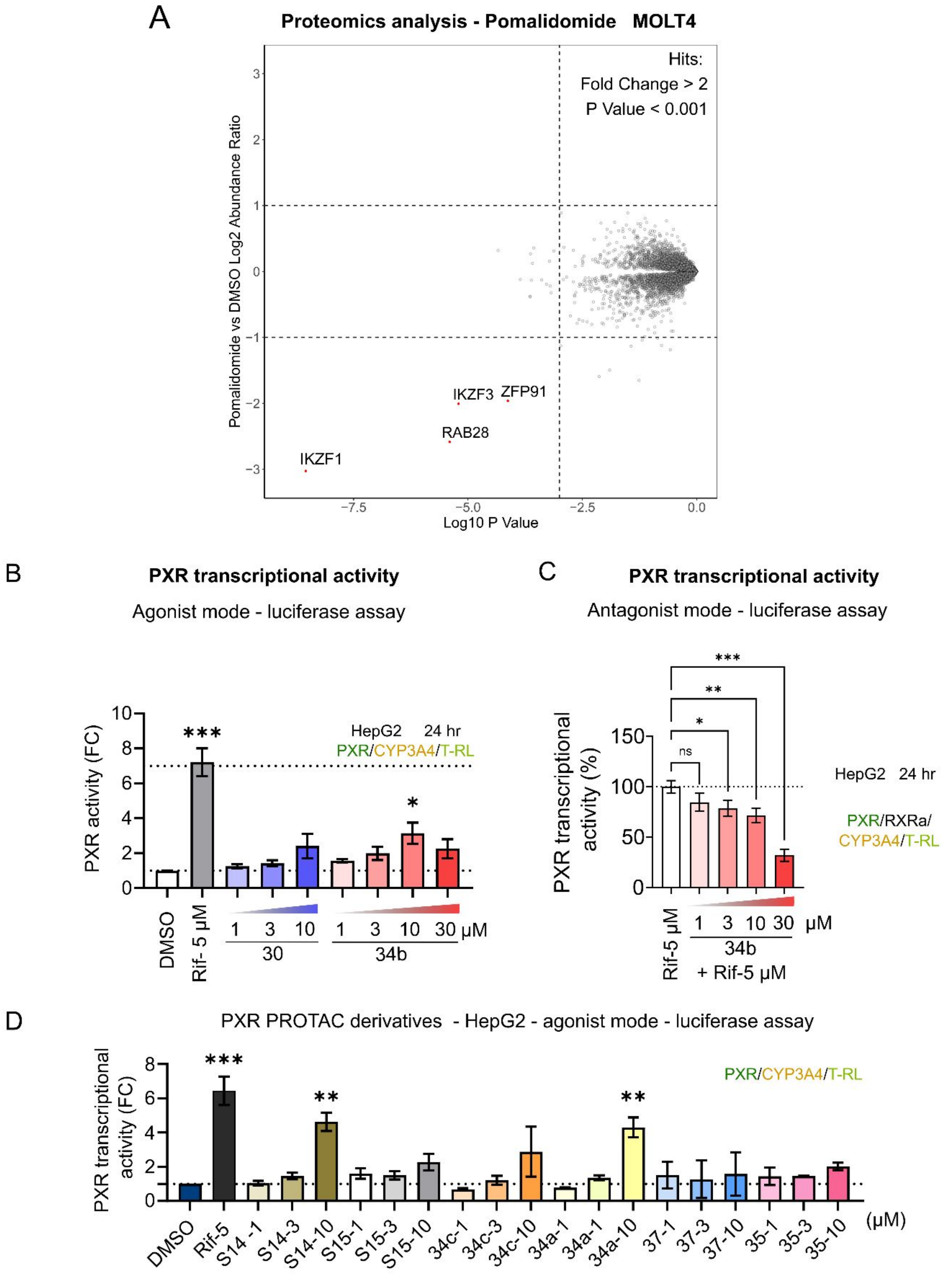


**Figure S9: Analysis of MI1013 and its derivatives.** (A) Quantitative proteomics analysis of MOLT4 cells treated with the positive control compound Pomalidomide. The volcano plot displays the differential protein expression profile, highlighting significantly downregulated zinc-finger proteins. (B) PXR agonistic assay for MI1013 derivatives, MI1002B and MI1011B. 5 µM rifampicin was used as a positive control. Data are presented as mean ± SEM (n=5). ***p < 0.001; **p = 0.003; other ns – non-significant (Dunnett's multiple comparison test; MI1002B and MI1011B at different concentrations or 5 µM rifampicin vs. DMSO). (C) Antagonistic activity: MI1011B was treated at specified concentrations alongside a constant 5 µM rifampicin treatment. Positive control was set at 100% PXR transcriptional activity. PXR activity is presented as PXR fold change (FC)

activity or PXR transcriptional activity (%) assessed based on the activation of the CYP3A4 gene promoter luciferase reporter construct in transiently transfected HepG2 cells. Data are presented as mean ± SEM. ns – non-significant (p values: 1 µM – 0.08, 3 µM – 0.02, 10 µM – 0.002, 30 µM – <0.001 vs. rifampicin 5 µM) using Dunnett's multiple comparison test. (D) PXR agonistic assay for other PROTAC derivatives. 5 µM rifampicin was used as a positive control. Data are presented as mean ± SEM. ***p < 0.001; p = 0.004 and 0.010; other ns – non-significant (Dunnett's multiple comparison test; PXR PROTAC derivatives at different concentrations or 5 µM rifampicin vs. DMSO).

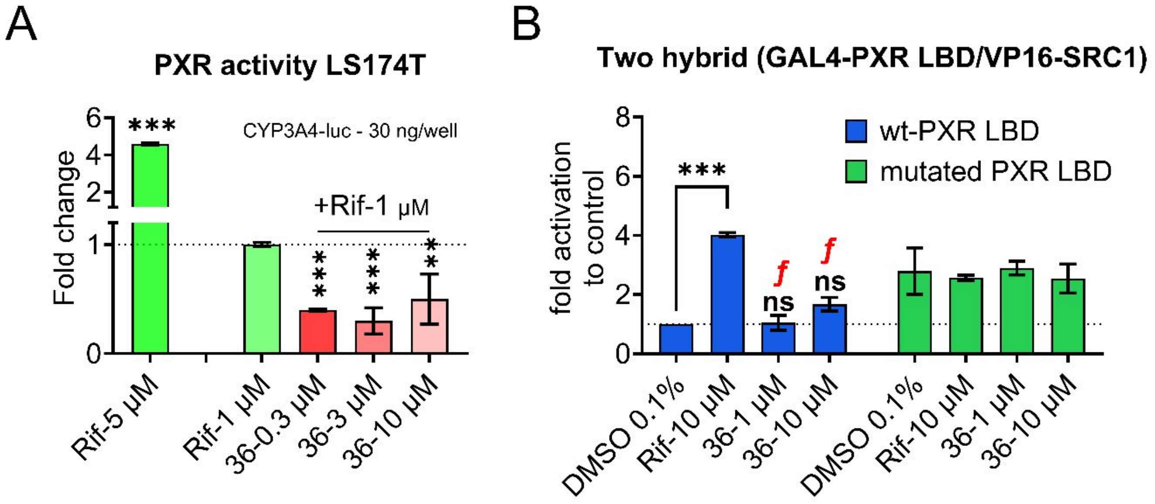


**Figure S10**: **MI1013-mediated degradation of endogenous PXR in LS174T cells and PXR LBD interaction profile**. (A) The antagonistic effect of MI1013 (36) was assessed by measuring the PXR-responsive *CYP3A4* gene-derived luciferase construct activity using a luciferase assay to indirectly evaluate PXR degradation in LS174T cells. CYP3A4-luc and RL-TK (30 ng/well) were transfected into LS174T cells, and luminescence was measured. Rifampicin was used as a positive control, and MI1013 (36) was tested at three different concentrations alongside a constant 1 µM rifampicin. Data are presented as mean ± SD, n = 3. *p < 0.05; **p < 0.001; ***p < 0.005 (Dunnett test; MI1013 vs Rif-1 µM). (B) Mammalian two-hybrid assay with wild-type (wt-PXR LBD) or triple mutant (S208W/S247W/C284W) obstructed PXR-LBD and co activator peptide SRC-1-VP16 in HepG2 cells. Data are presented as fold activation to the vehicle-treated GAL-4 wt-PXR-LBD/SRC-1-VP-16 transfected sample (set to 1). Statistical significance between the groups was tested by one-way ANOVA followed by Dunnett multiple comparisons test (DMSO to other molecules, *p < 0.05, **p < 0.01 or ***p < 0.001; ns – no significance; Rif 10 µM to other molecules: fp < 0.05, and fp < 0.01). All data are the mean ± SD, n = 3.

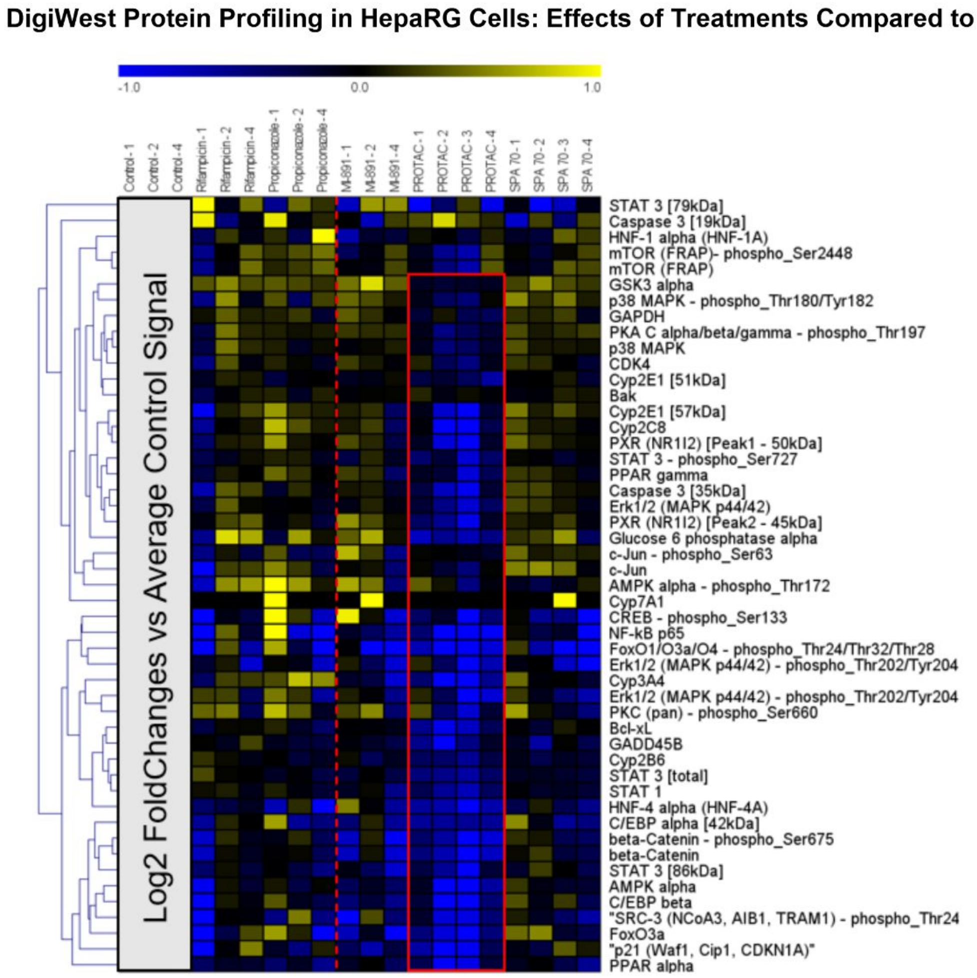


**Figure S11**: **DigiWest protein profiling using 60 antibodies in HepaRG cells.** The clustered heatmap shows differentially expressed genes in HepaRG cells. Protein levels were analyzed using DigiWest microarrays 48 hours after treatment with rifampicin (agonist), SPA70 or MI891 (antagonists), and MI1013 (36, degrader). Data are from four replicates (n = 4), with median-centered results. Statistical significance was determined using the Wilcoxon test ($p < 0.05$). The red dotted line separates the positive controls (rifampicin and propiconazole), while the red box highlights the MI1013-treated samples and their negative regulation. MI1013 (36) is named as PROTAC in the figure.

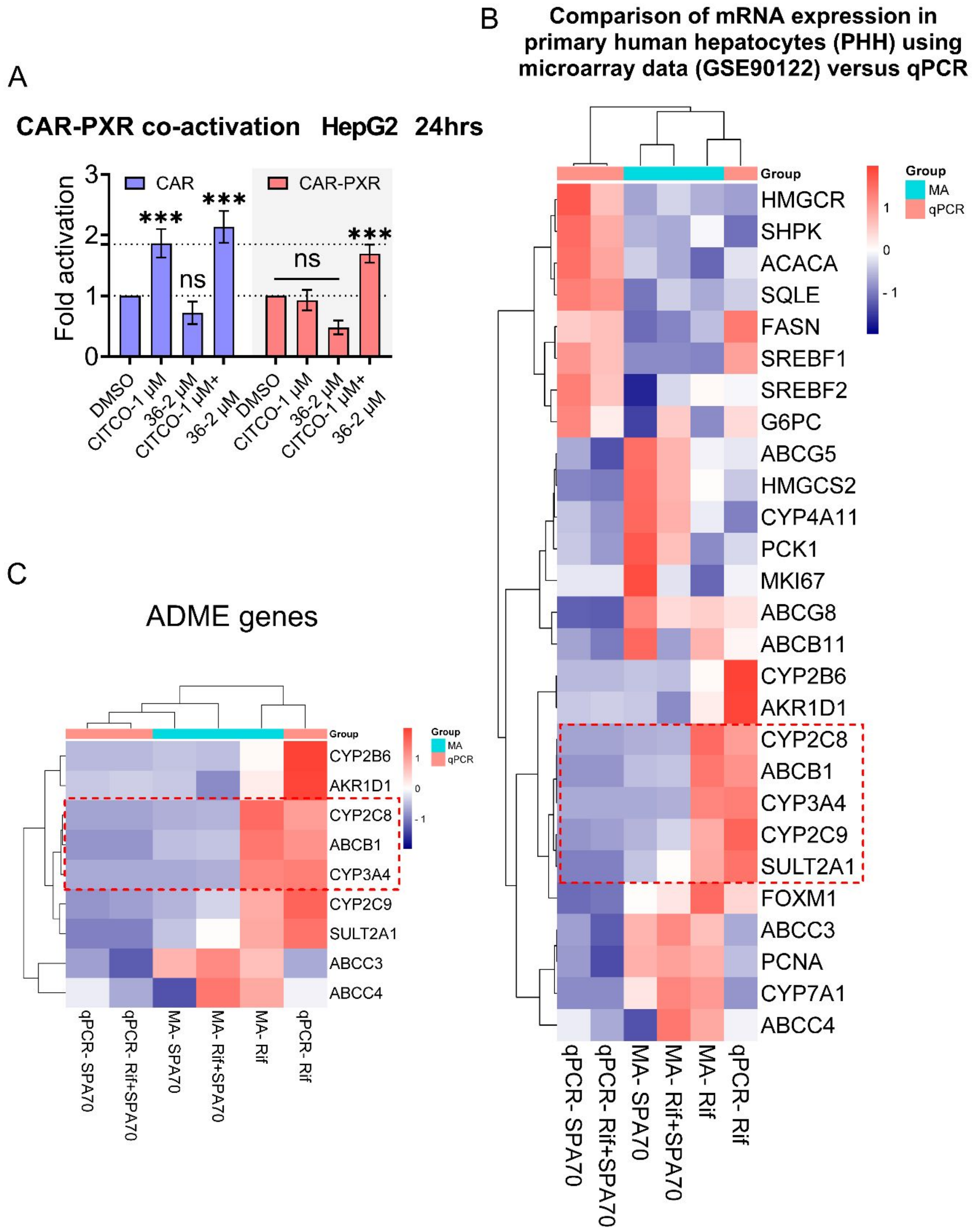


**Figure S12**: (A) Crosstalk of heterodimerization between PXR and CAR. The *CYP2B6* gene-derived luciferase construct was used to monitor CAR activation. CITCO was used as a positive control for CAR activation, and the MI1013 (36) degrader in combination with CITCO resumed CAR activation. Statistical significance between groups was tested by one-way ANOVA followed by Dunnett's multiple comparisons test (DMSO vs. other molecules, *$p < 0.05$, **$p < 0.01$, ***$p < 0.001$; ns – not significant). All data are presented as mean ± SD, n = 3. (B) Comparison of mRNA Expression in Primary Human Hepatocytes (PHH) Using Microarray Data (GSE90122) versus our RT-qPCR. The red dotted box highlights major ADME genes with similar expression patterns in both RT-qPCR and microarray data. (C) On the left, only the ADME gene cluster is presented for a more focused representation.

**A. MI891 (compound 11)**

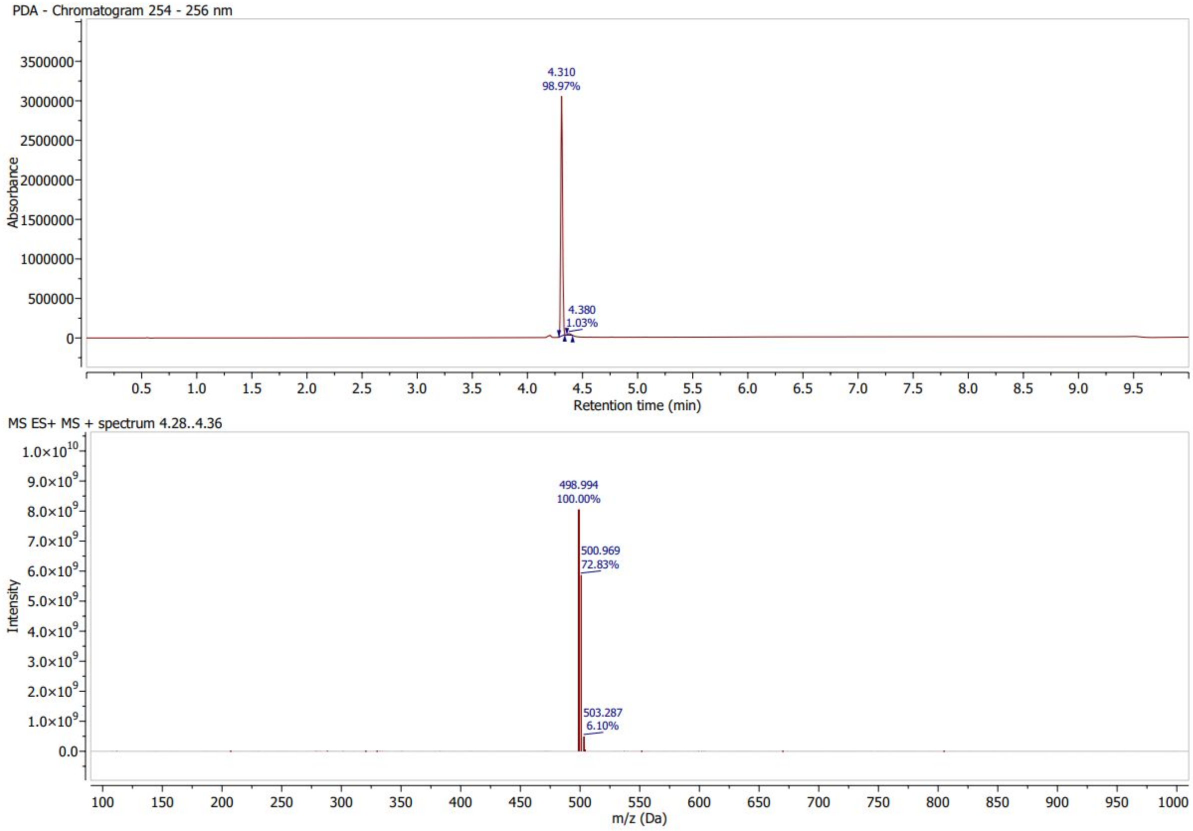


**B. MI1013**

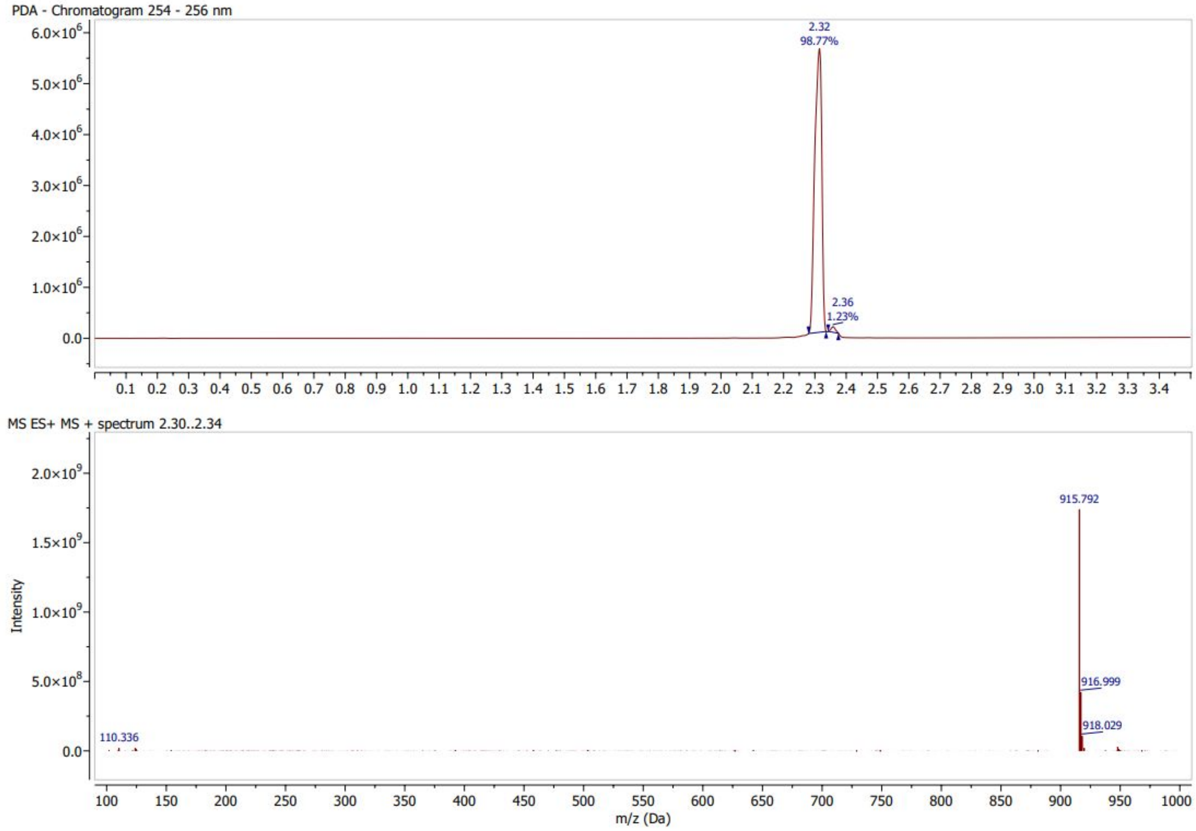

**Figure S13: Purity of the final compounds was determined by UPLC MS and was 95% or higher.** (A) MI891 (compound 11), (B) MI1013 PROTAC. Analytical High-Performance Liquid Chromatography (HPLC), low resolution mass spectra, UV absorbance and compound purity were measured on a Waters Ultra-High Performance Liquid Chromatography-Mass Spectrometry (UPLC-MS) system consisting of a Waters UPLC H-Class Core System, a UPLC photodiode array (PDA) detector and a Waters SQD2 or QDa mass spectrometer. The MS method used was electrospray ionization (ESI)+, cone voltage = 15 V, mass detector range 105 – 1000 Da. Two sets of HPLC conditions were used as indicated: (a) Waters Acquity UPLC BEH C18 column, 1.7 µm, 2.1 × 100 mm; LC method: H2O/CH3CN, 0.1% FA as a modifier, gradient 0 – 100 %, run length 7 min, flow 0.5 ml/min and (b) Waters Cortecs UPLC C18 column, 1.6 µm, 2.1 × 50 mm; LC method: H2O/CH3CN, 0.1% FA as a modifier, gradient 0 – 100 %, run length 3.5 min, flow 0.7 ml/min.

**General procedure-SI-I: Benzylic bromination**

Substituted phenyl derivative was dissolved in dry $CCl_4$ and degassed prior to an addition of NBS (1.5 eq) followed by the addition of catalytic amount of benzoylperoxide. The mixture was stirred at 70 °C overnight. After the completion, the mixture was evaporated and used as crude or diluted with EtOAc and washed with water and $Na_2S_2O_3$ solution. The organic phase was dried over sodium sulfate, evaporated and purified by flash column chromatography.

*5-(1-Bromoethyl)-2-chlorobenzamide (intermediate compound)* **S1**

Title compound was prepared according to the General procedure SI-I starting from 2-chloro-5-ethylbenzamide. After the completion, the mixture was evaporated, diluted with EtOAc and washed with water and $Na_2S_2O_3$ solution. The organic phase was dried over sodium sulfate, evaporated and purified by flash column chromatography. Mobile phase petrolether/EtOAc (10-50 %). Yield: 189 mg (79 %).$^1$H NMR (401 MHz, DMSO-$d_6$) δ 7.95 (s, 1H), 7.66 (s, 1H), 7.62 – 7.53 (m, 2H), 7.51 – 7.45 (m, 1H), 5.52 (q, $J$ = 6.9 Hz, 1H), 1.97 (d, $J$ = 6.9 Hz, 3H). $^{13}$C NMR (101 MHz, DMSO) δ 168.01, 142.38, 137.49, 130.14, 129.50, 129.30, 127.24, 127.24, 49.24, 26.46. HRMS: calcd for [M + H], 261.96288; found, 261.96296.

*5-(1-Azidoethyl)-2-chlorobenzamide (intermediate compound)* **S2**

5-(1-Bromoethyl)-2-chlorobenzamide was dissolved in DMF (3mL) and $NaN_3$ (1 eq) was added. The reaction mixture was stirred at r.t. till the completion of the reaction (2 h). The mixture was diluted with water and extracted with EtOAc. The organic phase was dried over sodium sulfate and evaporated, The residue was purified by column chromtography. Mobile phase petrolether/EtOAc (10-50 %). Yield: 189 mg (79 %). $^1$H NMR (401 MHz, DMSO-$d_6$) δ 7.94 (s, 1H), 7.67 – 7.61 (m, 1H), 7.53 – 7.47 (m, 1H), 7.45 (d, $J$ = 7.8 Hz, 2H), 4.91 (t, $J$ = 6.8 Hz, 1H), 1.47 (d, $J$ = 6.8 Hz, 3H). $^{13}$C NMR (101 MHz, DMSO) δ 168.36, 140.39, 137.79, 130.39, 130.36, 129.58, 128.94, 127.24, 59.59, 21.45. HRMS: calcd for [M + H], 225.0543; found, 225.0547.

**Scheme SI 1.**

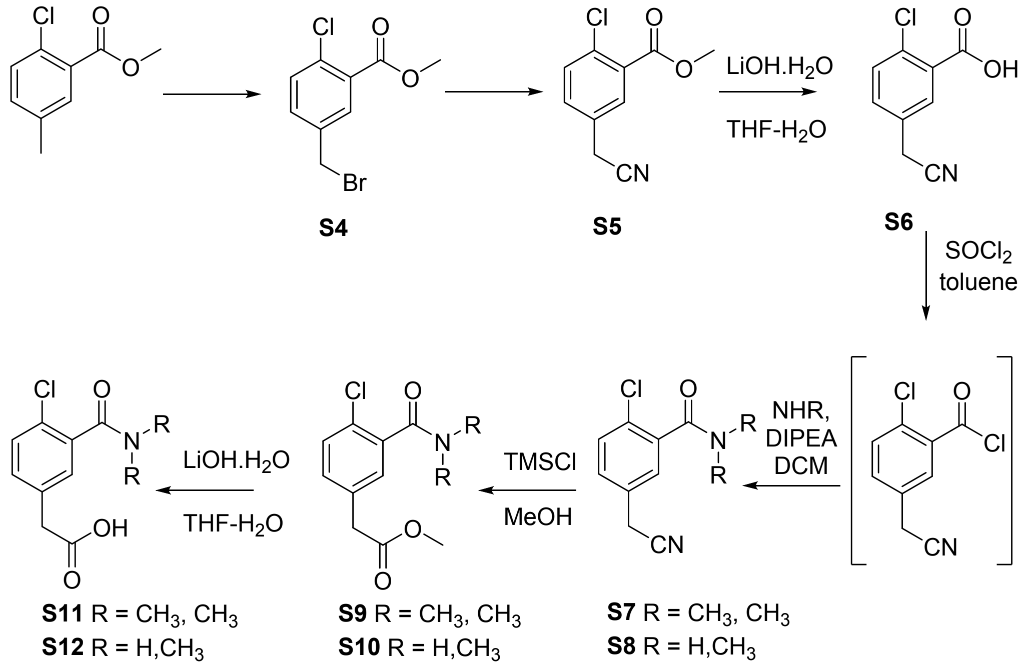


*Methyl 5-(bromomethyl)-2-chlorobenzoate (intermediate compound)* **S4**

Title compound was prepared according to the General procedure SI-I. The product was used as crude for the next step.[1]

*Methyl 2-chloro-5-(cyanomethyl)benzoate (intermediate compound)* **S5**

Methyl 5-(bromomethyl)-2-chlorobenzoate (1 g, 3.54 mmol) was dissolved in dry $CH_3CN$ and the mixture was degassed and refilled with argon. $K_2CO_3$ (540 mg, 1.1 eq) was added in one portion followed by TMSCN (0.66 mL, 1.5 eq). The reaction mixture was stirred at 60 °C for 7 hours (Scheme SI-1). After cooling to r.t. the mixture was quenched with 1 M NaOH, extracted with EtOAc and dried over sodium sulfate. Residue was purified by flash column chromatography, mobile phase petrolether/EtOAc (15-60 %). Yield 415 mg (56 %) as oil. $^{1}$H NMR (401 MHz, DMSO-$d_6$) δ 7.83 – 7.81 (m, 1H), 7.63 (d, $J$ = 8.4 Hz, 1H), 7.57 (ddt, $J$ = 8.3, 2.3, 0.7 Hz, 1H), 4.14 (t, $J$ = 0.7 Hz, 2H), 3.88 (s, 3H). $^{13}$C NMR (101 MHz, DMSO) δ 165.50, 133.30, 131.85, 131.61, 131.45, 131.08, 130.72, 119.14, 53.16, 22.12. HRMS: calcd for [M + H], 210.03163; found, 210.03151.

**General procedure SI-II. Ester hydrolysis**

Methyl 2-chloro-5-(cyanomethyl)benzoate was dissolved in THF/$H_2O$ 2:1 and LiOH.$H_2O$ (4 eq) was added in one portion. Reaction was stirred at r.t. and monitored by TLC. After the completion of the reaction, the mixture was extracted with EtOAc, water phase was acidified to pH 2 and extracted again with EtOAc. Organic phase was dried over sodium sulfate and purified by reverse-phase flash column chromatography.

*2-Chloro-5-(cyanomethyl)benzoic acid (intermediate compound)* **S6**

Title compound was prepared according to the General procedure SI-II (Scheme SI-1). Mobile phase $H_2O/CH_3CN$ 10-70 %. $^1$H NMR (401 MHz, DMSO-$d_6$) δ 13.16 (s, 3H), 7.78 (d, $J$ = 2.3 Hz, 1H), 7.58 (d, $J$ = 8.2 Hz, 1H), 7.51 (dd, $J$ = 8.3, 2.3 Hz, 1H), 4.11 (s, 2H). HRMS: calcd for [M - H], 194.00143; found, 194.00129.

**General procedure-SI-III. Amide preparation**

Carboxylic acid derivative (0.5 mmol) was placed in a round bottom flask and toluene was added (5 ml) followed by an addition of thionyl chloride (0.5 ml, in excess). The reaction mixture was stirred at 90 °C overnight. The reaction mixture was evaporated to dryness, co-evaporated with toluene and used directly for the next step without any purification. Acyl chloride derivative was dissolved in dry DCM and cooled in an ice bath. An appropriate amine (1.2 eq) was added followed by an addition of DIPEA (1.5 eq or 2 eq in case of amine salts). Reaction mixture was stirred at r.t. and monitored by TLC or LCMS. After completion of the reaction, the mixture was diluted with DCM, washed with water and purified by reverse phase flash CC or flash column chromatography.

*2-Chloro-5-(cyanomethyl)-N,N-dimethylbenzamide (intermediate compound)* **S7**

Title compound was prepared according to the General Procedure SI-III (Scheme SI-1). Mobile phase $H_2O/CH_3CN$ (10-70 %). Yield: 125 mg (86 %). $^1$H NMR (401 MHz, DMSO-$d_6$) δ 7.56 (d, $J$ = 8.3 Hz, 1H), 7.48 – 7.39 (m, 1H), 7.33 (d, $J$ = 2.2 Hz, 1H), 4.08 (s, 2H), 3.01 (s, 3H), 2.77 (s, 3H). $^{13}$C NMR (101 MHz, DMSO) δ 166.66, 136.91, 131.34, 130.23, 130.10, 128.50, 127.71, 118.97, 37.67, 34.21, 21.93. HRMS: calcd for [M + H], 223.06327; found, 223.06327.

*2-Chloro-5-(cyanomethyl)-N-methylbenzamide (intermediate compound)* **S8**

Title compound was prepared according to the General Procedure SI-III (Scheme SI-1). Mobile phase $H_2O/CH_3CN$ (10-70 %). Yield: 235 mg (91 %). $^1$H NMR (401 MHz, DMSO-$d_6$) δ 8.41 (q, $J$ = 4.7 Hz, 1H), 7.53 (dd, $J$ = 8.0, 0.7 Hz, 1H), 7.47 – 7.33 (m, 2H), 4.07 (t, $J$ = 0.7 Hz, 2H), 2.75 (d, $J$ = 4.6 Hz, 3H). $^{13}$C NMR (101 MHz, DMSO) δ 166.70, 137.91, 130.98, 130.81, 130.62, 129.59, 129.01, 119.26, 26.85, 22.11. HRMS: calcd for [M + H], 209.04762; found, 209.04754.

**General procedure SI-IV. Esterification of cyano group**

Cyanomethyl benzamide derivative was dissolved in small amount of dry MeOH and TMSCl (2eq) was added. A reaction mixture was stirred at 50 °C for 4 h and then ar r.t. overnight. The reaction mixture was diluted with water and extracted with EtOAc. Combined organic phases were dried over sodium sulfate and purified by reverse-phase column chromatography (Scheme 9).

*Methyl 2-(4-chloro-3-(dimethylcarbamoyl)phenyl)acetate (intermediate compound)* **S9**

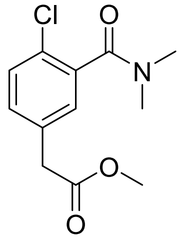

Title compound was prepared according to the General Procedure SI-IV (Scheme SI-1). Mobile phase $H_2O/CH_3CN$ (10-70 %). Yield: 110 mg (97 %). $^1$H NMR (401 MHz, DMSO-$d_6$) δ 7.47 (d, $J$ = 8.3 Hz, 1H), 7.33 (dd, $J$ = 8.3, 2.2 Hz, 1H), 7.25 (d, $J$ = 2.1 Hz, 1H), 3.74 (s, 2H), 3.62 (s, 2H), 3.00 (s, 2H), 2.76 (s, 2H). $^{13}$C NMR (101 MHz, DMSO) δ 171.65, 167.31, 136.55, 134.58, 131.89, 129.66, 129.31, 127.98, 52.30, 39.39, 37.96, 34.45. HRMS: calcd for [M + H], 256.07350; found, 256.07370.

*Methyl 2-(4-chloro-3-(methylcarbamoyl)phenyl)acetate (intermediate compound)* **S10**

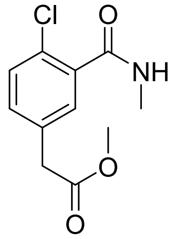

Title compound was prepared according to the General Procedure SI-IV (Scheme SI-1). Mobile phase $H_2O/CH_3CN$ (10-70 %). Yield: 118 mg (61 %). $^1$H NMR (401 MHz, DMSO-$d_6$) δ 8.35 (d, $J$ = 5.0 Hz, 1H), 7.46 – 7.43 (m, 1H), 7.35 – 7.31 (m, 2H), 3.74 (s, 2H), 3.63 (s, 3H), 2.75 (d, $J$ = 4.6 Hz, 3H).$^{13}$C NMR (101 MHz, DMSO) δ 171.64, 167.02, 137.40, 134.00, 132.17, 130.25, 129.91, 128.77, 52.29, 26.42. HRMS: calcd for [M + H], 242.05785; found, 242.05789.

*2-(4-Chloro-3-(dimethylcarbamoyl)phenyl)acetic acid (intermediate compound)* **S11**

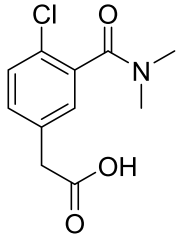

Title compound was prepared according to the General Procedure SI-II (Scheme SI-I). Mobile phase $H_2O/CH_3CN$ (10-70 %). Yield: 228 mg (97 %). $^1$H NMR (401 MHz, DMSO-$d_6$) δ 12.47 (s, 1H), 7.45 (d, $J$ = 8.3 Hz, 1H), 7.32 (dd, $J$ = 8.3, 2.2 Hz, 1H), 7.23 (d, $J$ = 2.2 Hz, 1H), 3.62 (s, 2H), 3.00 (s, 3H), 2.76 (s, 3H). $^{13}$C NMR (101 MHz, DMSO) δ 172.36, 167.09, 136.17, 134.98, 131.63, 129.24, 129.01, 127.44, 37.67, 34.16. dmso overlap HRMS: calcd for [M + H], 242.05785; found, 242.05764.

*2-(4-Chloro-3-(methylcarbamoyl)phenyl)acetic acid (intermediate compound)* **S12**

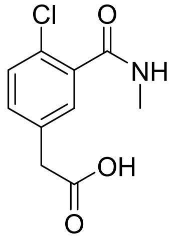

Title compound was prepared according to the General Procedure SI-II (Scheme SI-1). Mobile phase $H_2O/CH_3CN$ (10-70 %). Yield: 180 mg (97 %). $^1$H NMR (401 MHz, DMSO-$d_6$) δ 12.45 (s, 1H), 8.34 (q, $J$ = 4.6 Hz, 1H), 7.45 – 7.40 (m, 1H), 7.35 – 7.30 (m, 2H), 3.62 (s, 2H), 2.75 (d, $J$ = 4.6 Hz, 3H). $^{13}$C NMR (101 MHz, DMSO) δ 172.67, 167.13, 137.28, 134.66, 132.19, 130.24, 129.79, 128.54, 39.99, 26.42. HRMS: calcd for [M + Na], 250.02414; found, 250.02402.

Scheme SI S2 - Synthesis of methylated MI1013 - PROTAC **S15**

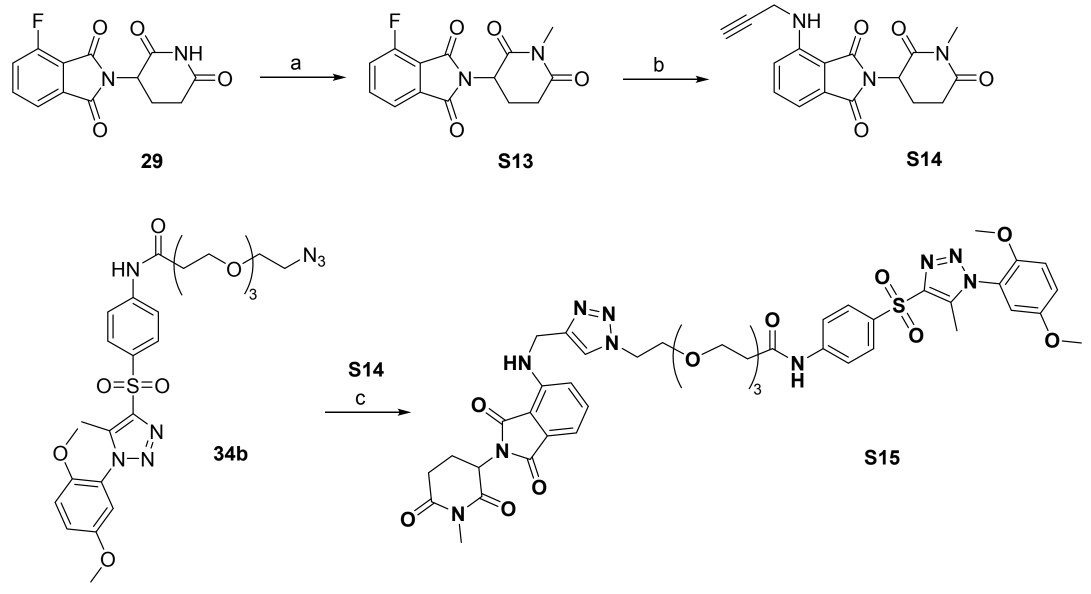

[a]Reagents and conditions: a) MeI, $Cs_2CO_3$, DMF, r.t., o/n, 90%; b) propargyl bromide, DIPEA, DMSO, 90 °C, 1 h, 65 %; c) **S14**, $CuSO_4.5H_2O$, Na-ascorbate, THF/$H_2O$ 1:1, 3 h, 87 %.

*4-Fluoro-2-(1-methyl-2,6-dioxopiperidin-3-yl)isoindoline-1,3-dione* **S13**

2-(2,6-dioxopiperidin-3-yl)-4-fluoroisoindoline-1,3-dione **29** (0.25 g, 1 eq) was dissolved in dry DMF (4mL) and $Cs_2CO_3$ (0.44g, 1.5 eq) was added. The mixture was stirred at r.t. for 10 min and MeI (62 mL) was added. The mixture was stirred overnight. After completion o fthe reaction, the was diluted with EtOAc, washed with water, dried over sodium sulfate and the residue was purified by column chromatography, mobile phase EtOAc/hexane (1:1). Yield 236mg (90 %).

$^1$H NMR (500 MHz, DMSO) δ 7.95 (ddd, *J* = 8.4, 7.4, 4.4 Hz, 1H), 7.79 (d, *J* = 7.3 Hz, 1H), 7.74 (t, *J* = 8.9 Hz, 1H), 5.22 (dd, *J* = 13.1, 5.4 Hz, 1H), 3.02 (s, 3H), 2.96 (ddd, *J* = 17.2, 13.9, 5.5 Hz, 1H), 2.78 (ddd, *J* = 17.3, 4.5, 2.5 Hz, 1H), 2.55 (td, *J* = 13.3, 4.6 Hz, 1H), 2.08 (dtd, *J* = 13.0, 5.4, 2.5 Hz, 1H). $^{13}$C NMR (126 MHz, DMSO) δ 171.87, 169.59, 166.25 (d, *J* = 2.8 Hz), 164.12, 158.04, 155.96, 138.25 (d, *J* = 7.8 Hz), 133.60, 123.20 (d, *J* = 19.7 Hz), 120.23 (d, *J* = 3.2 Hz), 117.19 (d, *J* = 12.5 Hz), 49.83, 31.22, 26.80, 21.20. HRMS: calcd for [M + H], 291.0776, found 291.0782.

*2-(1-Methyl-2,6-dioxopiperidin-3-yl)-4-(prop-2-yn-1-ylamino)isoindoline-1,3-dione* **S14**

4-Fluoro-2-(1-methyl-2,6-dioxopiperidin-3-yl)isoindoline-1,3-dione **S13** (0.22 g, 1 eq) was dissolved in dry DMSO (4.5 ml) and propargyl amine (60 mL, 1.2 eq) was added followed by an addition of DIPEA (0.42 ml, 3 eq) and the mixture was stirred at 90 °C for 1 h, when the TLC indicated completion of the reaction. The mixture was diluted with water and extracted with EtOAc. Organic phase was washed with brine and dried over sodium sulfate. The crude was purified by column chromatography, mobile phase hexane/EtOAc (4:1) - EtOAc. Yield 160 mg of yellow foam (65 %). $^1$H NMR (500 MHz,

DMSO) δ 7.66 (dd, *J* = 8.5, 7.1 Hz, 1H), 7.16 (d, *J* = 8.5 Hz, 1H), 7.12 (d, *J* = 7.1 Hz, 1H), 6.93 (t, *J* = 6.2 Hz, 1H), 5.13 (dd, *J* = 13.0, 5.4 Hz, 1H), 4.17 (dd, *J* = 6.3, 2.5 Hz, 2H), 3.17 (t, *J* = 2.4 Hz, 1H), 3.02 (s, 3H), 2.95 (ddd, *J* = 17.2, 13.9, 5.4 Hz, 1H), 2.76 (ddd, *J* = 17.2, 4.5, 2.6 Hz, 1H), 2.54 (dd, *J* = 13.3, 8.8 Hz, 1H), 2.05 (dtd, *J* = 13.0, 5.3, 2.5 Hz, 1H). $^{13}$C NMR (126 MHz, DMSO) δ 171.99, 169.98, 168.75, 167.39, 145.39, 136.28, 132.32, 118.17, 111.57, 110.42, 81.07, 73.95, 59.93, 49.33, 31.69, 31.28, 26.77, 21.52. HRMS: calcd for [M + H], 326.1135, found 326.1138.

*N-(4-((1-(2,5-dimethoxyphenyl)-5-methyl-1H-1,2,3-triazol-4-yl)sulfonyl)phenyl)-3-(2-(2-(2-(4-(((2-(1-methyl-2,6-dioxopiperidin-3-yl)-1,3-dioxoisoindolin-4-yl)amino)methyl)-1H-1,2,3-triazol-1-yl)ethoxy)ethoxy)ethoxy)propenamide* **S15**

3-(2-(2-(2-azidoethoxy)ethoxy)ethoxy)-N-(4-((1-(2,5-dimethoxyphenyl)-5-methyl-1H-1,2,3-triazol-4-yl)sulfonyl)phenyl)propenamide (**34b**) (100 mg, 1 eq) was dissolved in THF/water 1:1 mixture and degassed at 0 °C and refilled with argon. 2-(1-Methyl-2,6-dioxopiperidin-3-yl)-4-(prop-2-yn-1-ylamino)isoindoline-1,3-dione **S14** (54.0 mg, 1 eq) was added together with Na-ascorbate (33 mg, 1 eq) and $CuSO_4.5H_2O$ (4 mg, 0.05 eq). The mixture was degassed again and stirred at r.t. for 3 h. Completion of the reaction monitored by TLC. After the completion the mixture was diluted with EtOAc, washed with water, dried over sodium sulfate and the residue purified by column chromatography, mobile phase EtOAc/MeOH (10:1). Yield 133 mg (87 %).

$^{1}$H NMR (500 MHz, DMSO) δ 10.43 (s, 1H), 8.00 – 7.92 (m, 3H), 7.88 – 7.82 (m, 2H), 7.56 (dd, J = 8.5, 7.1 Hz, 1H), 7.26 (d, J = 9.1 Hz, 1H), 7.22 – 7.15 (m, 3H), 7.05 (dd, J = 6.7, 2.0 Hz, 2H), 5.75 (s, 1H), 5.12 (dd, J = 13.0, 5.4 Hz, 1H), 4.58 (d, J = 6.1 Hz, 2H), 4.45 (t, J = 5.2 Hz, 2H), 3.77 – 3.70 (m, 8H), 3.68 (t, J = 6.1 Hz, 2H), 3.48 – 3.39 (m, 8H), 3.01 (s, 3H), 2.94 (ddd, J = 17.1, 13.9, 5.4 Hz, 1H), 2.75 (ddd, J = 17.2, 4.5, 2.6 Hz, 1H), 2.58 (q, J = 6.5 Hz, 2H), 2.04 (dtd, J = 13.1, 5.4, 2.6 Hz, 1H). $^{13}$C NMR (126 MHz, DMSO) δ 171.96, 170.38, 169.97, 168.93, 167.41, 153.23, 147.77, 146.03, 144.48, 144.33, 143.53, 138.59, 136.32, 134.25, 132.27, 128.90, 123.42, 123.16, 119.24, 118.18, 117.82, 114.09, 114.07, 111.12, 109.80, 69.83, 69.78, 69.76, 69.67, 68.83, 66.54, 59.93, 56.53, 56.05, 55.08, 49.53, 49.30, 37.43, 31.28, 26.76, 21.54, 8.72. HRMS: calcd for [M + H], 929.3247, found 929.3234.

**SI-NMR**

**Cmp 2**

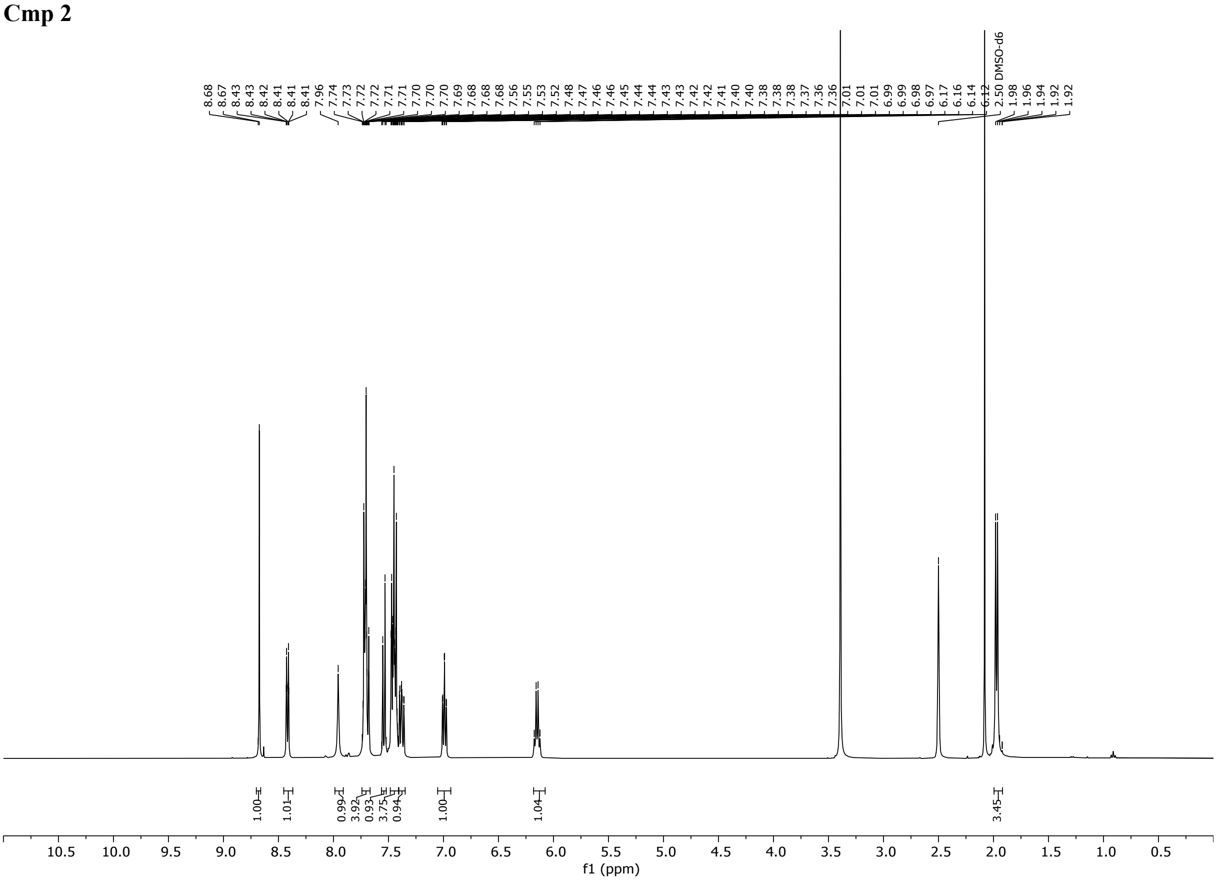

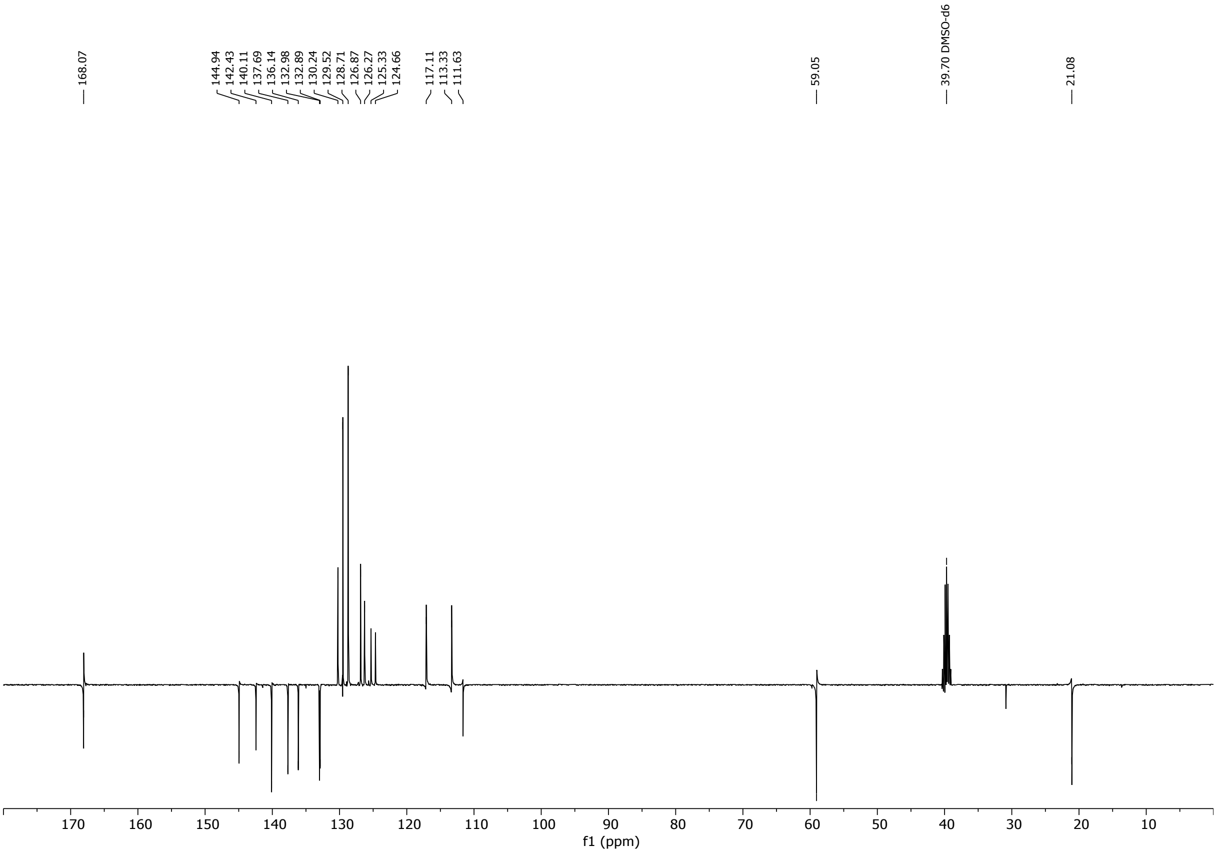

**Cmp 3**

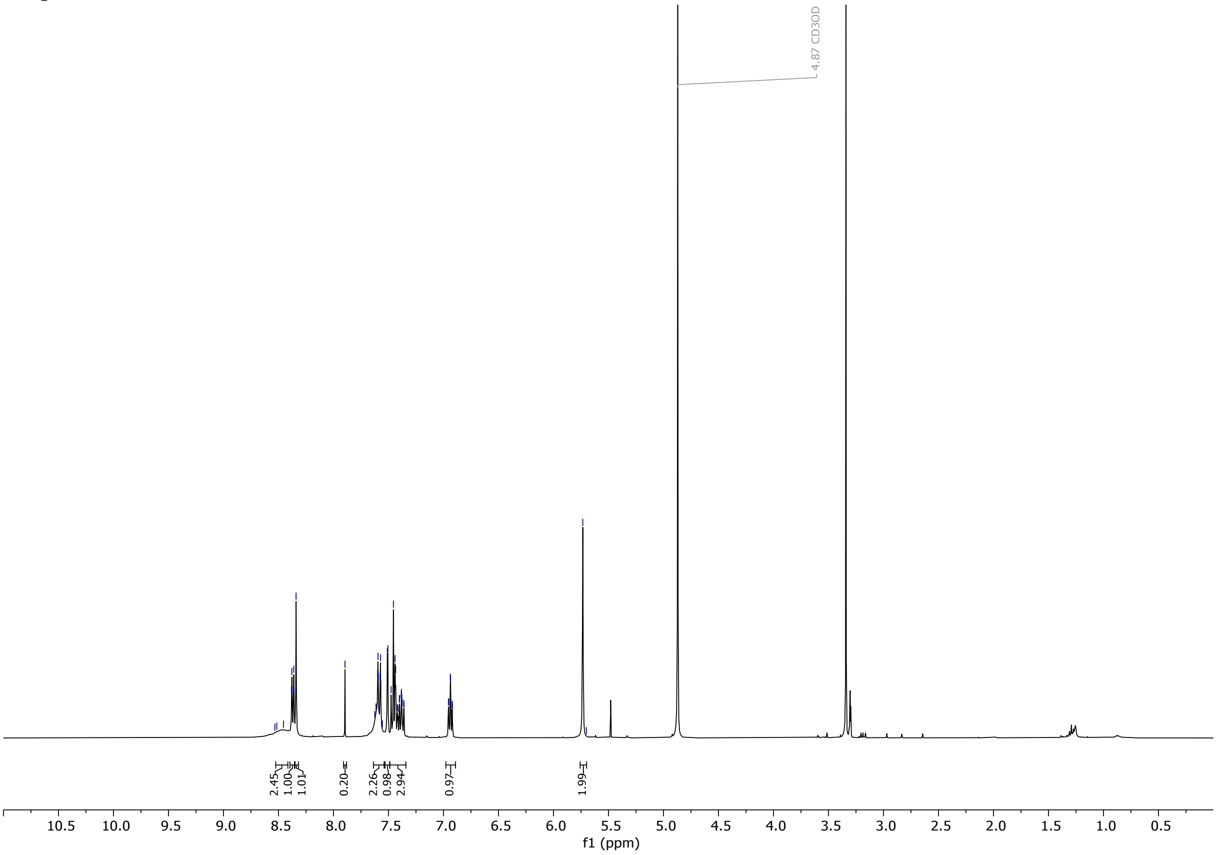
4.87 CD3OD
2.45
1.00
1.01
0.20
2.26
0.98
2.94
0.97
1.99
10.5 10.0 9.5 9.0 8.5 8.0 7.5 7.0 6.5 6.0 5.5 5.0 4.5 4.0 3.5 3.0 2.5 2.0 1.5 1.0 0.5
f1 (ppm)

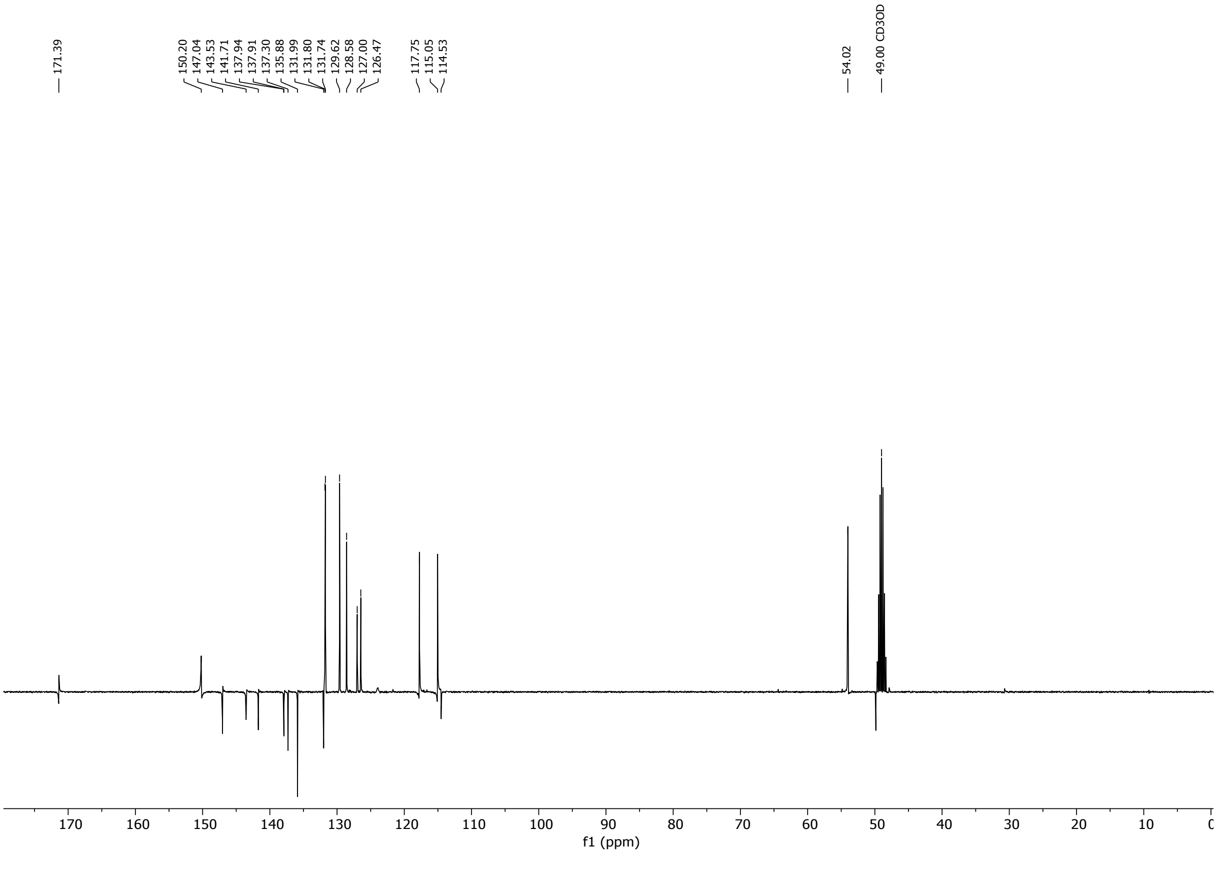
171.39
150.20
147.04
143.53
141.71
137.94
137.91
137.30
135.88
131.99
131.80
131.74
129.62
128.58
127.00
126.47
117.75
115.05
114.53
54.02
49.00 CD3OD
170 160 150 140 130 120 110 100 90 80 70 60 50 40 30 20 10
f1 (ppm)

**Cmp 4**

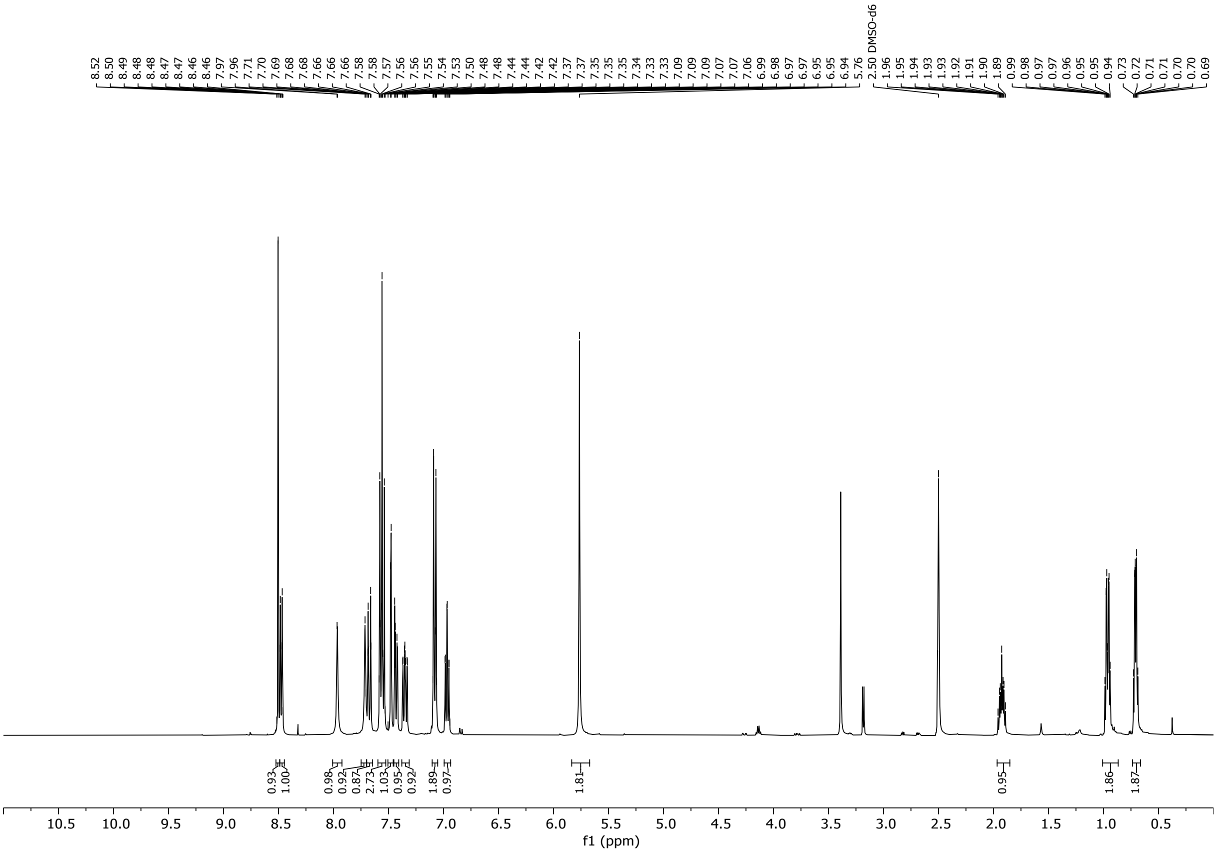
8.52 8.50 8.49 8.48 8.48 8.47 8.47 8.46 8.46 7.97 7.96 7.71 7.70 7.69 7.68 7.68 7.66 7.66 7.58 7.58 7.57 7.56 7.56 7.55 7.54 7.53 7.50 7.48 7.48 7.44 7.44 7.42 7.42 7.37 7.37 7.35 7.35 7.34 7.33 7.33 7.09 7.09 7.09 7.07 7.06 6.99 6.98 6.97 6.97 6.95 6.95 6.94 5.76
2.50 DMSO-d6
1.96 1.95 1.94 1.93 1.93 1.92 1.91 1.90 1.89 0.99 0.98 0.97 0.97 0.96 0.95 0.95 0.94 0.73 0.72 0.71 0.71 0.70 0.70 0.69
0.93 1.00 0.98 0.92 0.87 2.73 1.03 0.95 0.92 1.89 0.97 1.81 0.95 1.86 1.87
10.5 10.0 9.5 9.0 8.5 8.0 7.5 7.0 6.5 6.0 5.5 5.0 4.5 4.0 3.5 3.0 2.5 2.0 1.5 1.0 0.5
f1 (ppm)

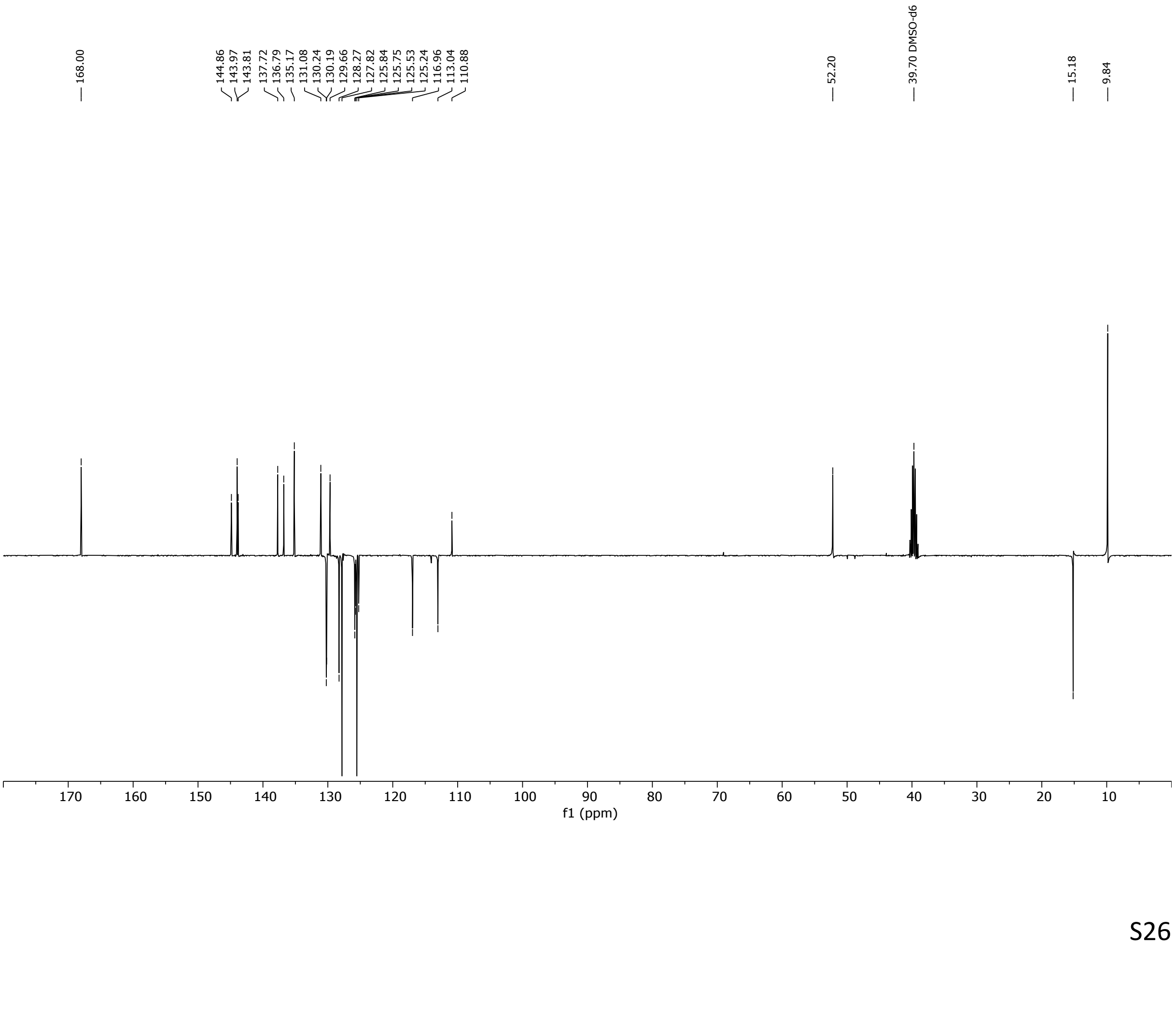
168.00
144.86 143.97 143.81 137.72 136.79 135.17 131.08 130.24 130.19 129.66 128.27 127.82 125.84 125.75 125.53 125.24 116.96 113.04 110.88
52.20
39.70 DMSO-d6
15.18
9.84
170 160 150 140 130 120 110 100 90 80 70 60 50 40 30 20 10
f1 (ppm)

**Cmp 5**

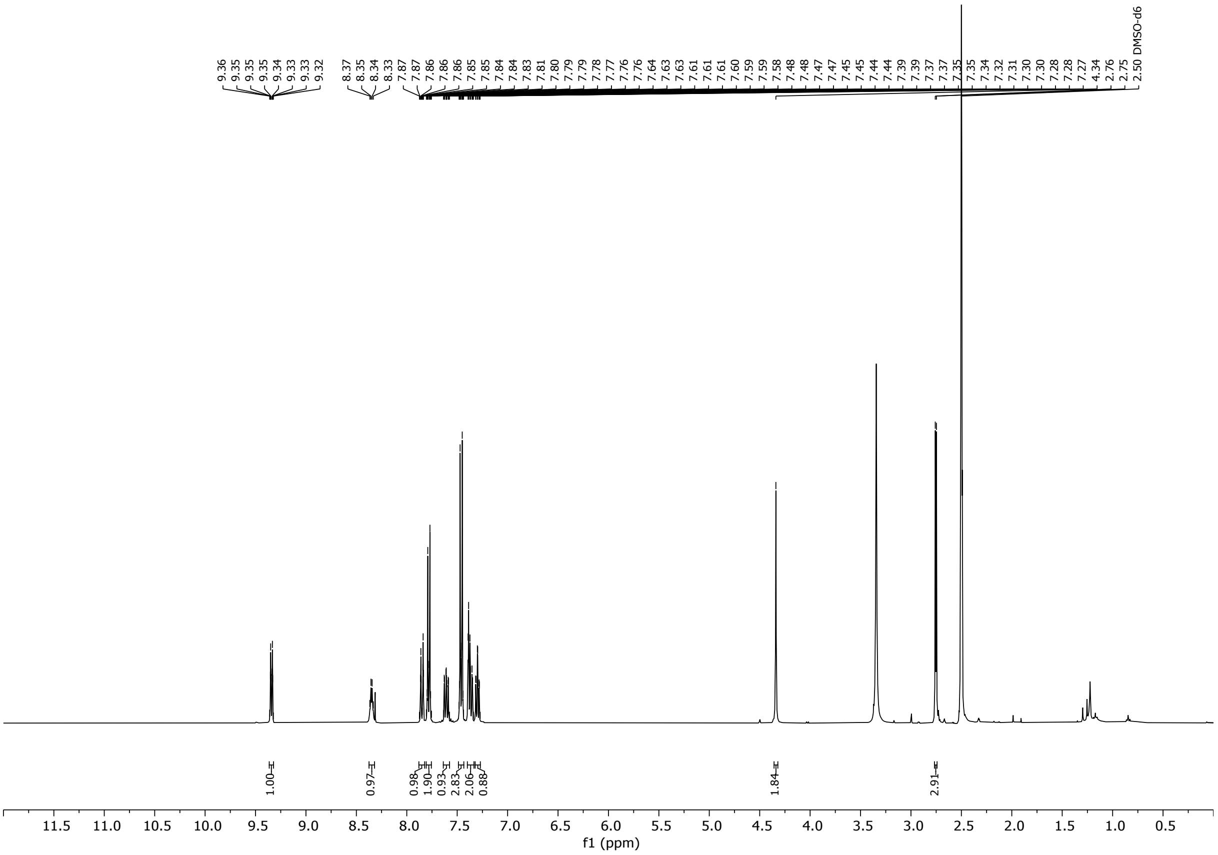

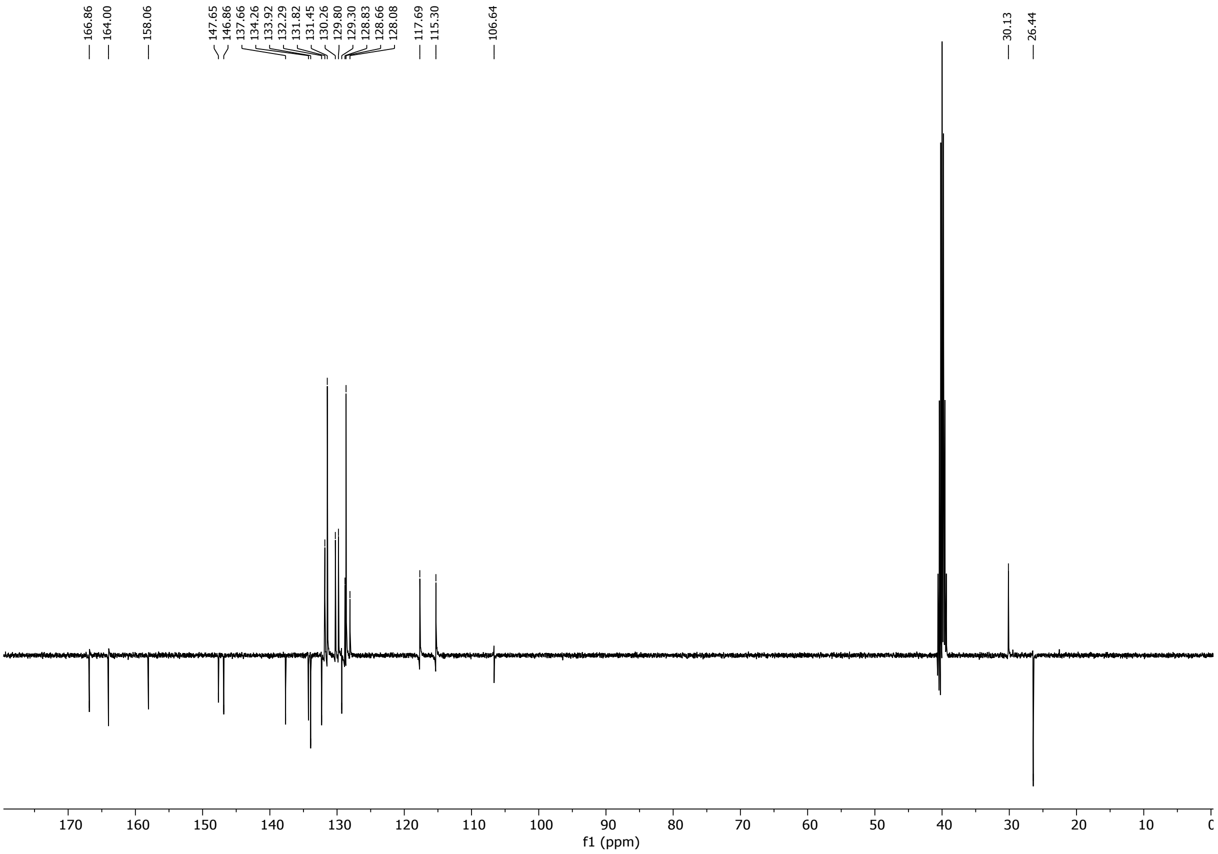

**Cmp 6**

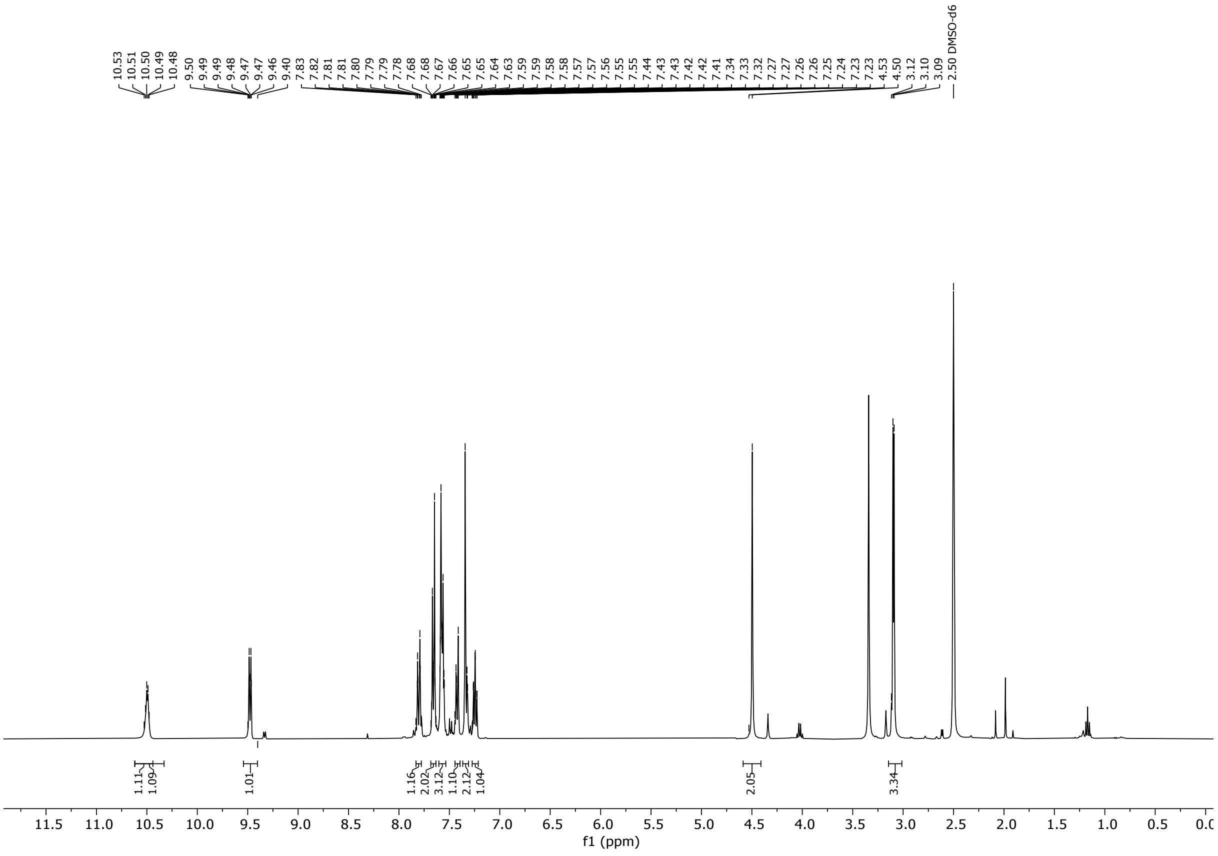
10.53
10.51
10.50
10.49
10.48
9.50
9.49
9.49
9.48
9.47
9.47
9.46
9.40
7.83
7.82
7.81
7.81
7.80
7.79
7.79
7.78
7.68
7.67
7.66
7.65
7.65
7.64
7.63
7.59
7.59
7.58
7.58
7.57
7.57
7.56
7.55
7.55
7.44
7.43
7.43
7.42
7.42
7.41
7.34
7.33
7.32
7.27
7.27
7.26
7.26
7.25
7.24
7.23
7.23
4.53
4.50
3.12
3.10
3.09
2.50 DMSO-d6
1.11
1.09
1.01
1.16
2.02
3.12
1.10
2.12
1.04
2.05
3.34
11.5 11.0 10.5 10.0 9.5 9.0 8.5 8.0 7.5 7.0 6.5 6.0 5.5 5.0 4.5 4.0 3.5 3.0 2.5 2.0 1.5 1.0 0.5 0.0
f1 (ppm)

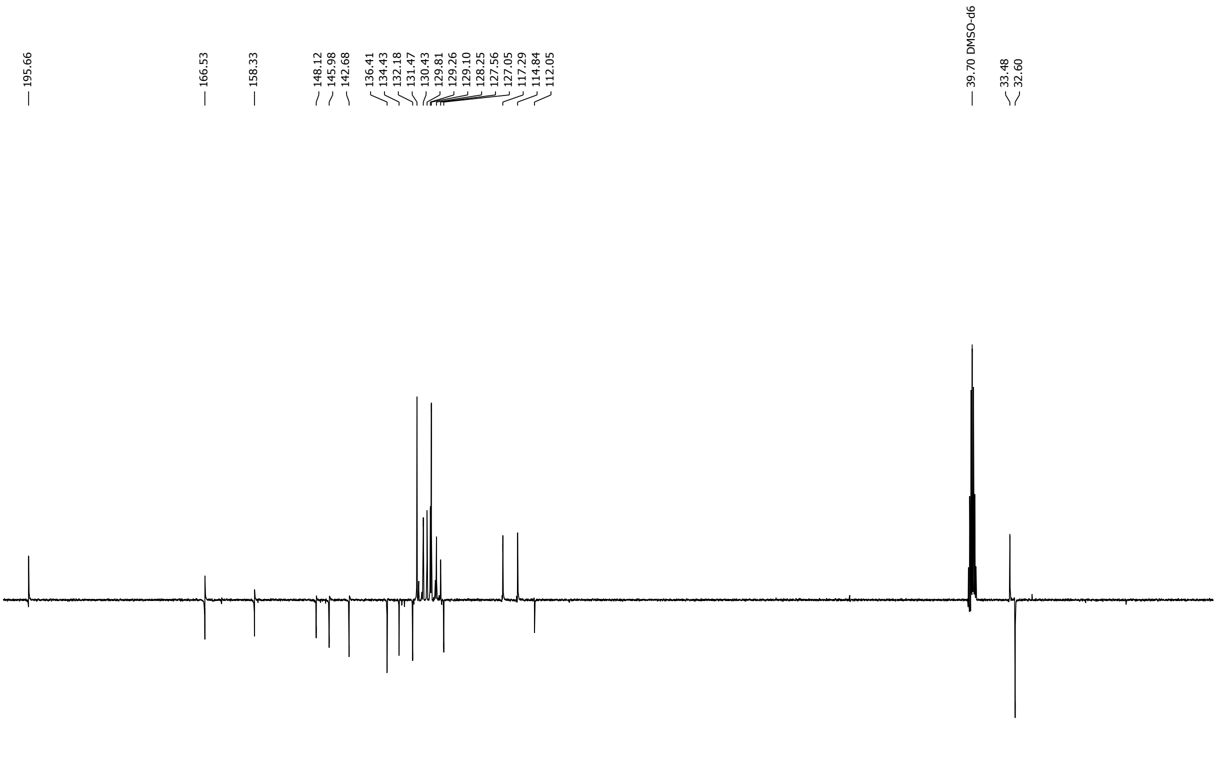
195.66
166.53
158.33
148.12
145.98
142.68
136.41
134.43
132.18
131.47
130.43
129.81
129.26
129.10
128.25
127.56
127.05
117.29
114.84
112.05
39.70 DMSO-d6
33.48
32.60
190 180 170 160 150 140 130 120 110 100 90 80 70 60 50 40 30 20 10
f1 (ppm)

**Cmp 7**

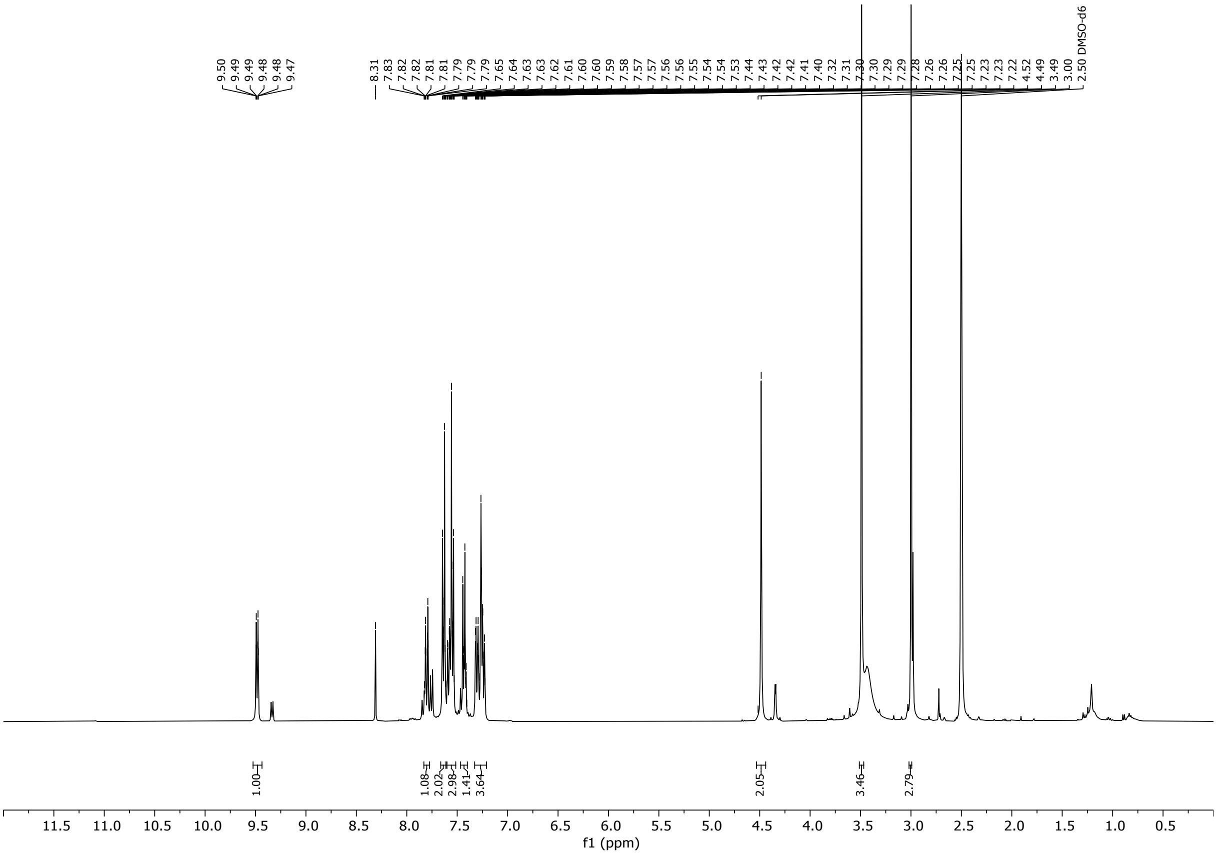
9.50
9.49
9.49
9.48
9.48
9.47
8.31
2.50 DMSO-d6
1.00
1.08
2.02
2.98
1.41
3.64
2.05
3.46
2.79
f1 (ppm)

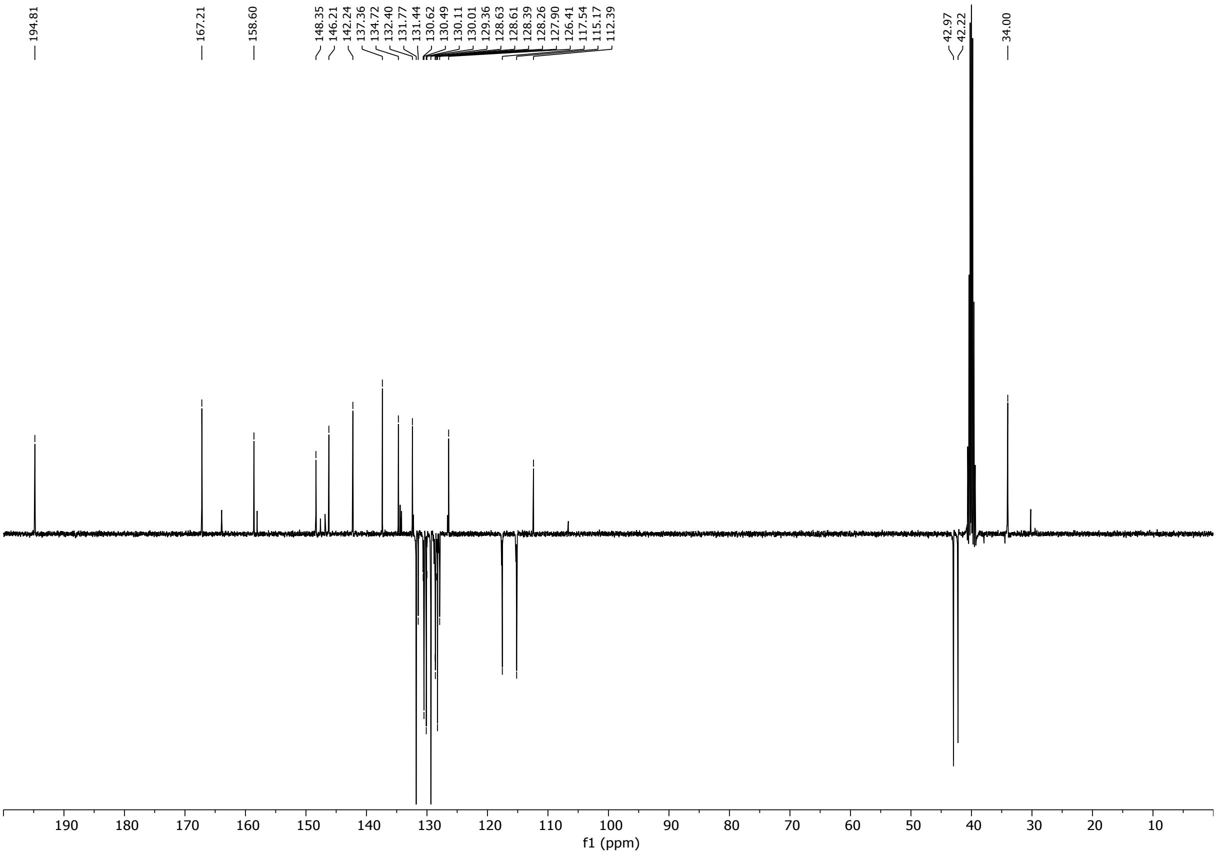
194.81
167.21
158.60
148.35
146.21
142.24
137.36
42.97
42.22
34.00
f1 (ppm)

**Cmp 8**

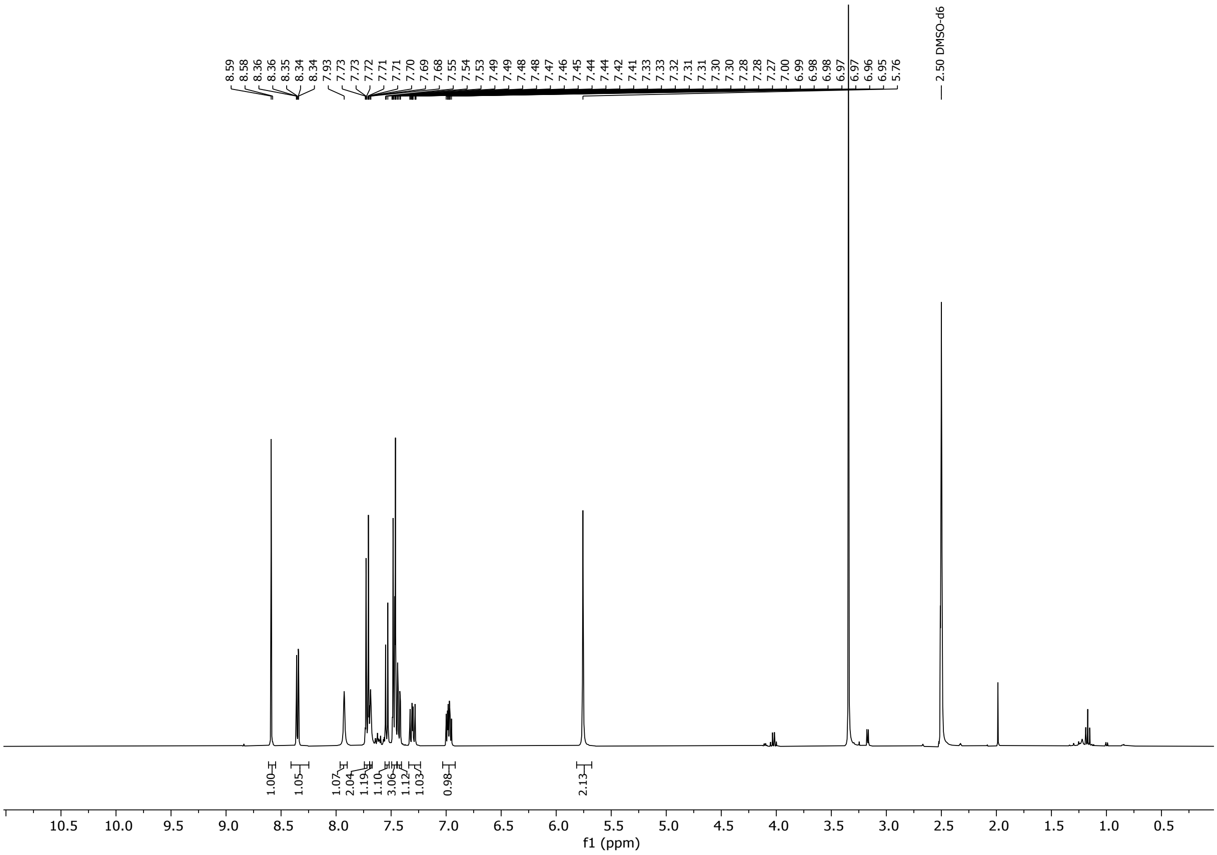
8.59 8.58 8.36 8.36 8.35 8.34 8.34 7.93 7.73 7.73 7.72 7.71 7.71 7.70 7.69 7.68 7.55 7.54 7.53 7.49 7.49 7.48 7.48 7.47 7.46 7.45 7.44 7.44 7.42 7.41 7.33 7.33 7.32 7.31 7.31 7.30 7.28 7.28 7.27 7.00 6.99 6.98 6.98 6.97 6.97 6.96 6.95 5.76
2.50 DMSO-d6
1.00 1.05 1.07 2.04 1.19 1.10 3.06 1.12 1.03 0.98 2.13
10.5 10.0 9.5 9.0 8.5 8.0 7.5 7.0 6.5 6.0 5.5 5.0 4.5 4.0 3.5 3.0 2.5 2.0 1.5 1.0 0.5
f1 (ppm)

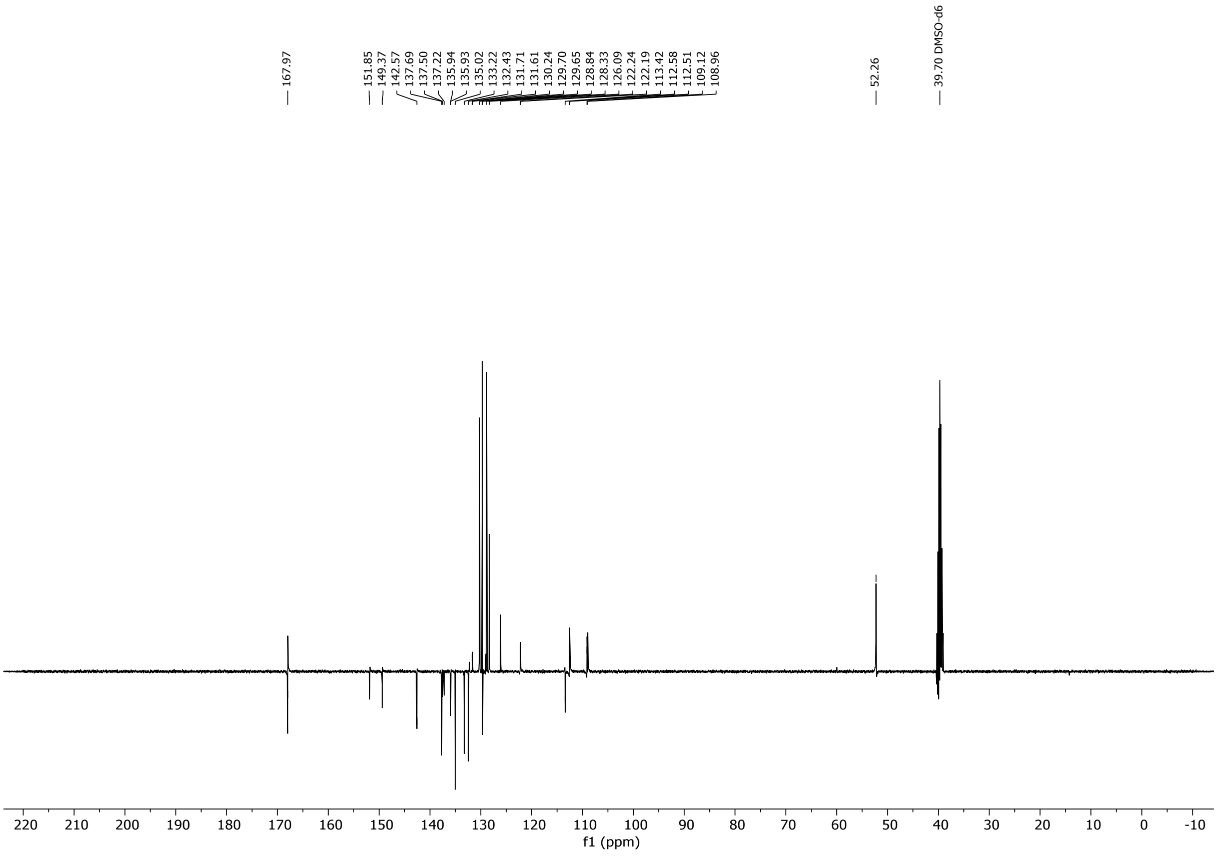
167.97
151.85 149.37 142.57 137.69 137.50 137.22 135.94 135.93 135.02 133.22 132.43 131.71 131.61 130.24 129.70 129.65 128.84 128.33 126.09 122.24 122.19 113.42 112.58 112.51 109.12 108.96
52.26
39.70 DMSO-d6
220 210 200 190 180 170 160 150 140 130 120 110 100 90 80 70 60 50 40 30 20 10 0 -10
f1 (ppm)

**Cmp 9**

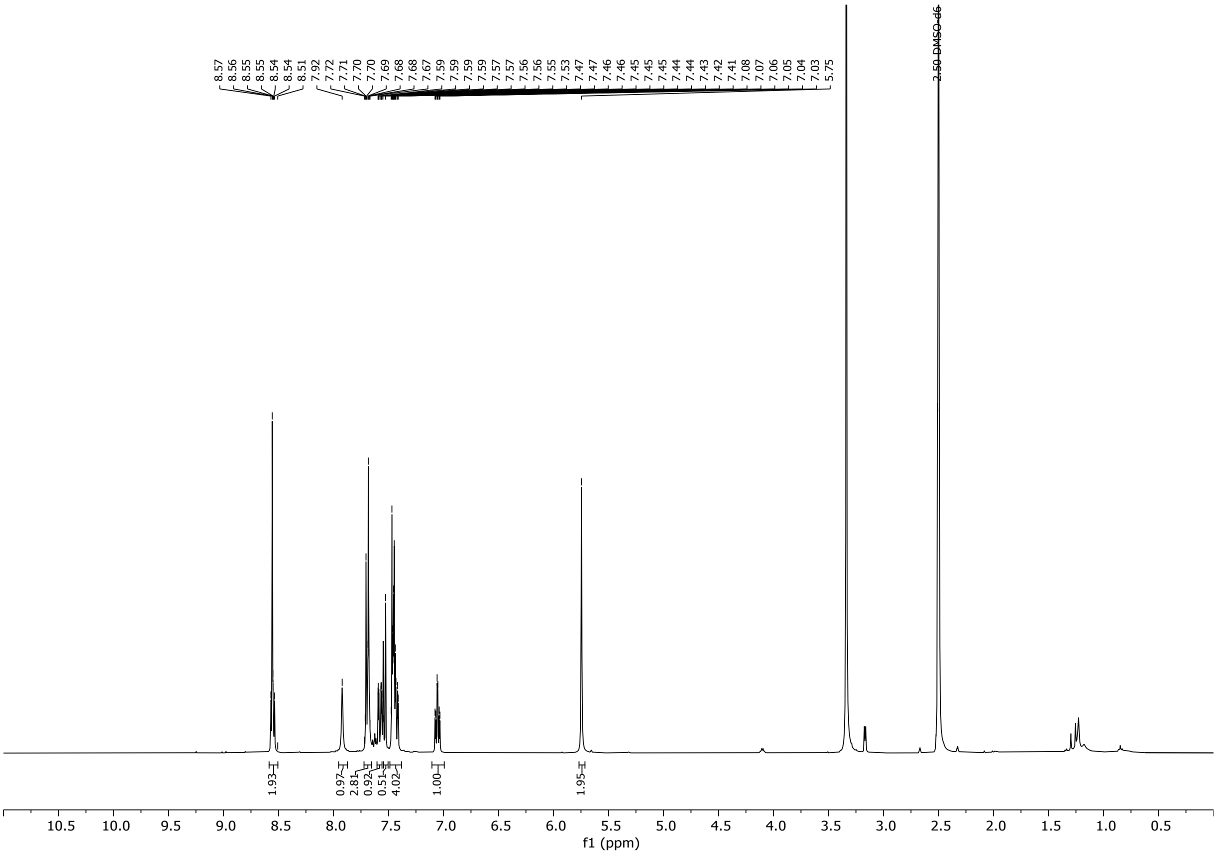

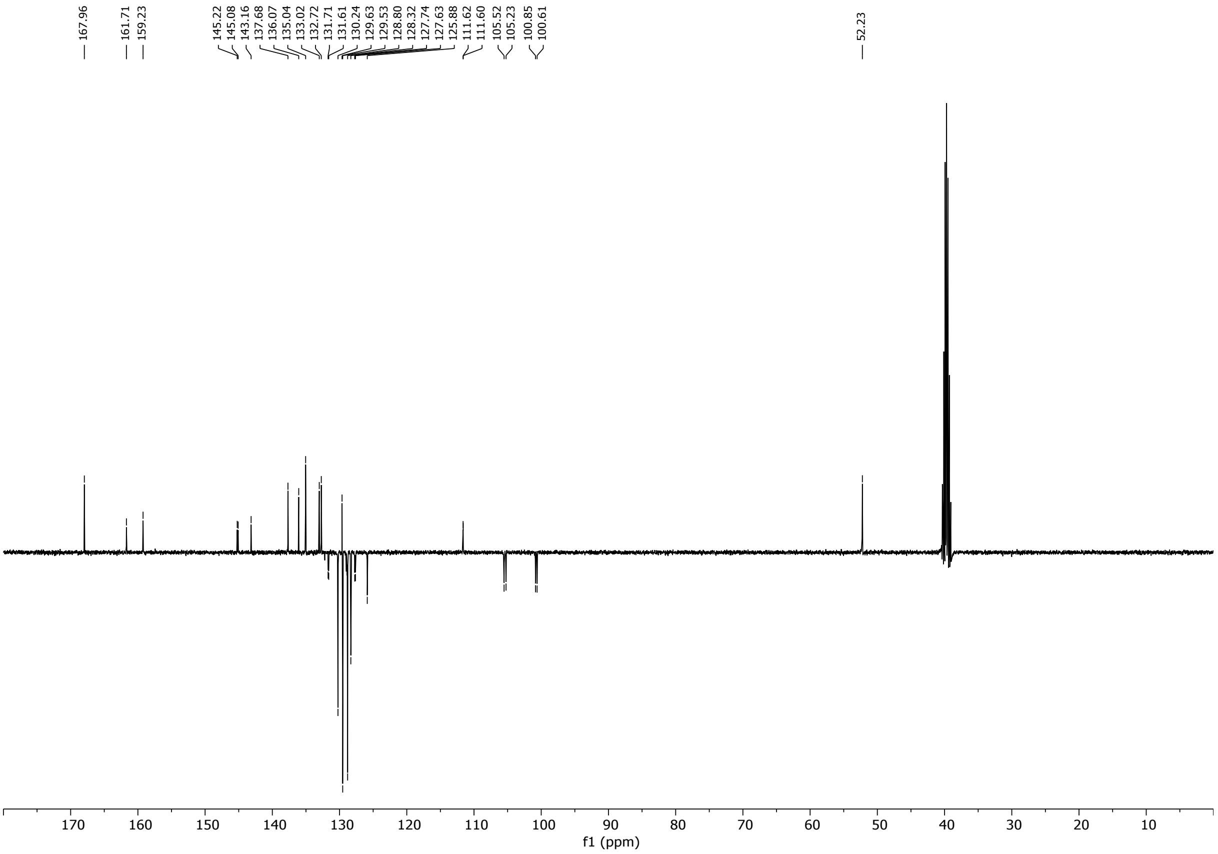

**Cmp 10**

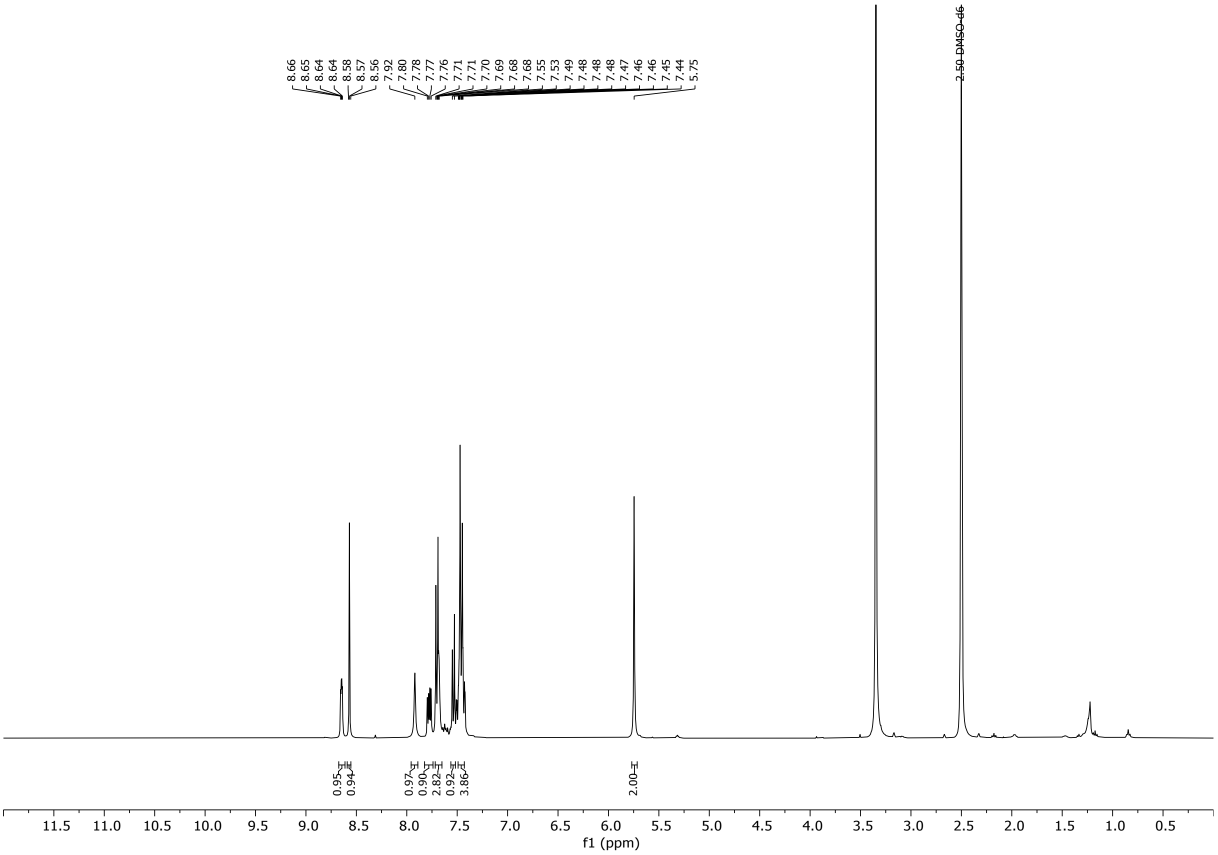

8.66 8.65 8.64 8.64 8.58 8.57 8.56 7.92 7.80 7.78 7.77 7.76 7.71 7.71 7.70 7.69 7.68 7.68 7.55 7.53 7.49 7.48 7.48 7.48 7.47 7.46 7.46 7.45 7.44 5.75
2.50 DMSO-d6
0.95 0.94 0.97 0.90 2.82 0.92 3.86 2.00
f1 (ppm)


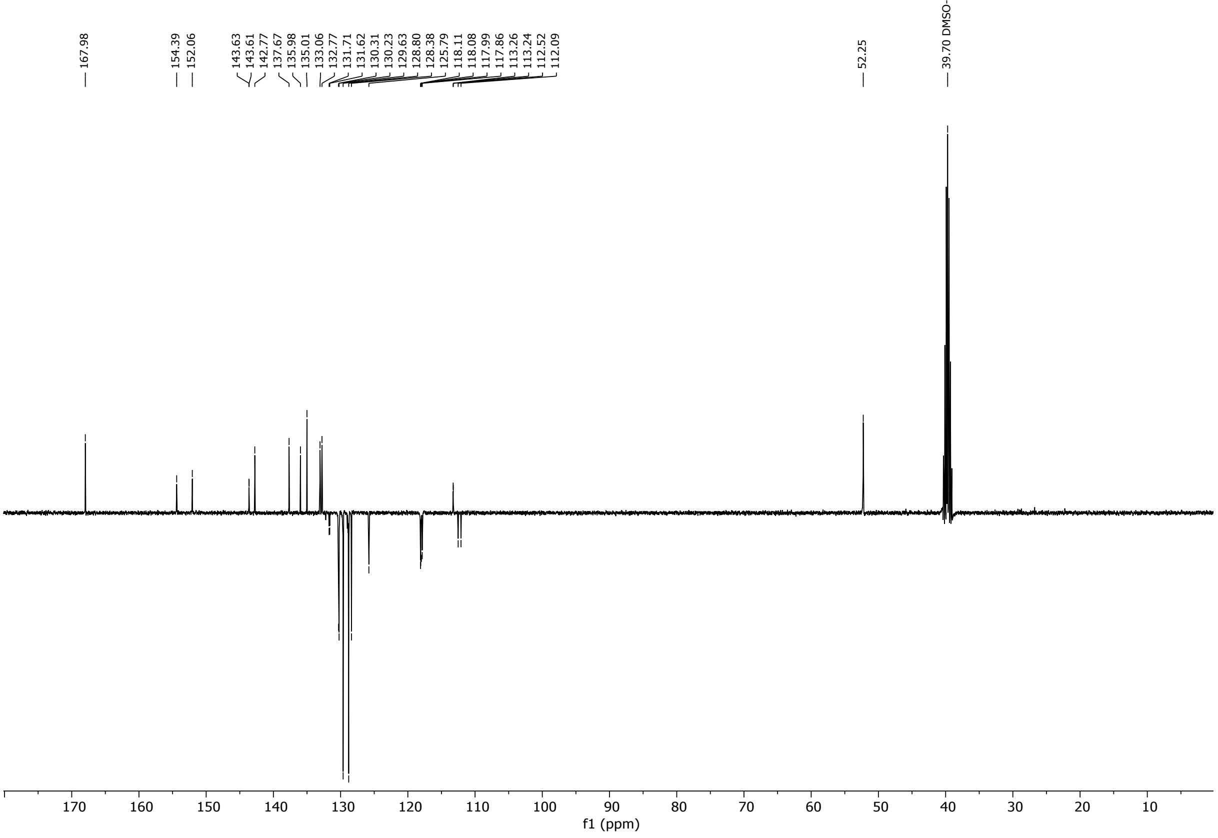

167.98
154.39 152.06
143.63 143.61 142.77 137.67 135.98 135.01 133.06 132.77 131.71 131.62 130.31 130.23 129.63 128.80 128.38 125.79 118.11 118.08 117.99 117.86 113.26 113.24 112.52 112.09
52.25
39.70 DMSO-d6
f1 (ppm)

**Cmp 11**

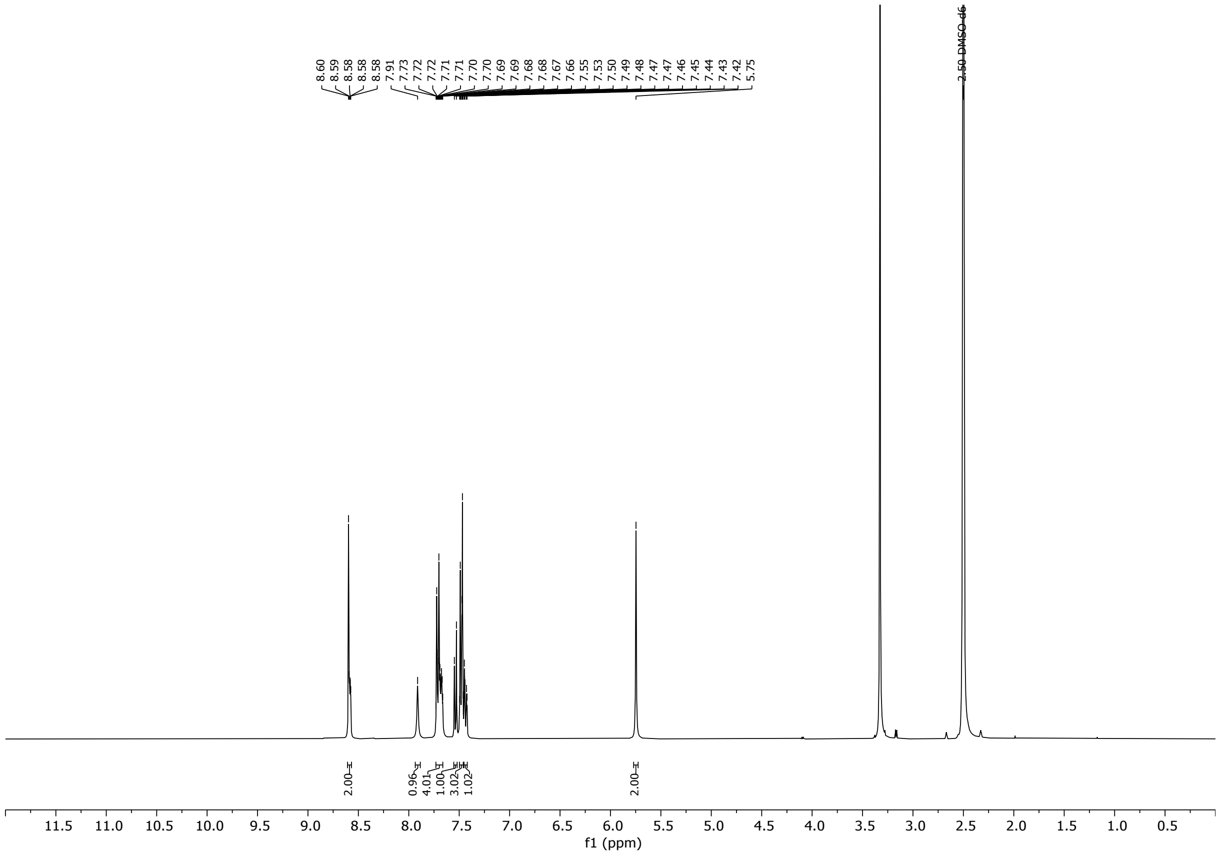
8.60
8.59
8.58
8.58
8.58
7.91
7.73
7.72
7.72
7.71
7.71
7.70
7.70
7.69
7.69
7.68
7.68
7.67
7.66
7.55
7.53
7.50
7.49
7.48
7.47
7.47
7.46
7.45
7.44
7.43
7.42
5.75
2.50 DMSO-d6
2.00
0.96
4.01
1.00
3.02
1.02
2.00
f1 (ppm)

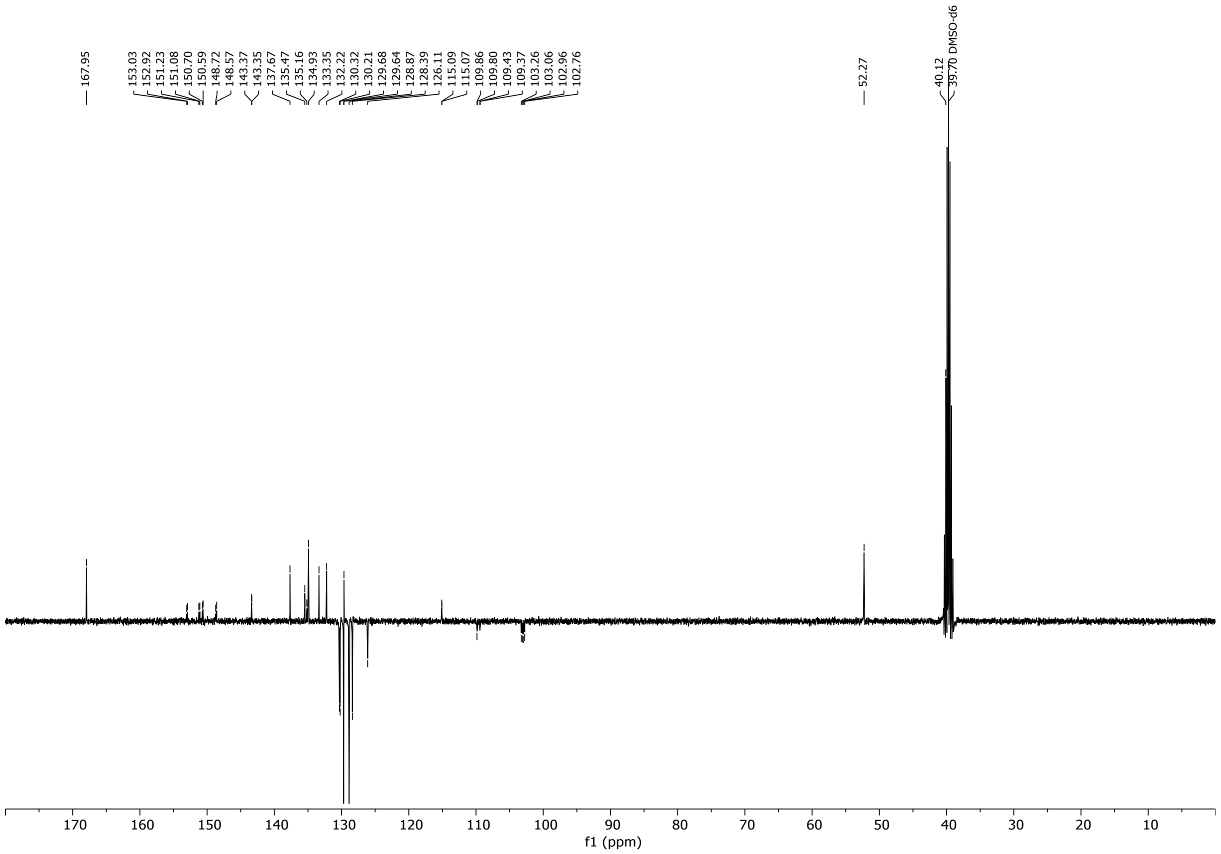
167.95
153.03
152.92
151.23
151.08
150.70
150.59
148.72
148.57
143.37
143.35
137.67
135.47
135.16
134.93
133.35
132.22
130.32
130.21
129.68
129.64
128.87
128.39
126.11
115.09
115.07
109.86
109.80
109.43
109.37
103.26
103.06
102.96
102.76
52.27
40.12
39.70 DMSO-d6
f1 (ppm)

**Cmp 12**

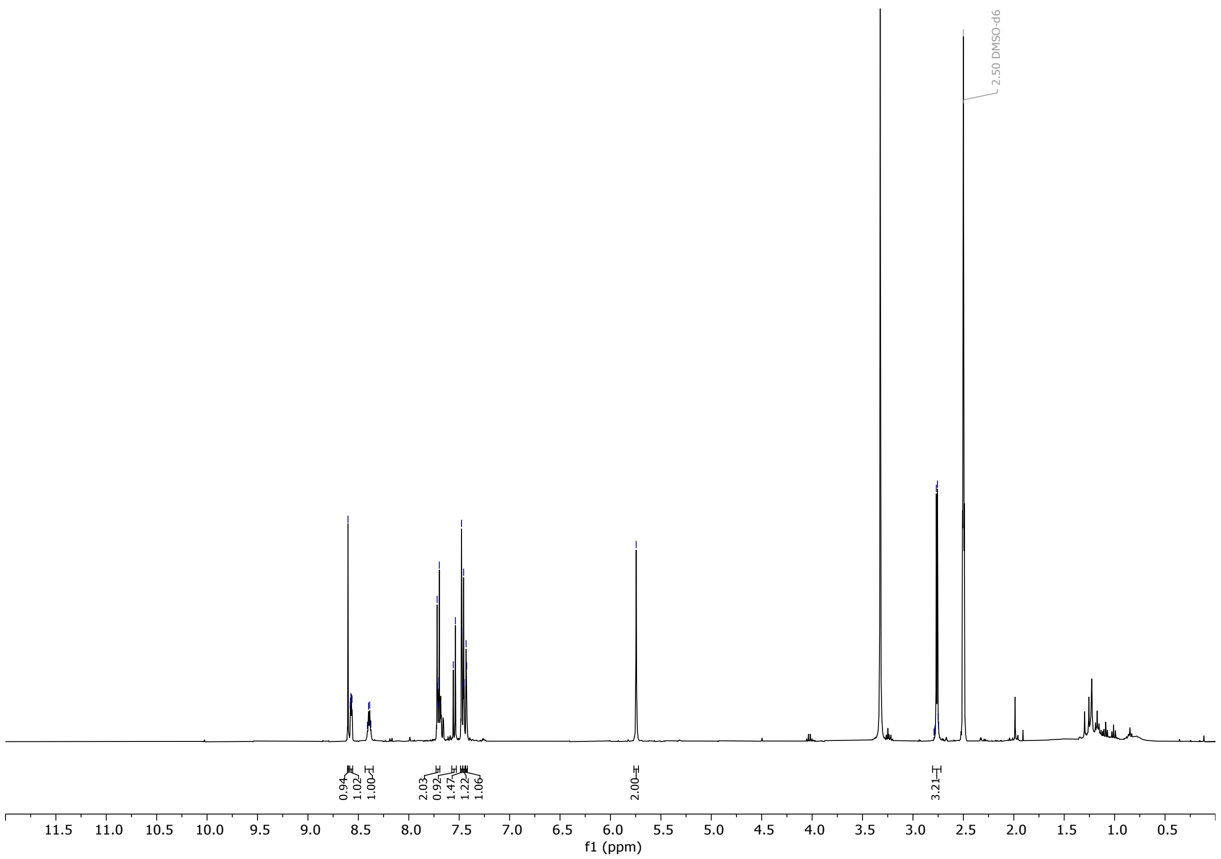
2.50 DMSO-d6
0.94
1.02
1.00
2.03
0.92
1.47
1.22
1.06
2.00
3.21
f1 (ppm)

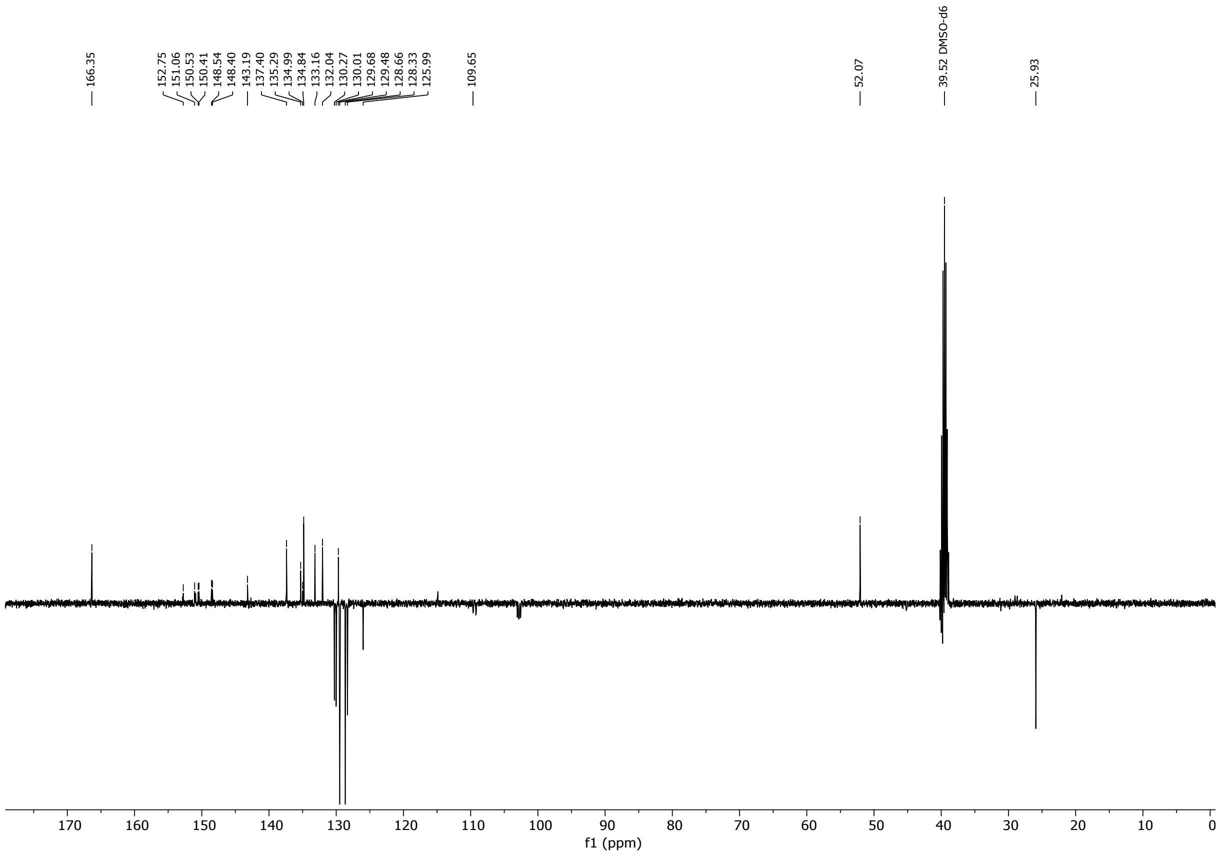
166.35
152.75
151.06
150.53
150.41
148.54
148.40
143.19
137.40
135.29
134.99
134.84
133.16
132.04
130.27
130.01
129.68
129.48
128.66
128.33
125.99
109.65
52.07
39.52 DMSO-d6
25.93
f1 (ppm)

**Cmp 35**

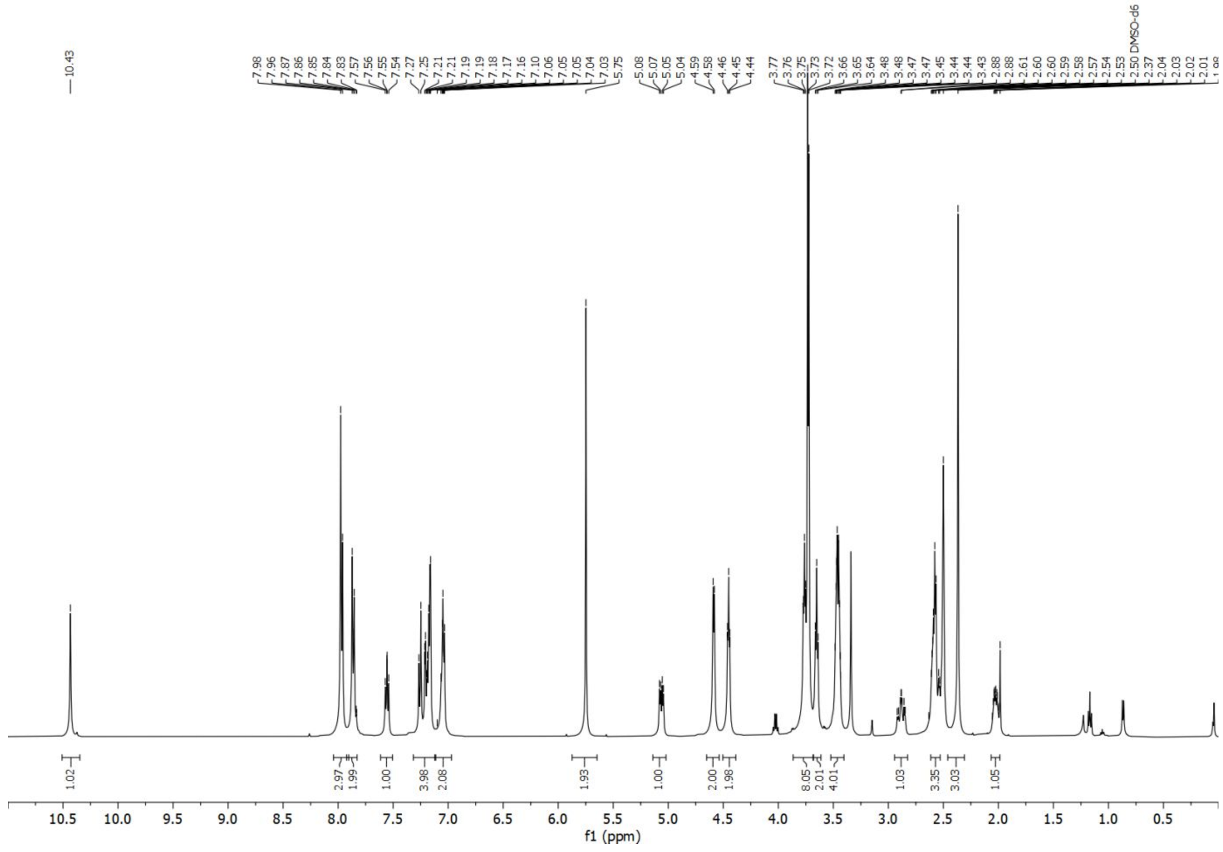

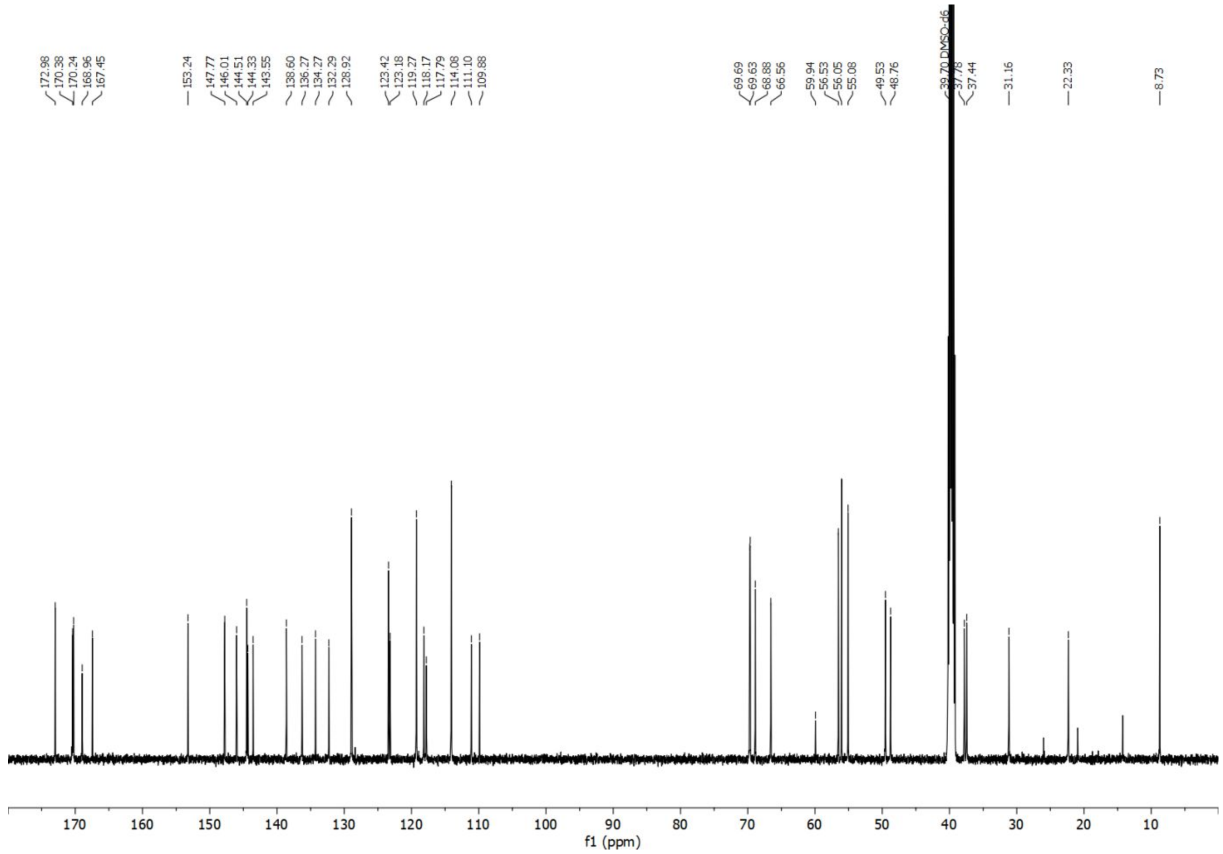

**Cmp 36**

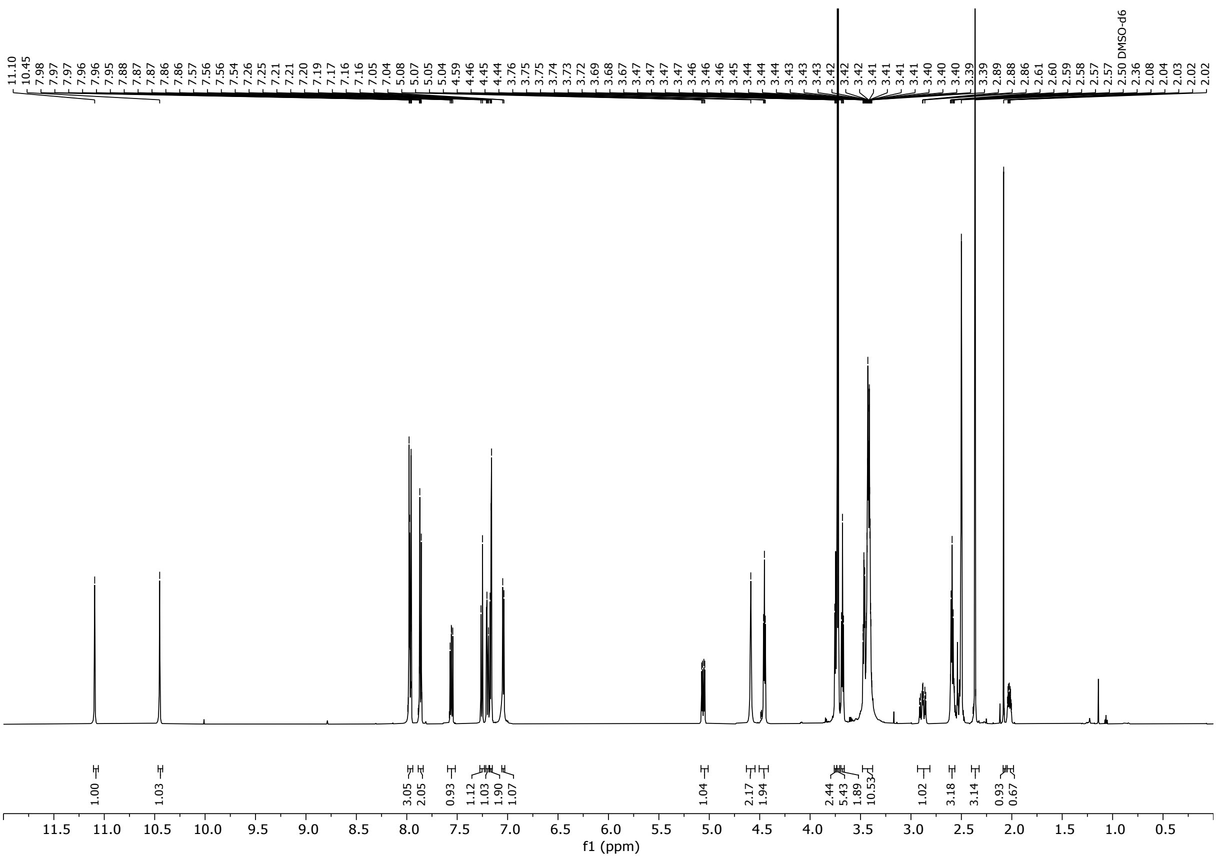

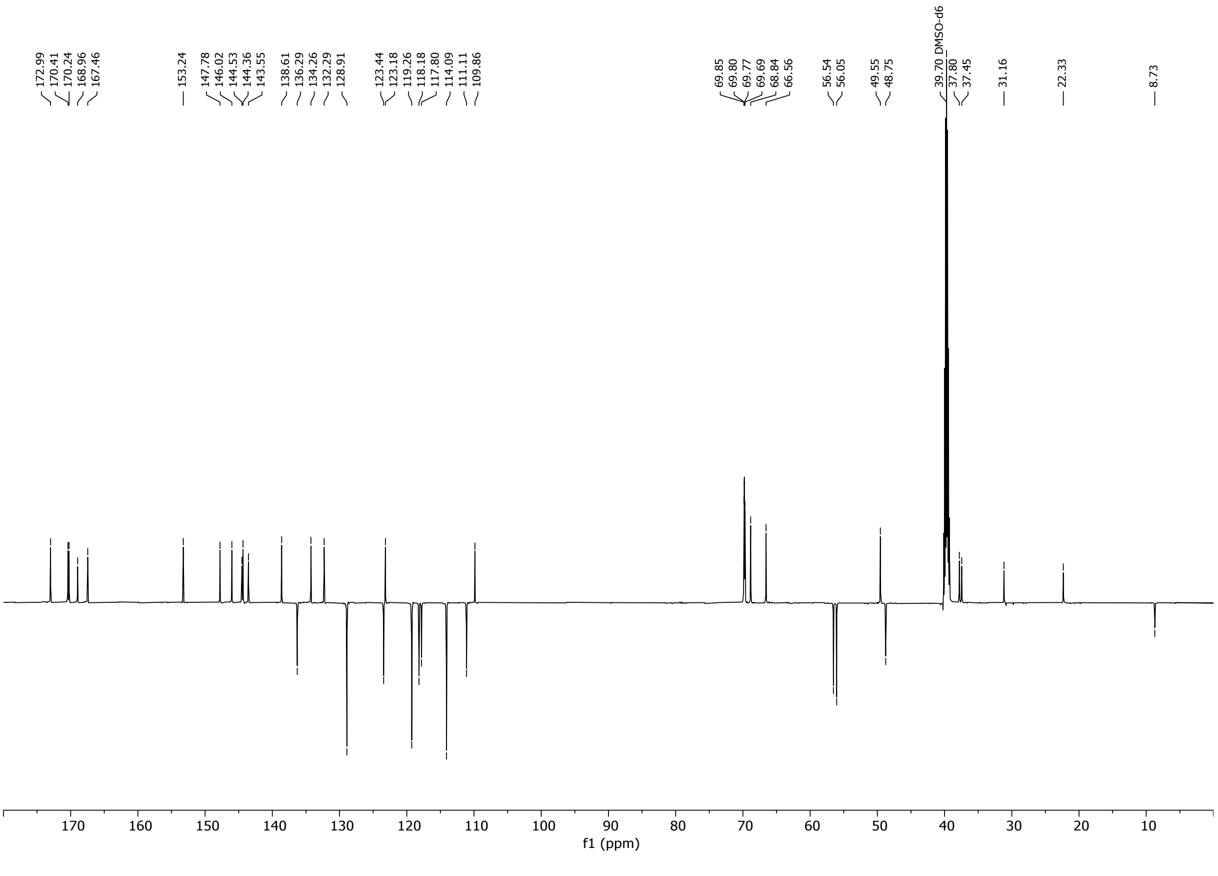

**Cmp 37**

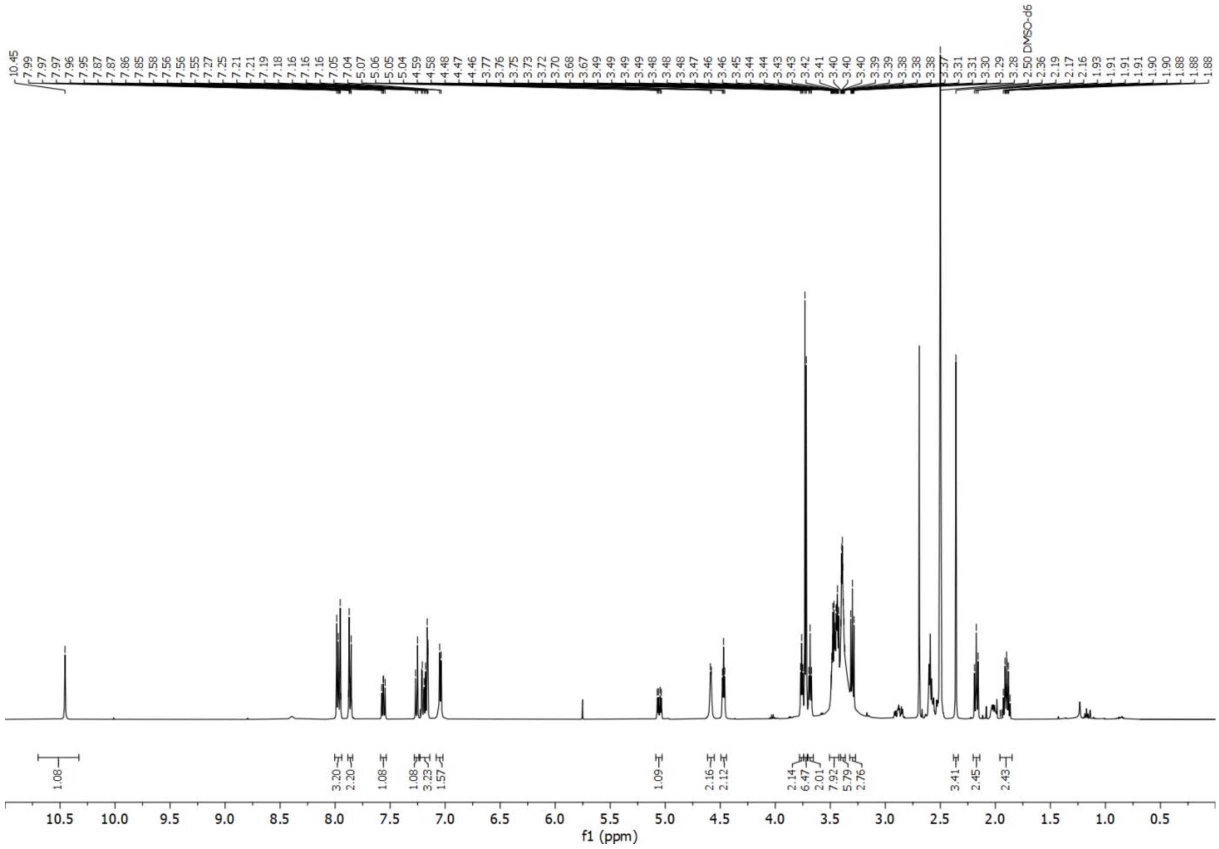


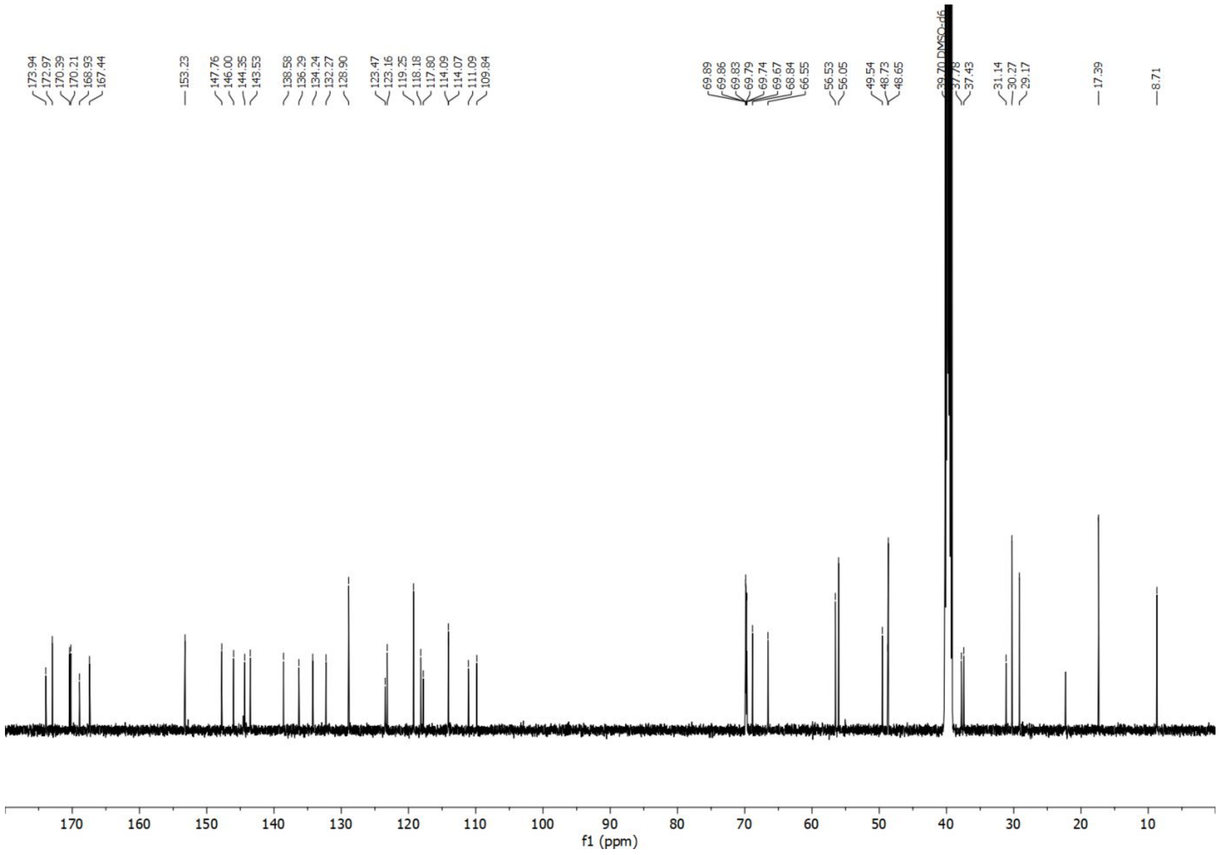


**Cmp S15**

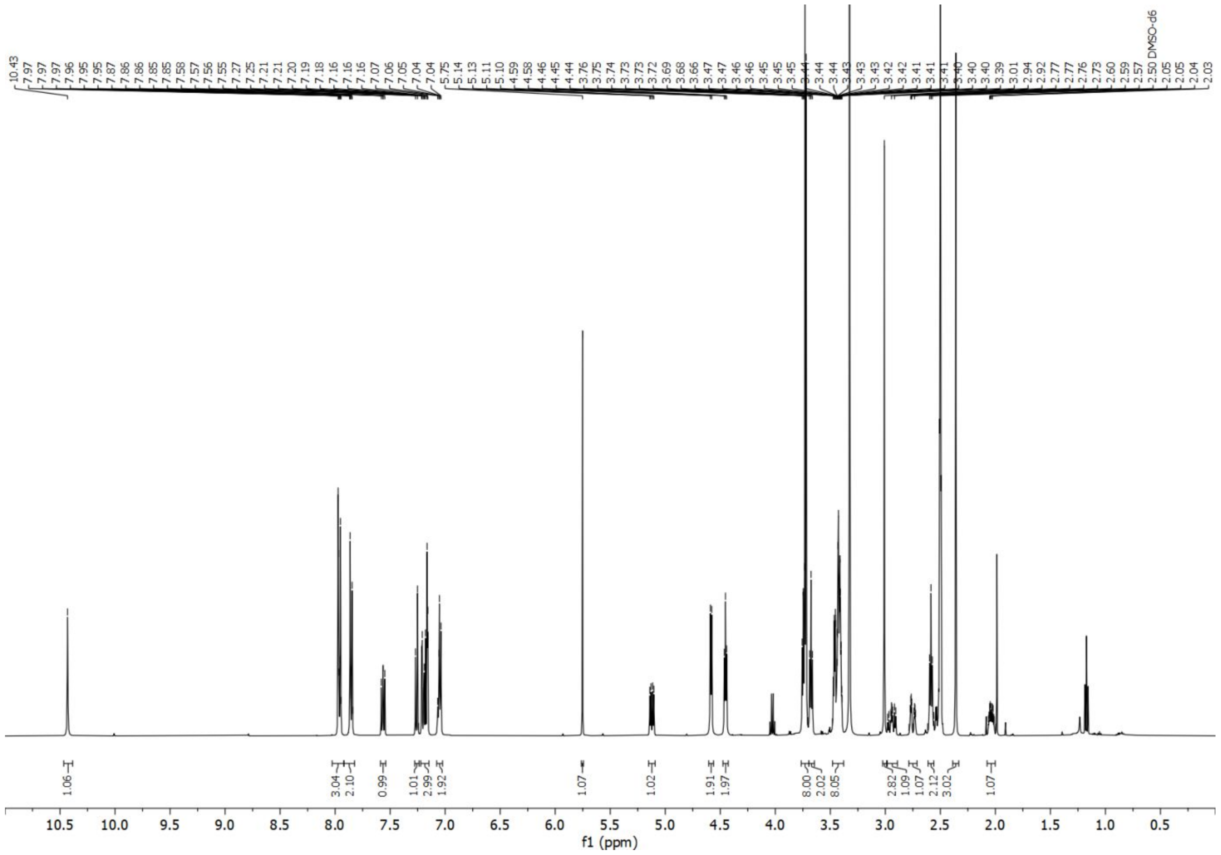
10.43
7.97
7.97
7.97
7.96
7.95
7.95
7.87
7.86
7.86
7.85
7.85
7.58
7.57
7.56
7.55
7.27
7.25
7.21
7.21
7.20
7.19
7.18
7.16
7.16
7.16
7.07
7.06
7.05
7.04
7.04
5.75
5.14
5.13
5.11
5.10
4.59
4.58
4.46
4.45
4.44
3.76
3.75
3.74
3.73
3.73
3.72
3.69
3.68
3.66
3.47
3.47
3.46
3.46
3.45
3.45
3.45
3.44
3.44
3.44
3.43
3.43
3.43
3.42
3.42
3.41
3.41
3.41
3.41
3.40
3.40
3.40
3.39
3.01
2.94
2.92
2.77
2.77
2.76
2.73
2.60
2.59
2.57
2.50 DMSO-d6
2.05
2.05
2.04
2.03
1.06
3.04
2.10
0.99
1.01
2.99
1.92
1.07
1.02
1.91
1.97
8.00
2.02
8.05
2.82
1.09
1.07
2.12
3.02
1.07
10.5
10.0
9.5
9.0
8.5
8.0
7.5
7.0
6.5
6.0
5.5
5.0
4.5
4.0
3.5
3.0
2.5
2.0
1.5
1.0
0.5
f1 (ppm)

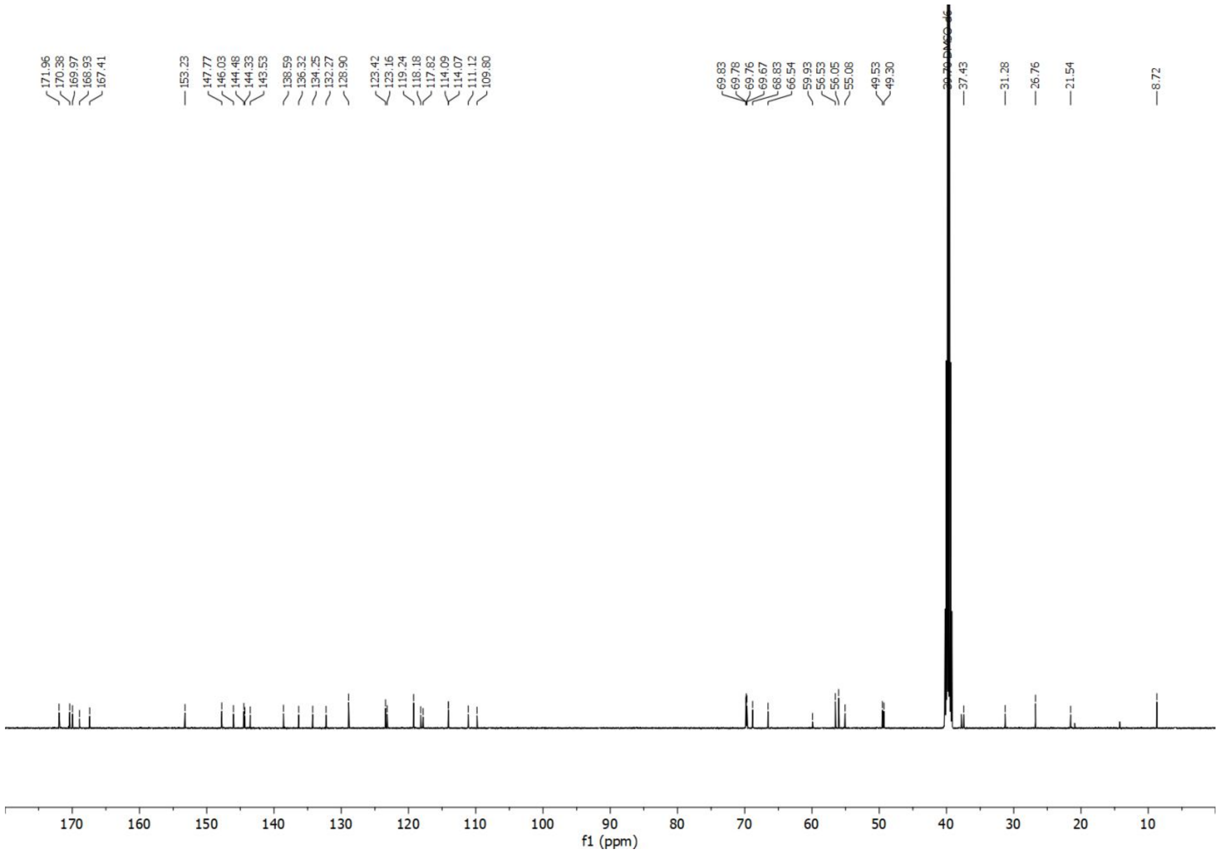
171.96
170.38
169.97
168.93
167.41
153.23
147.77
146.03
144.48
144.33
143.53
138.59
136.32
134.25
132.27
128.90
123.42
123.16
119.24
118.18
117.82
114.09
114.07
111.12
109.80
69.83
69.78
69.76
69.67
68.83
66.54
59.93
56.53
56.05
55.08
49.53
49.30
DMSO-d6
37.43
31.28
26.76
21.54
8.72
170
160
150
140
130
120
110
100
90
80
70
60
50
40
30
20
10
f1 (ppm)

**Table S5.** DigiWest antibody list

| Antigen | Modification Site | Supplier | Product # | Species | MW (kDa) | Dilution | RRID |
|---|---|---|---|---|---|---|---|
| AMPK alpha | | Cell Signaling | 2532 | rb | 62 | 200 | AB_330331 |
| AMPK alpha - phospho | Thr172 | Cell Signaling | 2535 | rb | 62 | 200 | AB_331250 |
| Bak | | Cell Signaling | 3814 | rb | 25 | 200 | AB_2290287 |
| Bcl2 | | Cell Signaling | 4223 | rb | 26 | 200 | AB_1903909 |
| Bcl-xL | | Cell Signaling | 2764 | rb | 30 | 200 | AB_2228008 |
| beta-Catenin | | Cell Signaling | 8480 | rb | 92 | 200 | AB_11127855 |
| beta-Catenin - phospho | Ser33/Ser37/Thr41 | Cell Signaling | 9561 | rb | 92 | 200 | AB_331729 |
| beta-Catenin - phospho | Ser675 | Cell Signaling | 9567 | rb | 92 | 200 | AB_2088241 |
| C/EBP alpha | | Cell Signaling | 8178 | rb | 42, 28 | 200 | AB_11178517 |
| C/EBP beta | | Cell Signaling | 3087 | rb | 49-45 | 200 | AB_2078052 |
| C/EBP beta - phospho | Thr235 | Cell Signaling | 3084 | rb | 38, 36, 19 | 200 | AB_2260359 |
| Caspase 3 | | Cell Signaling | 9662 | rb | 35, 19, 17 | 200 | AB_331439 |
| CDK4 | | Cell Signaling | 12790 | rb | 30 | 200 | AB_2631166 |
| c-Jun | | Cell Signaling | 9165 | rb | 48, 43 | 200 | AB_2130165 |
| c-Jun - phospho | Ser63 | Cell Signaling | 2361 | rb | 48 | 200 | AB_490908 |
| CREB | | Cell Signaling | 9197 | rb | 43 | 200 | AB_331277 |
| CREB - phospho | Ser133 | Cell Signaling | 9198 | rb | 43 | 200 | AB_2561044 |
| Cyclin D1 | | Cell Signaling | 55506 | rb | 36 | 200 | AB_2827374 |
| Cyp2B6 | | Thermo Scientific | PA5-35032 | rb | 70 | 200 | AB_2552342 |
| Cyp2C8 | | Puracyp | Hu-A004 | rb | 55 | 50 | n.a. |
| Cyp2E1 | | Acris | AP00032PU-N | rb | 58 | 200 | AB_979386 |
| Cyp3A4 | | BD Biosciences | 458234 | rb | 57 | 200 | AB_712948 |
| Cyp7A1 | | biorbyt | orb5022 | rb | 55 | 50 | AB_10927729 |
| Erk1/2 (MAPK p44/42) | | Cell Signaling | 4695 | rb | 44, 42 | 200 | AB_390779 |
| Erk1/2 (MAPK p44/42) - phospho | Thr202/Tyr204 | Cell Signaling | 9101 | rb | 44, 42 | 200 | AB_331646 |
| FoxO1 (FKHR) | | Cell Signaling | 9462 | rb | 82 | 200 | AB_2893250 |
| FoxO1 (FKHR) - phospho | Ser256 | Cell Signaling | 9461 | rb | 82 | 200 | AB_329831 |
| FoxO1/O3a/O4 - phospho | Thr24/Thr32/Thr28 | Cell Signaling | 2599 | rb | 95, 82-78, 65 | 200 | AB_2106814 |
| FoxO3a | | Cell Signaling | 2497 | rb | 97-82 | 200 | AB_836876 |
| GADD45B | | abcam (Epitomics) | ab128920 (5833-1) | rb | 18 | 1000 | AB_11150431 |
| Glucose 6 phosphatase alpha | | biorbyt | orb6097 | rb | 40 | 50 | AB_10929668 |
| GSK3 alpha | | Cell Signaling | 9338 | rb | 51 | 200 | AB_2114897 |
| GSK3 alpha/beta - phospho | Ser21/Ser9 | Cell Signaling | 8566 | rb | 51, 46 | 200 | AB_10860069 |
| HNF-1 alpha (HNF-1A) | | abcam | ab96777 | rb | 67 | 200 | AB_10679303 |
| HNF-4 alpha (HNF-4A) | | biorbyt | orb40132 | rb | 52 | 200 | AB_10993027 |

| | | | | | | | |
|---|---|---|---|---|---|---|---|
| HNF-4 alpha (HNF-4A) - phospho | Ser142 | biorbyt | orb34827 | rb | 52 | 200 | AB_10994759 |
| Ki-67 | | USBiological | K1700-05D | rb | 395, 345 | 50 | n.a. |
| Lipoprotein lipase | | abcam (Epitomics) | ab172953 (8021-1) | rb | 53 | 1000 | n.a. |
| mTOR (FRAP) | | Cell Signaling | 2983 | rb | 289 | 200 | AB_2105622 |
| mTOR (FRAP)-phospho | Ser2448 | Cell Signaling | 5536 | rb | 289 | 100 | AB_10691552 |
| NF-κB p65 | | abcam (Epitomics) | ab76311 (2229-1) | rb | 70 | 1000 | AB_2179019 |
| NF-κB p65 - phospho | Ser468 | Cell Signaling | 3039 | rb | 65 | 200 | AB_330579 |
| P21 - phospho | Thr145 | Invitrogen | PA512646 | rb | 18 | 200 | AB_10979470 |
| p21 (Waf1, Cip1, CDKN1A) | | Cell Signaling | 2947 | rb | 21 | 200 | AB_823586 |
| p38 MAPK | | Cell Signaling | 9212 | rb | 43 | 200 | AB_330713 |
| p38 MAPK - phospho | Thr180/Tyr182 | Cell Signaling | 4511 | rb | 43 | 200 | AB_2139682 |
| PKA C alpha/beta/gamma - phospho | Thr197 | Cell Signaling | 4781 | rb | 42 | 200 | AB_2300165 |
| PKC (pan) - phospho | Ser660 | Cell Signaling | 9371 | rb | 85, 82, 80, 78 | 200 | AB_2168219 |
| PPAR alpha | | abcam | ab8934 | rb | 52 | 200 | AB_306869 |
| PPAR gamma | | biorbyt | orb11291 | rb | 52 | 200 | AB_10748918 |
| PXR (NR1I2) | | Biorbyt | orb131805 | rb | 50, 45 | 200 | n.a. |
| SGK1 | | Cell Signaling | 12103 | rb | 60-45 | 200 | AB_2687476 |
| SRC-3 (NCoA3, AIB1, TRAM1) - phospho | Thr24 | Cell Signaling | 2979 | rb | 160 | 200 | AB_2267107 |
| STAT 1 | | Cell Signaling | 9175 | rb | 91, 84 | 200 | AB_2197984 |
| STAT 1 - phospho | Tyr701 | Cell Signaling | 9167 | rb | 91, 84 | 200 | AB_561284 |
| STAT 3 | | Cell Signaling | 4904 | rb | 86, 79 | 200 | AB_331269 |
| STAT 3 - phospho | Ser727 | Cell Signaling | 9134 | rb | 86 | 200 | AB_331589 |

**Table S6.** RT-qPCR primer list

| S.No | Gene | Assay ID | Species | Transcripts | Amplicon | Dye |
|---|---|---|---|---|---|---|
| 1 | CYP3A4 | Hs00604506_m1 | Human | 17 | 119 | FAM-MGB |
| 2 | CYP2B6 | Hs04183483_g1 | | 13 | 63 | |
| 3 | CYP2C9 | Hs04260376_m1 | | 12 | 66 | |
| 4 | ABCB1 | Hs00184500_m1 | | 7 | 67 | |
| 5 | CYP7A1 | Hs00167982_m1 | | 5 | 66 | |
| 6 | G6PC | Hs00609178_m1 | | 5 | 123 | |
| 7 | ABCC4 | Hs00988721_m1 | | 19 | 141 | |
| 8 | SCD1 | Hs01682761_m1 | | 10 | 129 | |
| 9 | ABCG5 | Hs00223686_m1 | | 12 | 60 | |
| 10 | ABCB11 | Hs00184824_m1 | | 12 | 63 | |
| 11 | CYP4A11 | Hs00167961_m1 | | 29 | 115 | |
| 12 | HMGCS2 | Hs00985427_m1 | | 7 | 91 | |

| | | | | | | |
|---|---|---|---|---|---|---|
| 13 | ACACA | Hs01046047_m1 | | 17 | 65 | |
| 14 | PCNA | Hs00427214_g1 | | 8 | 138 | |
| 15 | FASN | Hs01005622_m1 | | 6 | 62 | |
| 16 | PCK1 | Hs00159918_m1 | | 7 | 81 | |
| 17 | FOXM1 | Hs01073586_m1 | | 22 | 77 | |
| 18 | MKI67 | Hs04260396_g1 | | 8 | 64 | |
| 19 | ABCC3 | Hs00978452_m1 | | 24 | 64 | |
| 20 | ABCG8 | Hs00223690_m1 | | 8 | 63 | |
| 21 | AKR1D1 | Hs00818881_m1 | | 10 | 103 | |
| 22 | AKR1B10 | Hs00252524_m1 | | 8 | 95 | |
| 23 | CYP2C8 | hs02383390_s1 | | 16 | 112 | |
| 24 | GLUT2 | Hs01096908_m1 | | 11 | 65 | |
| 25 | SREBF1 | Hs01088691_m1 | | 15 | 90 | |
| 26 | SREBF2 | Hs01081784_m1 | | 12 | 91 | |
| 27 | SQLE | Hs01123768_m1 | | 12 | 109 | |
| 28 | HMGCR | Hs00168352_m1 | | 11 | 67 | |
| 29 | SHP | Hs00222677_m1 | | 3 | 87 | |
| 30 | CYP1A2 | Hs00167927_m1 | | 10 | 67 | |
| 31 | GSTA | Hs07292464_g1 | | 4 | 163 | |
| 32 | UGT1A1 | Hs01592482_m1 | | 7 | 137 | |
| 33 | SULT2A1 | Hs00234219_m1 | | 12 | 98 | |
| 34 | SULT1E1 | Hs00608272_m1 | | 28 | 77 | |
| 35 | SULT1B1 | Hs01376028_m1 | | 2 | 98 | |
| 36 | ABCC2 | Hs00960489_m1 | | 9 | 62 | |
| 37 | CPT1A1 | Hs00912671_m1 | | 11 | 75 | |
| 38 | CD36 | Hs00354519_m1 | | 31 | 83 | |
| 39 | CYP7B1 | Hs01046431_m1 | | 7 | 169 | |
| 40 | CYP39A1 | Hs00900043_m1 | | 16 | 84 | |
| 41 | HPRT1 | Hs02800695_m1 | | 8 | 82 | |
| 42 | GAPDH | Hs02758991_g1 | | 31 | 93 | |

**SI Reference:**

(1) Leonard, F.; Wajngurt, A.; Tschannen, W.; Klein, M. Unnatural Amino Acids. II. Congeners of DL-3-Carboxy-4-methoxyphenylalanine. Journal of Medicinal Chemistry 1967, 10 (3), 478-481. DOI: 10.1021/jm00315a040.
